# Capsimycin derivatives from *Streptomyces* sp. PH9007 as α‑glucosidase inhibitors and insights into their biosynthetic gene cluster

**DOI:** 10.1186/s12864-026-13269-7

**Published:** 2026-08-18

**Authors:** Zhong Wang, Guangling Wu, Jian Pan, Runze Sun, Shun Cao, Xiaoyan Jin, Yani Zhong, Yuhan Chen, Lizi Cao, Xinlu Chen, Bin Feng, Xueshuang Huang, Hong Pu

**Affiliations:** 1 Hunan Provincial Key Laboratory for Synthetic Biology of Traditional Chinese Medicine, School of Pharmaceutical Sciences, Hunan University of Medicine, Huaihua, Hunan 418000 China; 2 Huaihua Hospital of Traditional Chinese Medicine, Huaihua, 418000 China; 3 College of Pharmacy, Hunan Vocational College of Science and Technology, Changsha, 410004 China; 4 College of Pharmacy, Xinjiang Medical University, Urumchi, Xinjiang 830011 China

**Keywords:** Endophytic *Streptomyces*, Polycyclic tetramate macrolactams, Biosynthetic gene cluster, α-glucosidase inhibitor

## Abstract

**Background:**

The increasing number of individuals with diabetes mellitus (DM) highlights the need to develop new diabetes medications. Polycyclic tetramate macrolactams (PTMs), characterized by a macrocyclic lactam containing an integrated tetramic acid ring, constitute an expanding group of extensively dispersed natural compounds with diverse biological functions.

**Results:**

In this study, two *Streptomyces* strains were isolated from the roots of the medicinal plant *Sinomenium acutum*. Using the Global Natural Products Social (GNPS) web platform, three PTMs, namely, capsimycin (4), capsimycin B (5), and capsimycin G (6), were extracted from *Streptomyces* sp. PH9007, which was isolated from *Sinomenium acutum*. Compounds 4 and 5 inhibited α-glucosidase, with 4 and 5 exhibiting IC_50_ values of 484.6 ± 14.10 μM and 366.9 ± 12.85 μM, respectively. Compound 5 also exhibited antibacterial and antifungal activities, with MICs of 16 µg/mL against MRSA, *Staphylococcus aureus* ATCC 29213 and *Candida albicans* CMCC 98001. The results of fluorescence quenching, UV–visible spectroscopy, molecular docking and molecular dynamics simulations of 5 with α-glucosidase indicated that this compound binds better with α-glucosidase. Whole-genome sequencing of PH9007 revealed a 6, 339, 217 bp chromosome and two plasmids (83, 532 bp and 167, 087 bp) containing 25 biosynthetic gene clusters (BGCs), including one exhibiting 56% sequence similarity to the ikarugamycin BGC. Furthermore, bioinformatics analysis revealed that the ikarugamycin BGC is widely distributed in microorganisms.

**Conclusions:**

Overall, this study highlights that PTM-type compounds may serve as promising compounds for the further development of α-glucosidase inhibitors and lays the foundation for research on their biosynthesis.

**Supplementary Information:**

The online version contains supplementary material available at https://doi.org/10.1186/s12864-026-13269-7.

## Background

Diabetes mellitus (DM) is a chronic metabolic disorder characterized by persistent hyperglycemia and represents a major global health challenge. DM ranks as the ninth major cause of death worldwide [1]. Type 2 DM (T2DM) accounts for more than 90% of all diabetes cases [2]. α-Glucosidase inhibitors play a crucial role in managing T2DM by delaying carbohydrate digestion and reducing postprandial glucose levels. Currently, only three such inhibitors—acarbose, miglitol, and voglibose—are clinically available [3]. Despite their clinical effectiveness, limitations associated with these inhibitors have encouraged the search for structurally diverse α-glucosidase inhibitors with promising biological properties.

**Fig. 1:**
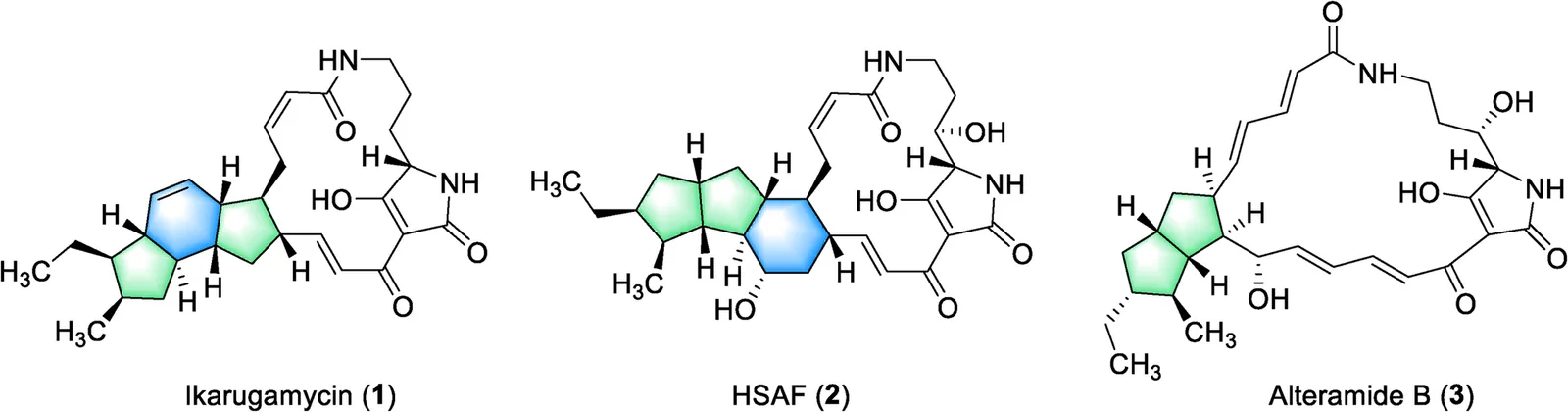
Examples of PTM-type compounds include ikarugamycin (**1**), HSAF (**2**), and alteramide B (**3**)

Natural products, particularly microbial secondary metabolites, have historically served as an important source of bioactive molecules for drug discovery. Among these metabolites, polycyclic tetramate macrolactams (PTMs) represent an expanding class of structurally diverse natural products with various biological activities [4]. Structurally, PTMs consist of a macrocyclic lactam ring containing a tetramate unit accompanied by a diverse array of stereochemically complex carbocyclic rings, resulting in 5/5, 5/5/6, 5/6/5, and 5/4/6 ring systems. Owing to their structure complexity, relatively simple biosynthetic pathway, and significant biomedical potential, PTMs have garnered considerable attention [5–17]. Representative PTMs, including ikarugamycin, dihydromaltophilin (heat-stable antifungal factor, HSAF) and alteramide B (1–3, Fig. 1) have demonstrated notable antimicrobial [18, 19], antiprotozoal [20], immunoregulatory [21] and cytotoxic properties [22]. However, most studies on PTMs have focused on their antimicrobial and cytotoxic properties, whereas their potential as α-glucosidase inhibitors remains largely unexplored.

The genus *Streptomyces* represents one of the most prolific sources of bioactive natural products and has contributed substantially to the discovery of antibiotics and other therapeutic agents [23]. In particular, endophytic *Streptomyces* associated with medicinal plants represent an underexplored reservoir of structurally diverse secondary metabolites with promising biological activities [24, 25]. Previous studies have demonstrated that metabolites from *Streptomyces* species can exhibit α-glucosidase inhibitory activity [26, 27], suggesting that this microbial resource may provide promising sources for the discovery of enzyme inhibitors Nevertheless, the secondary metabolic potential of endophytic *Streptomyces* associated with medicinal plants, including *Sinomenium acutum*, remains insufficiently characterized, and the α-glucosidase inhibitory activity of PTM-type metabolites has rarely been systematically investigated.

To address these research gaps, this study isolated endophytic *Streptomyces* strains from the roots and stems of *Sinomenium acutum* and employed an integrated strategy combining Global Natural Products Social (GNPS)-based molecular network dereplication, genome mining, bioactivity evaluation and computational simulation to investigate PTM metabolites from *Streptomyces* sp. PH9007. This work not only expands our understanding of the biosynthetic potential of plant-associated endophytic *Streptomyces* but also provides a preliminary foundation for developing PTM scaffolds as novel potential α-glucosidase inhibitors and antimicrobial candidates.

## Results and discussion

### Isolation, molecular identification and phylogenetic analysis of the isolates

**Fig. 2:**
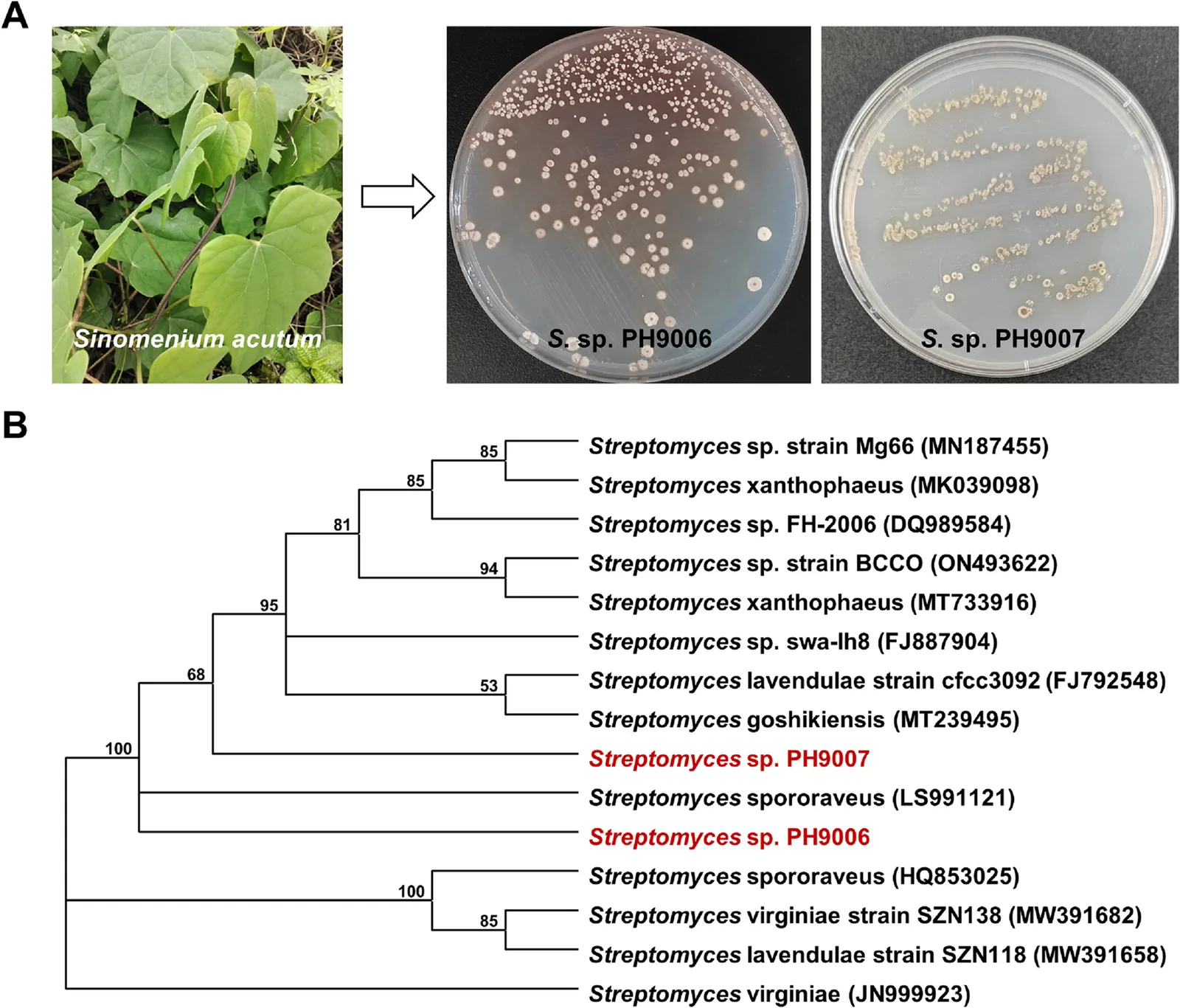
**A** Colony morphology of actinobacteria isolates derived from the roots and stems of *Sinomenium acutum* (Thunb.). **B** Phylogenomic tree showing the relationships between the strains *Streptomyces* sp. PH9006 and *Streptomyces* sp. PH9007 and other members of the genus *Streptomyces*

Two isolates, PH9006 and PH9007 (Fig. 2A), were isolated from the roots of the medicinal plant *S. acutum* (Thunb.) that were collected from a mountain ditch in Zhongfang County, Huaihua city, Hunan Province, China (Fig. 2A), by a series of repeated pure cultures on four types of agar media. The morphological characteristics of the isolates are shown in Fig. 2A. 16S rRNA sequencing was used to identify bioactive isolates, and analysis was performed using the BLASTN tool of the NCBI to construct a phylogenetic tree. The results revealed that the two isolates, PH9006 and PH9007, belong to the *Streptomyces* genus (Fig. 2B). Phylogenetic analysis was conducted on the basis of each sequence of the isolates and the 16S rRNA sequences of related species (Fig. 2B). These results suggested that the species richness and chemical diversity of *Streptomycetes* may be influenced by the complex nature of *S. acutum* (Thunb.). The 16S rRNA data in this study are available in GenBank (accession numbers: PP624363 and PP625129) (Figs. S2–S3).

### Isolation and structural elucidation of compounds 4–6 from *Streptomyces* sp. PH9007

#### Liquid chromatography–tandem mass spectrometry (LC‒MS/MS) and GNPS analyses

**Fig. 3:**
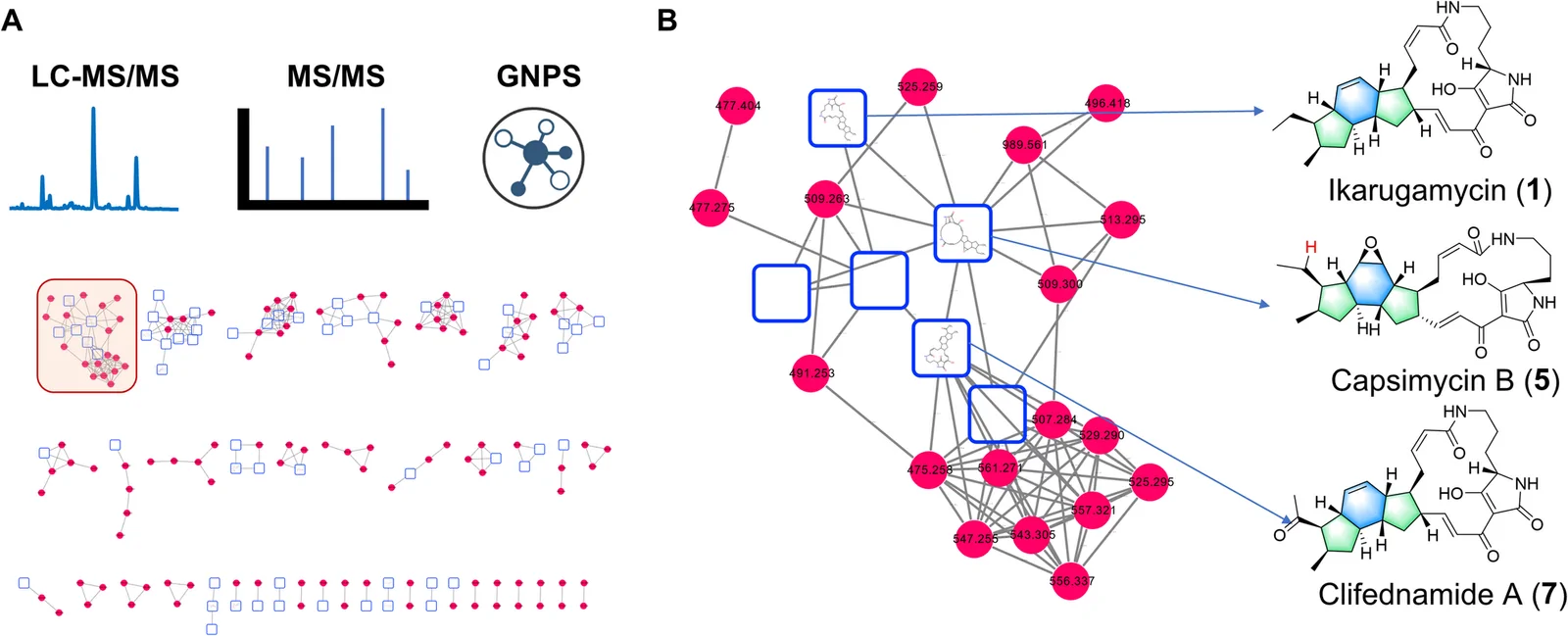
Molecular networking analysis and identification of compounds in the crude extract derived from *Streptomyces* sp. PH9007. **A** Molecular network analysis of the crude extract and clusters of categorized compounds. **B** Magnification of the molecular network of ikarugamycin

After *Streptomyces* PH9006 and PH9007 were fermented in six different media (Table S1, Fig. S1), the secondary metabolites of *Streptomyces* PH9007 cultured in ISP4 medium were analyzed using GNPS. The LC‒MS/MS data of the crude extract obtained using ethyl acetate and methanol were analyzed using the GNPS web platform (https://gnps.ucsd.edu, accessed on 21 February 2025). The data from the crude extract were integrated by the MolNetEnhancer workflow on the GNPS web platform, which automatically determined the chemical class of each cluster (Fig. 3A). Among the clusters, Cluster I, represented by the precursor ion at *m/z* 479.290 [M + H]^+^, was unequivocally annotated as ikarugamycin (1) (Fig. 3B). In addition, the precursor ions at *m/z* 495.284 [M + H]^+^ and 493.269 [M + H]^+^ were annotated as capsimycin B (5) and clifednamide A (7), respectively (Fig. 3B). This clustering analysis offers strong evidence for the presence of PTMs among the secondary metabolites of *Streptomyces* sp. PH9007 and demonstrates its biosynthetic potential for producing PTMs.

#### Isolation and structural elucidation of compounds 4–6 from *Streptomyces* sp. PH9007

**Fig. 4:**
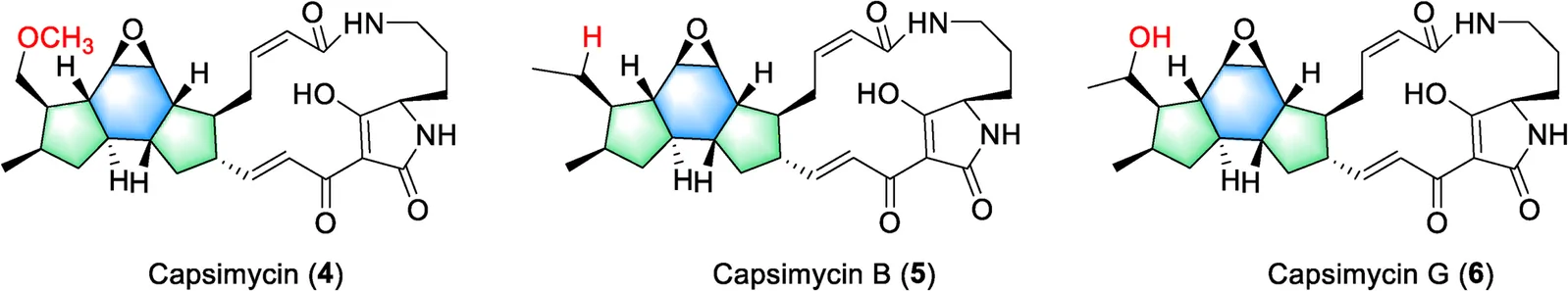
Structures of compounds 4*–*6 isolated from the endophytic *Streptomyces* sp. PH9007

On the basis of the GNPS results, *Streptomyces* sp. PH9007 was subjected to large-scale fermentation (95 L), which led to the production and isolation of compounds 4–6 (Fig. 4). The crude extracts of *Streptomyces* sp. PH9007 were fractionated using silica gel and MCI adsorption resin chromatography, as well as semipreparative HPLC, to yield compounds 4–6. Compound 4 was isolated as a white powder. High-resolution electrospray ionization mass spectrometry (HRESIMS) revealed its molecular formula to be C_30_H_41_N_2_O_6_ on the basis of the molecular ion [M + H]^+^ 525.29572 (calcd, 525.29591) (Fig. S12). The nuclear magnetic resonance (NMR) data of 4 are almost identical to those of capsimycin, which was isolated from mangrove-derived *Streptomyces xiamenensis* 318 (Figs. S4–S11; Table S2) [28]. Compound 4 was identified as capsimycin.

Compound 5 was isolated as a white powder and exhibited UV absorption similar to that of 4, suggesting that these compounds might have a similar molecular skeleton. HRESIMS analysis revealed its molecular formula to be C_29_H_39_N_2_O_5_ on the basis of the molecular ion [M + H]^+^ 495.28433 (calcd, 495.28535) (Fig. S22). The 1D and 2D NMR spectra of 5 further revealed that 5 is the previously isolated capsimycin B from *S. xiamenensis* 318 (Figs. S14–S21; Table S3) [28]. Compound 5 was identified as capsimycin B.

Compound 6 was also isolated as a white powder. HRESIMS revealed its molecular formula to be C_29_H_39_N_2_O_6_ on the basis of the molecular ion [M + H]^+^ 511.27966 (calcd, 511.28026) (Fig. S29). The NMR spectra revealed that 6 was capsimycin G, which was previously isolated from *S. xiamenensis* 318 (Figs. S24–S28; Table S4) [28]. Compound 6 was identified as capsimycin G.

### Bioactivity evaluation, molecular docking studies, and molecular dynamic simulations

#### α-Glucosidase inhibition

The α-glucosidase inhibitory activities of compounds 4*–*6 were evaluated. Compounds 4 and 5 showed significant inhibitory effects, with IC_50_ values of 484.6 ± 14.10 μM and 366.9 ± 12.85 μM, respectively, outperforming the positive control acarbose (IC_50_ = 671.5 ± 0.17 μM) (Table S7, Fig. S31). These results demonstrate that the inhibitory activity of compound 5 against α-glucosidase was approximately twice that of acarbose.

#### Fluorescence quenching

**Fig. 5:**
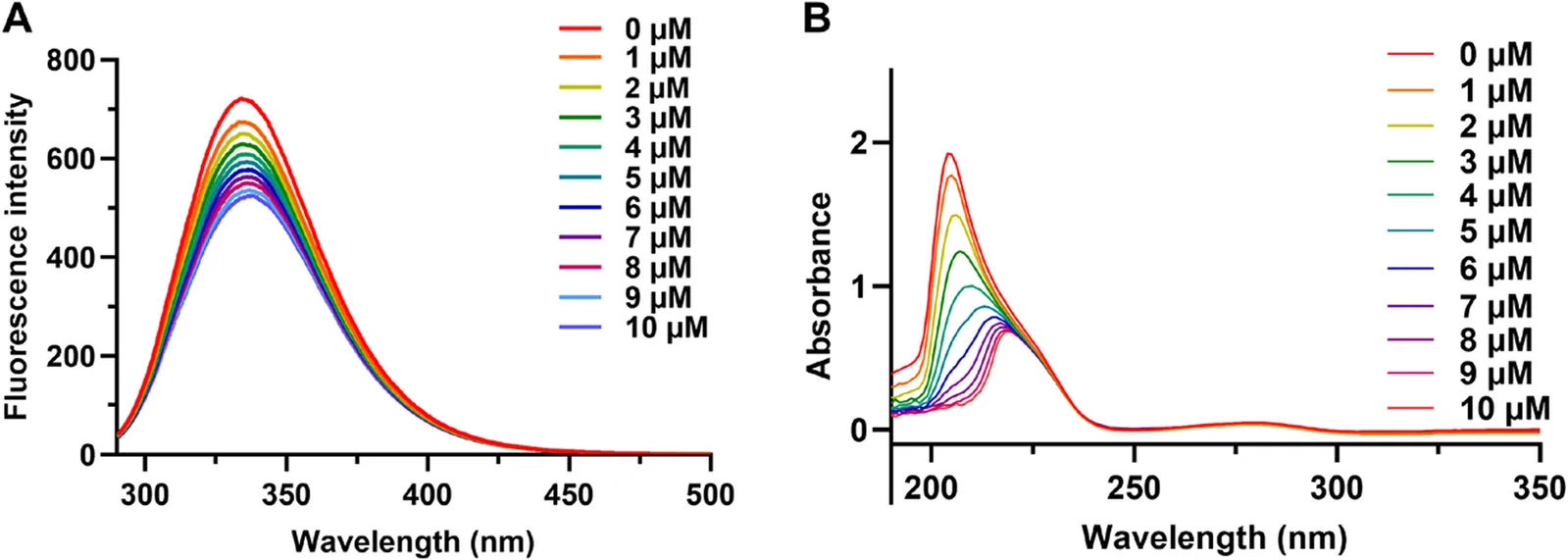
**A** Changes in the fluorescence intensity of α-glucosidase with increasing concentrations of 5 at 298 K. **B** Changes in the absorbance of α-glucosidase with increasing concentrations of 5

To clarify the mechanism by which 5 inhibits α-glucosidase activity, the changes in the fluorescence spectrum of α-glucosidase in the presence of 5 were examined. The fluorescence intensity progressively decreased as the concentration of 5 increased at 298 K, as illustrated in Fig. 5A. These findings suggest that the binding of compound 5 to α-glucosidase could quench the endogenous fluorescence of the enzyme.

#### UV–visible (UV–Vis) spectroscopy

UV–Vis spectroscopy was used to examine the conformational changes in the enzyme and to further clarify the fluorescence quenching mechanism of 5 [29]. As shown in Fig. 5B, α-glucosidase has two prominent UV absorption peaks at 279 and 205 nm. The peak at 279 nm is attributed to the π → π* electron transitions of amino acid residues such as phenylalanine (Phe), tyrosine (Tyr), and tryptophan (Trp), whereas the signal at 205 nm results primarily from the n → π and π → π transitions of peptide bonds (-CO-NH-). The intensity of the peak at 205 nm was much greater than that of the peak at 279 nm. With the incremental addition of compound 5, the intensity of the absorption peak at 205 nm consistently decreased, strongly suggesting the development of an enzyme–inhibitor complex between 5 and α-glucosidase. Furthermore, the peak absorption of the peptide bonds redshifted from 205 nm to 211 nm. This suggested that the microenvironment of the associated peptide bonds was altered in terms of either polarity or steric hindrance, leading to a modification in the energy of the chromophore. As the amount of 5 added increased, the light absorption intensity at 260 nm markedly increased. This increase may be attributable to the establishment of hydrogen bonds between the inhibitor and enzyme, which enhances the density of π-electron clouds surrounding aromatic amino acid residues and increases the UV absorption intensity [30]. These data suggested an interaction between 5 and α-glucosidase. Moreover, a comparison of the ultraviolet spectra of the free enzyme and the enzyme–5 complex revealed incomplete overlap, further substantiating the static quenching mechanism of α-glucosidase by 5. This outcome aligns with prior fluorescence quenching studies.

#### Molecular docking

**Fig. 6:**
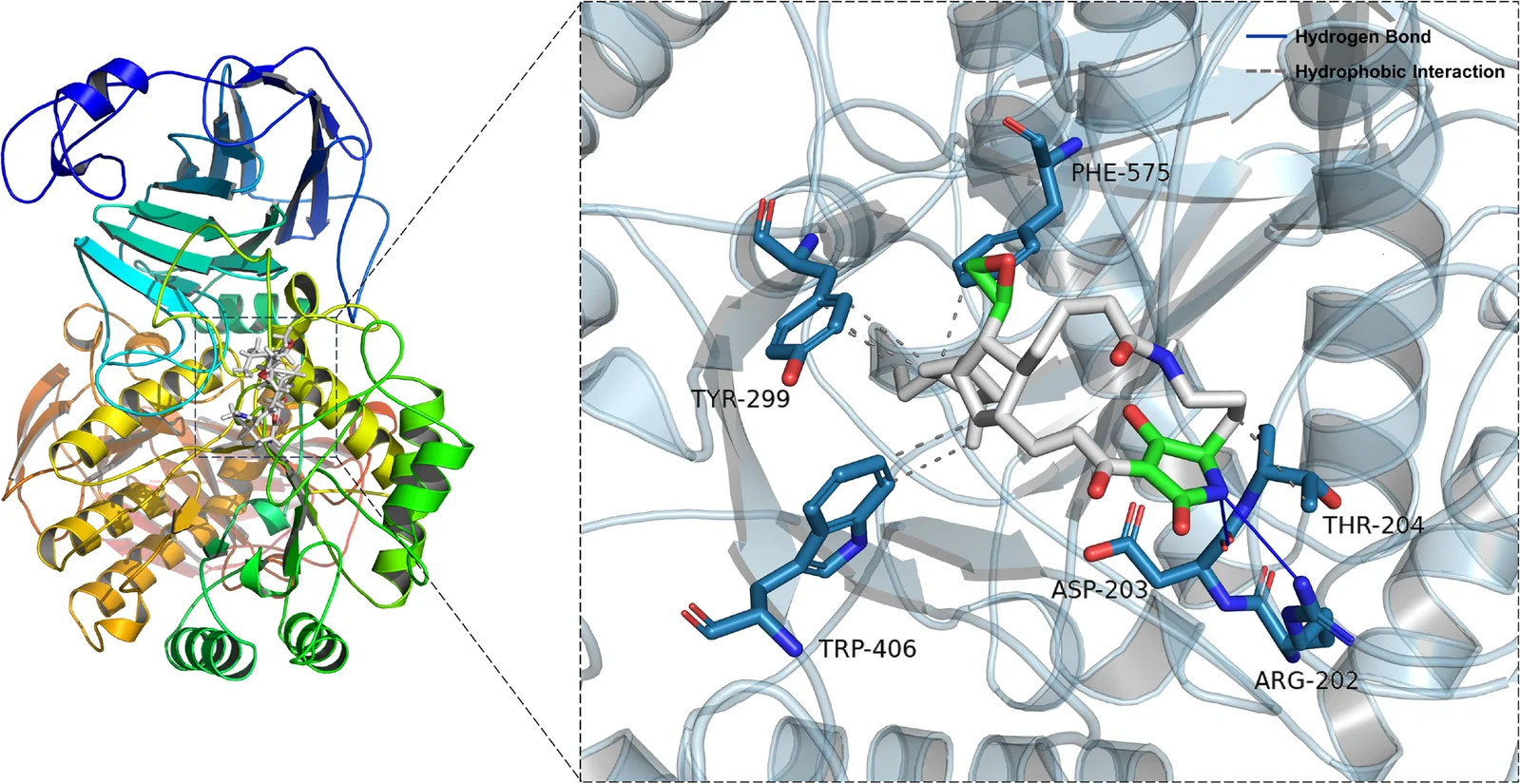
Docking pose and interactions of 5 with α-glucosidase (PDB ID: 2QMJ)

Molecular docking studies (AutoDock Vina 1.1.2) were conducted to predict the binding modes of 4 and 5 within the α-glucosidase active site (PDB: 2QMJ). As shown in Fig. 6, compound 5 formed two hydrogen bonds with residues Arg-202 and Asp-203 and engaged in multiple hydrophobic interactions with residues including Tyr-299, Trp-406, Thr-204, and Phe-575. In addition, 4 formed six hydrogen bonds with the Arg-202, Asp 203 and Thr-205 residues and six hydrophobic interactions with Thr-204, Tyr-299, Trp-406, Leu-473 and Phe-575. The calculated binding affinities were -8.2, -7.6, and -6.7 kcal/mol for 4, 5, and acarbose, respectively, which showed good correlation with the experimental IC_50_ values; therefore, these compounds may stably bind to α-glucosidase (Table S8). As shown in Fig. S32, fewer hydrogen bonds and hydrophobic interactions were formed between acarbose and α-glucosidase than between compounds 4 and 5 and α-glucosidase. The superior affinity of the PTMs over acarbose is attributed to their distinct interaction profiles.

It should be noted that the α-glucosidase used in the biochemical inhibition assay was derived from *Saccharomyces cerevisiae*, whereas the protein structure used for molecular docking and molecular dynamics simulations was based on the N-terminal subunit of human maltase-glucoamylase (PDB ID: 2QMJ). Although these proteins originate from different species, both are classified within the glycoside hydrolase family 31 (GH31) α-glucosidase family and share conserved catalytic residues and structural features involved in substrate recognition and hydrolysis [31–33]. Therefore, the human maltase-glucoamylase structure (PDB ID: 2QMJ) was employed as a representative structural model of GH31 α-glucosidases to investigate the potential ligand-binding interactions of the identified compounds. Accordingly, the docking and molecular dynamics simulation results should be interpreted as providing structural insights into possible ligand–α-glucosidase interactions rather than a definitive representation of the binding mode in the yeast enzyme. Future studies using experimentally determined structures or reliable homology models of yeast α-glucosidase may further elucidate the binding mechanisms of these inhibitors.

#### Molecular dynamics simulations

**Fig. 7:**
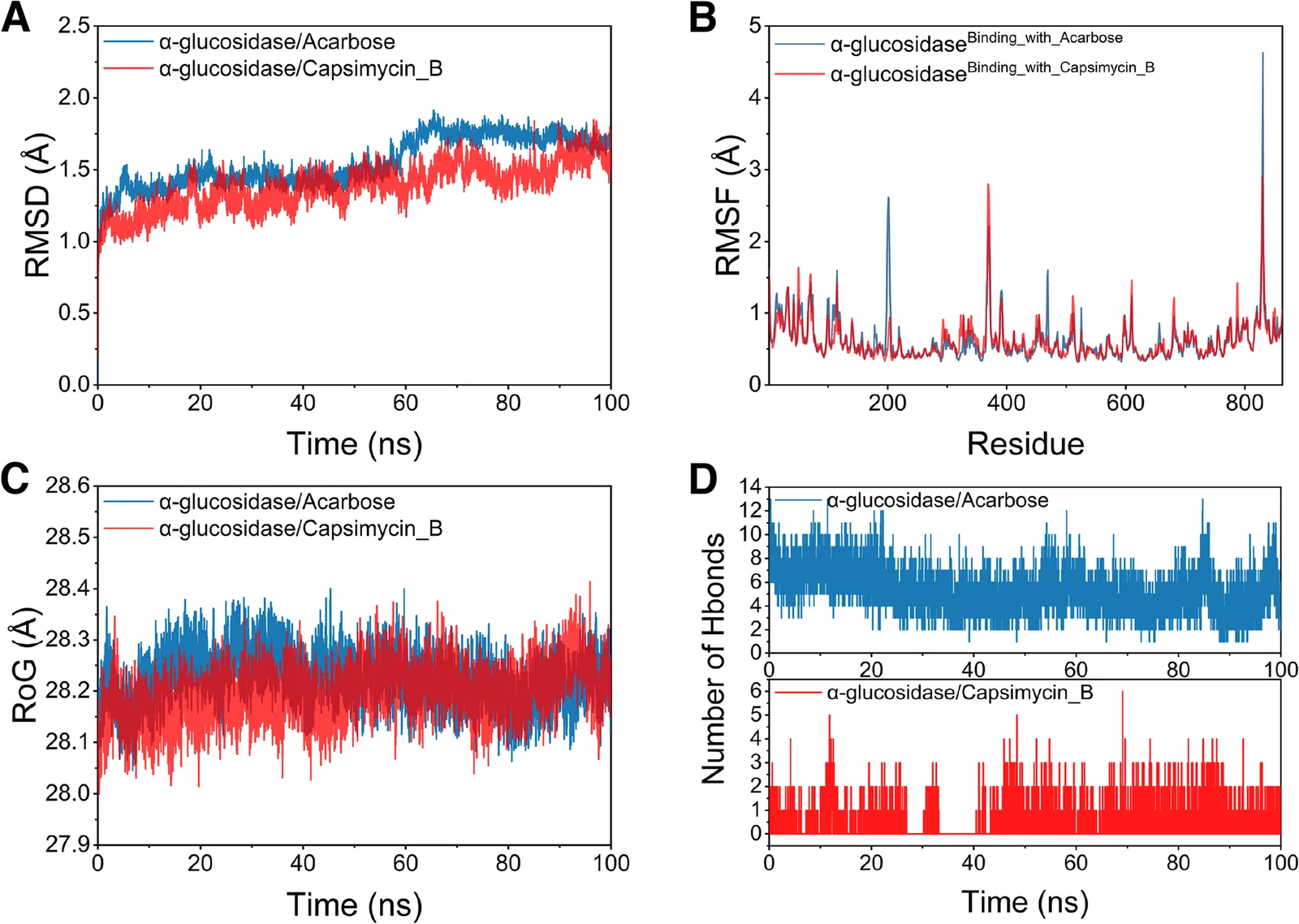
Molecular dynamics simulations of acarbose and 5 with the active pocket of α-glucosidase. **A** RMSD values of the complex and protein backbone systems during the molecular dynamics simulations. **B** Changes in the RMSF values of binding site residues in the free protein and complex systems. **C** Radius of gyration values of the two systems during the molecular dynamics simulations. **D** Numbers of hydrogen bonds during the molecular dynamics simulations

Molecular dynamics simulations were performed under physiological conditions to assess the binding pattern, stability, and interaction mode of 5 with α-glucosidase. The dynamic variations and stability of the complexes were analyzed using RMSD, RMSF, and hydrogen bond metrics, with low RMSD and RMSF values indicating structural stability [34]. As shown in Fig. 7A, the RMSD curves for the simulated α-glucosidase/acarbose and α-glucosidase/capsimycin B complexes converged at approximately 60 ns, with stable fluctuations observed in subsequent simulations. The simulations indicated that α-glucosidase/capsimycin B reached convergence at 10 ns, at which time it exhibited consistent variations in subsequent simulations. The reduced RMSD of the α-glucosidase/capsimycin B complex suggests minimal alterations in the system and enhanced stability after small-molecule binding. As shown in Fig. 7B, all the protein regions exhibited low RMSF values after ligand binding, suggesting increased rigidity within the protein core. Proteins exhibit increased stiffness upon binding with inhibitory small molecules. The red and blue lines demonstrate significant overlap, suggesting that the two small molecules exert comparable effects on the protein. The RMSF of the acarbose-bound protein exceeded that of the capsimycin B-bound protein (red line). In comparison to acarbose, capsimycin B exhibited increased inhibition of protein flexibility. In comparison with α-glucosidase/acarbose, α-glucosidase/capsimycin B has a reduced radius of gyration (RoG) and diminished fluctuations throughout the simulation (Fig. 7C). Molecular dynamics simulations based on the human maltase-glucoamylase model indicated that the capsimycin B–protein complex exhibited higher compactness and stability than the acarbose complex under simulated physiological conditions.

The binding energy was calculated using MM-GBSA, a method appropriate for assessing small-molecule interactions with target proteins based on molecular dynamics simulation trajectories. Table S9 presents the binding energies for the α-glucosidase/acarbose and α-glucosidase/capsimycin B complexes, which are -16.77 ± 1.25 and -22.07 ± 3.89 kcal/mol, respectively. A negative value indicates the ligand’s ability to bind to the target protein, whereas a lower value denotes a higher binding affinity. Small molecules clearly bind to proteins. Binding is governed primarily by electrostatic and van der Waals interactions, whereas the contribution of the nonpolar solvation free energy to the total binding energy is minimal.

Hydrogen bonds are among the most robust noncovalent interactions, with an increase in their quantity correlated with enhanced bond strength. Variations in the number of hydrogen bonds between small molecules and proteins were observed during the 100 ns molecular dynamics simulations. A comparison of the α-glucosidase/acarbose and α-glucosidase/capsimycin B complexes (Fig. 7D) indicated that the α-glucosidase/acarbose complex exhibited 5 to 6 hydrogen bonds, whereas the α-glucosidase/capsimycin B complex typically displayed between 1 and 3 hydrogen bonds. Hydrogen bonds play a crucial role in the stable binding of acarbose to α-glucosidase, as the presence of numerous hydrogen bond donors and acceptors in the acarbose structure facilitates the formation of a substantial number of hydrogen bonds. The α-glucosidase/capsimycin B complex exhibited a variable number of hydrogen bonds, ranging from 0 to 6 throughout the simulations, with most snapshots indicating the presence of 1 hydrogen bond. Hydrogen bonding played an important role in the binding of acarbose to α-glucosidase, whereas it had a minimal effect on the binding of capsimycin B to the same enzyme; van der Waals interactions were the primary contributors to the binding of capsimycin B.

#### Antibacterial activity

The antibacterial activities of 4–6 were tested against MRSA, *S. aureus* ATCC 29213, *Klebsiella pneumoniae* ATCC 113 and *Pseudomonas aeruginosa* ATCC 9027. Compound 5 exhibited notable activity against gram-positive bacteria, with MIC values of 16 µg/mL against both MRSA and *S. aureus* ATCC 29213. Compounds 4 and 6 were inactive (MIC > 64 µg/mL) (Table S5, Fig. S33).

#### Antifungal activity

The antifungal activities of 4–6 against *Candida albicans* CMCC 98001 and *Candida parapsilosis* ATCC 22019 were evaluated by the broth microdilution checkerboard method based on the CLSI-M27-A3 standard. Among the tested compounds, compound 5 exhibited antifungal activity, with MIC values of 16 µg/mL against *C. albicans* CMCC 98001 and 32 µg/mL against *C. parapsilosis* ATCC 22019. The MICs of compounds 4 and 6 were greater than 64 µg/mL, indicating no significant inhibitory activity (Table S6).

### Genome characteristics of *Streptomyces* sp. PH9007

**Fig. 8:**
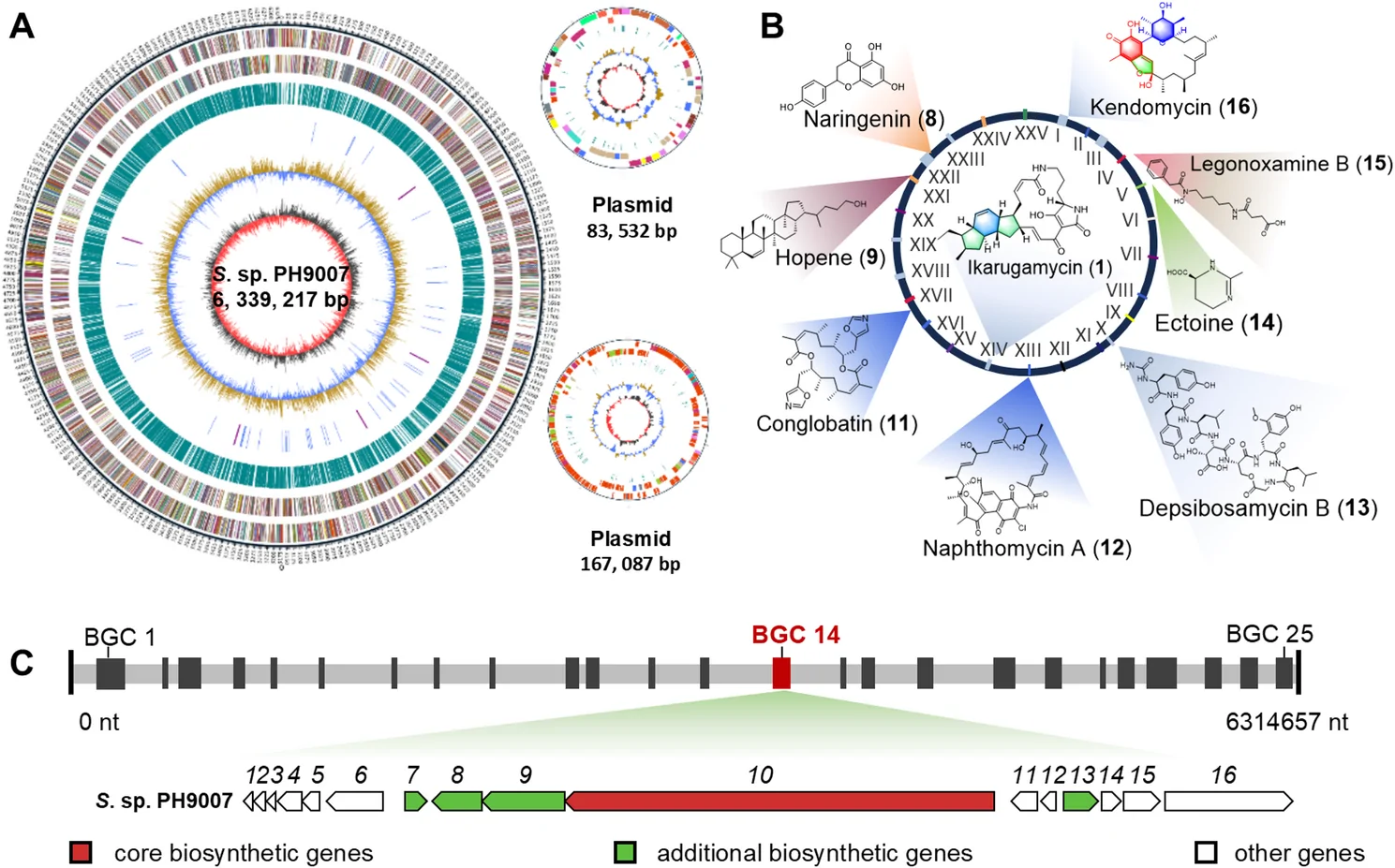
**A** Genome map of *Streptomyces* sp. PH9007. The outermost circle of the genome map identifies the genome size. The second and third circles represent the CDSs on the forward and reverse strands, respectively, and the different colors indicate the different COG functional categories of the CDSs. The fourth circle shows the locations of the rRNAs and tRNAs. The fifth circle represents the GC content. The central circle shows the GC skew values (G − C/G + C) of the third bases of the codons measured throughout the genome. **B** Biosynthetic loci identified in the genome of *Streptomyces* sp. PH9007. BGCs were identified using antiSMASH and manual analysis. The structures of the natural products currently assigned to the BGCs are shown. Ikarugamycin, as the focus of this study, is shown in the center of the circle representing the genome, and previously reported compounds are shown. **C** Ikarugamycin biosynthetic gene cluster (*ika* BGC) in *Streptomyces* sp. PH9007

The unique chemical structure of compound 5, coupled with its significant α-glucosidase inhibitory activity and antibacterial and antifungal effects, prompted us to perform whole-genome sequencing of *Streptomyces* sp. PH9007 to investigate the biosynthetic origin of this class of compounds, particularly compound 5. We analyzed and reported the genome data in this study. The PH9007 genome consists of a 6, 339, 217 bp linear chromosome and two linear plasmids of 83, 532 bp and 167, 087 bp, with an average G + C content of 72.07%. The complete genome was predicted to contain 5, 949 protein-coding genes, 59 rRNA genes, and 15 tRNA genes (Fig. 8A; Table 1).

**Table 1:** General genomic features of *Streptomyces* sp. PH9007

Feature	Chromosome	Plasmid	Plasmid
Genome topology	Linear	Linear	Linear
Assembly size (bp)	6, 339, 217	83, 532	167, 087
G + C content (%)	72.07		
Protein-coding genes	5, 949		
tRNA genes	59		
rRNA genes	15		
Secondary metabolite gene clusters	25	1	1
GenBank accession	JBQRUY000000000		
NCBI BioProject accession	PRJNA1301833		

The genome of *Streptomyces* sp. PH9007 has a considerable number of candidate biosynthetic gene clusters (BGCs) for various metabolites based on in silico analysis. Up to 25 putative BGCs (clusters 1 to 25) for secondary metabolites were unevenly distributed on the 6.38 Mb linear chromosome and were responsible mainly for the production of nonribosomal peptides, transAT-PKS, polyketides, and terpenes, as determined by antiSMASH 7.1.0 and manual inspection. In addition, many other types of BGCs, such as those for siderophores, lanthipeptides, hydrogen-cyanide, ectoines and lasso peptides, were also predicted (Fig. 8B, Table S10). Moreover, through antiSMASH analysis, the putative ikarugamycin BGC (*ika* BGC), which is responsible for the synthesis of ikarugamycin, was predicted in *Streptomyces* sp. PH9007 and is highly similar to the *ika* BGC in *Streptomyces* sp. ZJ306, which contains 16 open reading frames (*orf*s) (Fig. 8C; Table 2). According to the results, *Streptomyces* sp. PH9007 could produce numerous secondary metabolites. Specifically, this isolate has two putative gene clusters that are identical (100% gene similarity) to the reported compound gene clusters, which are for naringenin (8) and ectoine (14); one putative gene cluster that shows high similarity (> 70% gene similarity) to the reported cypemycin gene cluster; and three putative gene clusters that show moderate similarity (40 ~ 70% gene similarity) to the reported ikarugamycin (1), desferrioxamine B (13), legonoxamine B (15) and kendomycin (16) clusters. Nineteen putative gene clusters showed low similarity (< 40% gene similarity) with the reported gene clusters for hopene (9), butyrolactol A, triacsin C and deoxyhangtaimycin, among others, and three putative gene clusters were unknown (Table S10).

**Table 2:** The predicted functions of genes in the *ika* biosynthetic gene cluster from *Streptomyces* sp. PH9007

Gene	aa^a^	Putative function	Protein homolog, origin	ID/SI^b^
PH9007_*orf*1	72	Chitin synthase chsA	Unknown	Unknown
PH9007_*orf*2	85	Pescadillo	Unknown	Unknown
PH9007_*orf*3	66	Cytochrome b5	Query_1849768	53%/63%
PH9007_*orf*4	181	Cytosolic 5′-nucleotidase 1 A	Unknown	Unknown
PH9007_*orf*5	130	NADH dehydrogenase subunit 5	Unknown	Unknown
PH9007_*orf*6	414	Acetate metabolism transcriptional regulator RamB	Unknown	Unknown
PH9007_*orf*7	156	GCN5-related N-acetyltransferase	AJD77026.1 (BGC0001435)	95%/97%
PH9007_ *orf*8	354	NADP-dependent oxidoreductase	IkaC AJD77025.1 (BGC0001435)	97%/98%
PH9007_ *orf*9	610	FAD-dependent oxidoreductase	IkaB AJD77024.1 (BGC0001435)	98%/99%
PH9007_ *orf*10	3124	PKS/NRPS enzyme	IkaA AJD77023.1 (BGC0001435)	98%/98%
PH9007_ *orf*11	193	Hypothetical protein	Unknown	96%/96%
PH9007_ *orf*12	111	Tryptophan synthase α chain	Unknown	78%/77%
PH9007_ *orf*13	251	Deacetylase	AJD77020.1 (BGC0001435)	100%/100%
PH9007_ *orf*14	136	Photosystem I core protein PsaA	Unknown	Unknown
PH9007_ *orf*15	263	Hydroxylase	AJD77019.1 (BGC0001435)	94%/96%
PH9007_ *orf*16	932	β-L-Arabinofuranosidase	Unknown	Unknown

^a^Number of amino acids

^b^ID/SI: identity/similarity

### Ikarugamycin-related PTM BGC is widespread among diverse bacterial strains

**Fig. 9:**
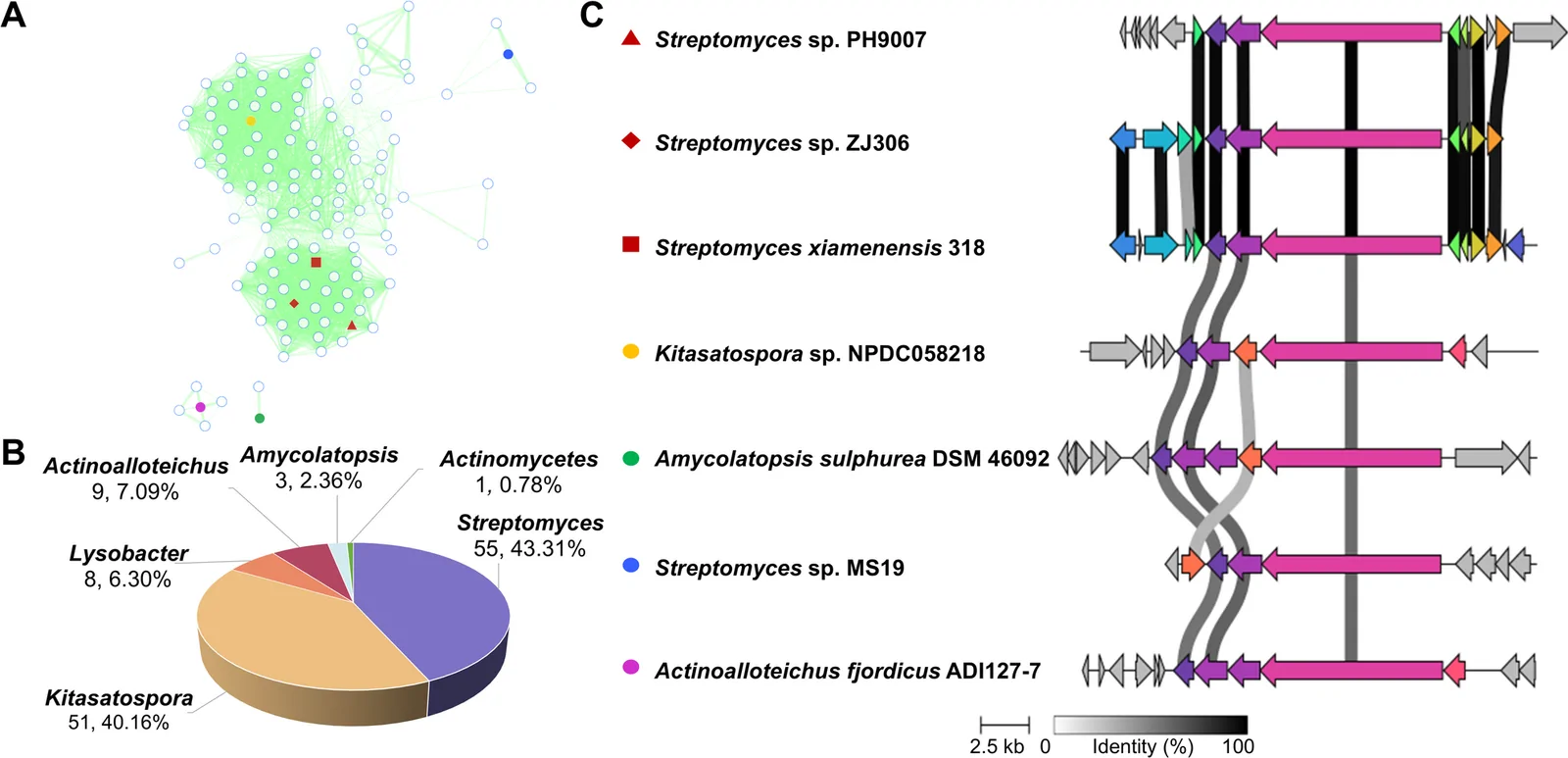
Distribution of the ikarugamycin-related PTM gene cluster across a wide range of bacteria. **A** Analysis of 127 ikarugamycin-related PTM gene clusters. **B** Representative ikarugamycin-related PTM gene clusters. **C** GenBank taxonomic distribution of the 127 identified ikarugamycin-related PTM producers

AntiSMASH analysis of the *Streptomyces* sp. PH9007 genome (GenBank accession: JBQRUY000000000) led to the prediction of a candidate BGC for the production of compounds 4–6, which is similar to the ikarugamycin (*ika*) BGC from *Streptomyces* sp. No. 8603. Three conserved enzymes, namely, the hybrid PKS/NRPS *ikaA*, the FAD-dependent oxidoreductase *ikaB*, and the alcohol dehydrogenase *ikaC*, have been confirmed to be responsible for constructing the intact scaffold of ikarugamycin. PH9007_*orf8* to PH9007_*orf10* are homologs of *ikaA* and *ikaC*, respectively (Table 2). To further investigate the presence of the ikarugamycin-related PTM BGC in bacteria, we used PH9007_*orf8* to PH9007_*orf10* as a query with the cblaster tool on the CAGECAT server. The search algorithm detected 127 clusters (Supporting Information II) containing homologs of PH9007_*orf8* to PH9007_*orf10* from 118 organisms encompassing five genera. The taxonomic distribution of these strains revealed that nearly 43.31% and 40.16% of them belonged to *Streptomyces* and *Kitasatospora*, respectively (Fig. 9B). Next, compounds 4–6 and the 127 ikarugamycin-related PTM gene clusters were analyzed by BIG-SCAPE, and a gene cluster similarity network was constructed using Cytoscape. These gene clusters were categorized into five groups (Fig. 9A), and representative gene clusters from each group were selected and aligned (Fig. 9C).

## Materials and methods

### Collection of the *S. acutum* sample

The medicinal plant *S*. acutum was collected from a mountain ditch in Zhongfang County, Huaihua City, Hunan Province, China (E110°3′5″, N27°28′17″). The plant was identified by Professor Weifeng Deng at the Department of Pharmacognosy, School of Pharmaceutical Sciences, Hunan University of Medicine. A voucher specimen (HY-PHSA-202110) has been deposited at the Pharmaceutical Experimental Center, Hunan University of Medicine.

### Isolation of actinomycetes

The isolation medium was supplemented with 50 mg/L of nystatin and 25 mg/L of nalidixic acid to inhibit fungal and bacterial growth. The roots and stems of *S. acutum* were pulverized in a mortar with a pestle on a sterile laboratory surface. The resulting mixture was homogenized by the addition of approximately 5 mL of sterile distilled water. The homogenate was subsequently aspirated, and the suspension was diluted 10^–1^, 10^–2^, and 10^–3^-fold. Finally, 0.2 mL of each suspension was added to four distinct selective isolation media. The samples were incubated at 28 °C for 7 days with periodic inversion. Afterward, the samples were kept at ambient temperature. During this period, daily inspections were performed, and specific colonies were selected on the basis of their growth and physical traits. The chosen colonies were subsequently purified at the same temperature on identical media. The purified strains were stored as glycerol suspensions in water (20%, *v*/*v*) at -80 °C.

### Genomic DNA extraction, 16S rRNA gene sequencing and phylogenetic tree construction

Two clones with notable morphological variations were selected and incubated in 20 mL of TSB medium. The mycelium biomass was collected using centrifugal precipitation after agitation at 200 rpm for five days at 30 °C. The genomic DNA was extracted from the harvested mycelium biomass using the EasyPure Bacteria Genomic DNA Kit (TransGen Biotech, Beijing, China) in accordance with the manufacturer’s instructions. The purity and concentration of the isolated DNA were assessed using agarose gel electrophoresis. The verified DNA was preserved at -20 °C for future use.

The 16S rRNA gene was amplified using the universal primer pair 27F/1492R [35], and the resulting PCR products were subsequently cloned and sequenced under the suggested conditions. The NCBI-BLASTn database was used to perform sequence similarity searches and determine pairwise similarity values. The partial 16S rRNA gene sequences of the two isolates were uploaded to the NCBI GenBank database and are accessible online. The MEGA 11 program was utilized to assess each sequence alignment [36].

### General methods

As previously reported, the instruments (including those used for MS and NMR) and standard reagents for chemical isolation and biological evaluation were utilized [37]; details are provided in the Supporting Information Ⅰ.

### *Streptomycetes* isolates

Strains PH9006 and PH9007 were identified as *Streptomyces* spp. on the basis of molecular identification, and their 16S rRNA sequences had 99% sequence identity to those of other *Streptomyces* species. Strains PH9006 and PH9007 were deposited at the School of Pharmaceutical Sciences, Hunan University of Medicine, Huaihua, China.

### Fermentation of *Streptomyces* sp. PH9007

The *Streptomyces* sp. PH9007 strain was grown on G1 agar plates and incubated at 30 °C to obtain spores. *Streptomyces* sp. PH9007 was subsequently grown in 250 mL Erlenmeyer flasks containing 50 mL of sterile seed medium (TSB) and incubated at 30 °C on a rotary shaker at 220 rpm for 48 h. Finally, 50 mL of the seed culture was transferred to 2 L Erlenmeyer flasks containing 500 mL of sterile fermentation medium (ISP4) containing 4% microporous resin D101 (Tianjin Kemiou Chemical Reagent Co., Ltd., Tianjin, China) and incubated at 30 °C for 7 days. After fermentation, the culture (95 L) was filtered to isolate the D101 resin, which was extracted with ethyl acetate and methanol by a supersonic wave. The ethyl acetate and methanol extracts were subsequently concentrated in vacuo to yield 76.2 g of oily crude extract.

### LC‒MS/MS and GNPS analyses

The crude extract was dissolved in MeOH and analyzed using a Q Exactive Focus Orbitrap mass spectrometer (Thermo Electron, Bremen, Germany) equipped with a heated electrospray ionization (HESI) source, which was connected to a Thermo Scientific Dionex Ultimate 3000 RS instrument (Thermo Fisher Scientific, California, USA). Chromatographic separation was performed on an Acquity UPLC BEH reversed-phase C18 column (100 mm × 2.1 mm, 1.7 µM). Elution was achieved with a gradient of 0.1% formic acid in H_2_O (A) and MeCN (B) at a flow rate of 0.3 mL/min as follows: H_2_O/MeCN, *v*/*v*, 95:5 → 85:15 (0–2 min); 85:15 → 65:35 (2–5 min); 65:35 → 40:60 (5–7 min); 40:60 → 5:95 (7–9 min); 5:95 → 95:5 (9–12 min); and 95:5 (12–15 min). MS/MS data acquisition was performed in positive electrospray ionization (ESI) mode. The resulting RAW files were subsequently transformed into open-source files .mzXML format employing ProteoWizard MSConvert (version 3) [38]. Files were transferred to the GNPS platform using WinSCP. Visualization of the MS² data was carried out utilizing the built-in tools within the GNPS. The molecular networks generated from the analysis were exported in GraphML format and further processed and visualized using Cytoscape software (version 3.9.1).

### Isolation of compounds 4–6

The MeOH/EtOAc-soluble fraction (76.2 g) was chromatographed on silica gel columns (200–300 mesh) with petroleum/EtOAc (*v*/*v*, 10:1 → 9:2 → 8:3 → 7:4 → 6:5 → 5:6 → 4:7 → 3:8 → 2:9 → 1:10 → 0:1) and EtOAc/MeOH (*v*/*v* 10:1 → 5:1 → 1:1 → 1:5 → 1:10) to yield six combined fractions (Fr.A to F). Fr. F (38.0 g) was separated on an ODS column (H_2_O/MeOH, *v*/*v* 8:2 → 7:3 → 6:4 → 5:5 → 4:6 → 3:7 → 2:8 → 1:9 → 0:1) to generate eight fractions (Fr. F-1 to Fr. F-8). Fr.F-3 (2.12 g) was subsequently run through an MCI gel highly porous polymer CHP20/P120 column (H_2_O/MeOH, *v*/*v* 9:1 → 8:2 → 7:3 → 6:4 → 5:5 → 4:6 → 3:7 → 2:8 → 1:9 → 0:1) to generate eight fractions (Fr.F-3-1 to Fr.F-3-8). The ODS was chosen to separate Fr.F-3-2 (0.165 g) from the mobile phase of H_2_O/MeOH (*v*/*v*, 95:5 → 90:10 → 85:15 → 80:20 → 75:25 → 70:30 → 60:40 → 50:50 → 40:60 → 30:70 → 20:80 → 10:90 → 0:100), and six fractions were separated (Fr.F-3-1 to Fr.F-3-6). Fr.F-3-2-2 (0.059 g) was purified using semipreparative HPLC with a gradient of MeCN/H_2_O [containing 0.03% formic acid, MeCN/H_2_O, *v*/*v*, 5:95 → 95:5 (0–10 min); 95:5 (10–12 min); 95:5 → 5:95 (12–12.5 min); 5:95 (12.5–20 min)] as the mobile phase to yield Capsimycin G (6, 7.36 mg, 10.5 min). Fr.F-7 (3.63 g) was separated on an MCI gel highly porous polymer CHP20/P120 column (H_2_O/MeOH, *v*/*v* 9:1 →8:2 → 7:3 → 6:4 → 5:5 → 4:6 → 3:7 → 2:8 → 1:9 → 0:1) to generate six fractions (Fr.F-7-1 to Fr.F-7-6). Fr.F-7-5 (0.744 g) was separated on an ODS column (H_2_O/MeOH, *v*/*v* 90:10 → 80:20 → 70:30 → 60:40 → 50:50 → 40:60 → 30:70 → 25:75 → 20:80 → 15:85 → 10:90 → 0:100) to generate four fractions (Fr.F-7-5-1 to Fr.F-7-5-4). Fr.F-7-5-3 (0.153 g) was separated on an ODS column (H_2_O/MeOH, *v*/*v* 90:10 → 80:20 → 70:30 → 60:40 → 50:50 → 40:60 → 35:65 → 30:70 → 25:75 → 20:80 → 15:85 → 10:90 → 0:100) to generate five fractions (Fr.F-7-5-3-1 to Fr.F-7-5-5-5). Fr. F-7-5-3-4 (0.039 g) and Fr. F-7-5-4 (2.12 g) were purified using semipreparative HPLC with a gradient of MeCN/H_2_O (containing 0.03% formic acid, MeCN/H_2_O, *v*/*v*, 40:60 → 85:15 (0–15 min), 85:15 (15–17 min), 85:15 → 10:90 (17–17.5 min), and 10:90 (17.5 → 20 min) as the mobile phase to yield Capsimycin (4, 6.78 mg, 13.5 min) and Capsimycin B (5, 13.78 mg, 18.8 min).

#### Capsimycin (4)

Colorless gum; LC-UV (ACN/H_2_O/0.3‰ FA) λmax 221.8, 325.1; ^1^H, ^13^C and 2D NMR spectroscopic data, see Figs. S4–S13; HRESIMS *m/z* 525.29572 [M + H]^+^ (calcd for C_30_H_42_N_2_O_6_^+^, 525.29591).

#### Capsimycin B (5)

Colorless gum; LC-UV (ACN/H_2_O/0.3‰ FA) λmax 221.8, 325.1; ^1^H, ^13^C and 2D NMR spectroscopic data, see Figs. S14–S23; HRESIMS *m/z* 495.28433 [M + H]^+^ (calcd for C_29_H_40_N_2_O_5_^+^, 495.28535).

#### Capsimycin G (6)

Colorless gum; LC-UV (ACN/H_2_O/0.3‰ FA) λmax 221.8, 325.1; ^1^H, ^13^C and 2D NMR spectroscopic data, see Figs. S24–S30; HRESIMS *m/z* 511.27966 [M + H]^+^ (calcd for C_29_H_40_N_2_O_6_^+^, 511.28026).

### α-Glucosidase inhibition assay

The α-glucosidase inhibitory activity was assessed using a slightly modified Worawalai method [39–41]. α-Glucosidase from *Saccharomyces cerevisiae* (Sigma-Aldrich, USA) was used for the enzymatic inhibition assay. Briefly, the compounds were incubated with α-glucosidase and 4-nitrophenyl α-D-glucopyranoside in phosphate buffer (pH 6.8) at 37 °C. The reaction was terminated with Na₂CO₃, and the absorbance was measured at 405 nm. Acarbose was used as a positive control. The Supplementary Information Ⅰ describes the reaction system.

### Fluorescence quenching assay

The fluorescence spectra of α-glucosidase with and without a quencher were measured using an F-7100 fluorescence spectrophotometer (Hitachi, Tokyo, Japan) at 298 K. α-Glucosidase was initially dissolved in PBS (0.1 M, pH 6.8) to prepare the enzyme solution (2 U/mL), while compound 5 was dissolved in DMSO to prepare the stock solution (50 µM). First, 2 µL of stock solution was added to the α-glucosidase solution (2 mL), followed by 2 µL of stock solution for each subsequent addition, for a total of 10 times. The emission spectra of the reaction system were recorded between 300 and 500 nm. The excitation wavelength was set to 280 nm, the slit width was set to 5 nm, the voltage was 400 V, and the scanning speed was 1200 nm/min.

### UV–visible spectra

The UV spectra were recorded using a UV-2600 spectrophotometer (Shimadzu, Tokyo, Japan). Different concentrations (from 2 to 20 µM) of 5 were added to an α-glucosidase (2 U/mL) solution, and absorption was recorded in the range of 190–500 nm after incubation for 5 min at 298 K. The corresponding concentration of 5 served as a control to correct the background.

### Molecular docking analysis

Molecular docking analyses were performed using AutoDock Vina 1.1.2. The crystal structure of the N-terminal catalytic subunit of human maltase-glucoamylase in complex with acarbose (PDB ID: 2QMJ) was retrieved from the Protein Data Bank and used as a structural model for investigating the potential binding interactions between the isolated compounds and an α-glucosidase-related target. The approach is outlined in the Supplementary Information Ⅰ.

### Molecular dynamic simulations

The approach is described in the Supplementary Information Ⅰ.

### Antibacterial assay

The MICs were determined using the broth dilution method [42]. The specific details are included in the Supplementary Information Ⅰ.

### Antifungal assay

The MICs were quantified through broth dilution [43, 44]. The specific details are included in the Supplementary Information Ⅰ.

## Conclusion

In this study, we identified a potential producer of PTMs from *S. acutum*, namely, *Streptomyces* sp. PH9007. Three known PTM derivatives, namely, capsimycin (4), capsimycin B (5) and capsimycin G (6), were isolated from *Streptomyces* sp. PH9007. Their structures were established by analysis of their NMR and HRESIMS spectra. These compounds were evaluated for their inhibitory activity against α-glucosidase, and 4 and 5 exhibited α-glucosidase inhibitory activity, with IC_50_ values of 484.6 ± 14.10 μM and 366.9 ± 12.85 μM, respectively. Fluorescence quenching, UV–visible spectroscopy, molecular docking and molecular dynamics simulations of 5 with α-glucosidase all indicated that this compound has a better binding effect with α-glucosidase. In addition, compound 5 exhibited antibacterial activity, with MICs of 16 µg/mL against both MRSA and *S. aureus* ATCC 29213. In addition, 5 exhibited antifungal activity, with MIC values of 16 µg/mL against *C. albicans* CMCC 98001 and 32 µg/mL against *C. parapsilosis* ATCC 22019. The whole genome of *Streptomyces* sp. PH9007 was subsequently sequenced, which revealed that it consists of a 6, 339, 217 bp linear chromosome and two linear plasmids of 83, 532 bp and 167, 087 bp. Twenty-five putative BGCs that are necessary for the biosynthesis of various natural products, including ikarugamycin, were identified in the genome of *Streptomyces* sp. PH9007 via genomic analysis. Bioinformatics analysis revealed that the ikarugamycin BGC is widely distributed in microorganisms. In view of the above results, these discoveries indicate that PTMs can be further investigated as α-glucosidase inhibitor promising compounds and establish a basis for the biosynthesis of this class of compounds.

## Supplementary Information

Supplementary Material 1.

Supplementary Material 2.

## Data Availability

Data availabilityThe sequencing data that provide evidence for the conclusions of the study are available in the NCBI GenBank database. The 16S rRNA gene sequences of strains PH9006 and PH9007 were deposited in the NCBI GenBank database under accession numbers PP624363 and PP625129. The genome sequence of strain PH9007 was deposited in the NCBI GenBank database under accession number JBQRUY000000000.
