# Supplementary material for: Capsimycin derivatives from Streptomyces sp. PH9007 as α‑glucosidase inhibitors and insights into their biosynthetic gene cluster
Source: BMC Genomics. 2026 Aug 18;27:944. doi: 10.1186/s12864-026-13269-7 (PMC13761111; doi:10.1186/s12864-026-13269-7)
Supplement: Supplementary file 1 [file 12864_2026_13269_MOESM1_ESM.pdf]

Supporting Information I

Capsimycin Derivatives from *Streptomyces* sp. PH9007 as α-Glucosidase Inhibitors and Insights into Their Biosynthetic Gene Cluster

Zhong Wang [1, a], Guangling Wu [1, a], Jian Pan [1, c], Runze Sun [a], Shun Cao [a], Xiaoyan Jin [d], Yani Zhong [a], Yuhan Chen [a], Lizi Cao [a], Xinlu Chen [a], Bin Feng [b, *], Xueshuang Huang [a, *], and Hong Pu [a, *]

[a]*Hunan Provincial Key Laboratory for Synthetic Biology of Traditional Chinese Medicine, School of Pharmaceutical Sciences, Hunan University of Medicine, Huaihua, Hunan, 418000, China;*

[b]*Huaihua Hospital of Traditional Chinese Medicine, Huaihua 418000, China;*

[c]*College of Pharmacy, Hunan Vocational College of Science and Technology, Changsha 410004, China;*

[d]*College of Pharmacy, Xinjiang Medical University, Urumchi, Xinjiang, 830011, China*

*__Corresponding author.__

E-mail address: puhong@hnmu.edu.cn (H. Pu), xueshuanghuang@126.com (X. S. Huang),

txz-1221@163.com (B. Feng)

[1] These authors contributed equally to this work

Table of Contents

Experimental Procedures ... 4

General experimental procedures ... 4

α-Glucosidase inhibition assay ... 4

Antibacterial assay ... 5

Antifungal assay ... 5

Molecular docking ... 6

Molecular dynamic simulations ... 6

MM/GBSA binding free energy calculation ... 7

Table S1 The media used in the experiment ... 8

Table S2 Comparison of ^{1}H- and ^{13}C-NMR data between Capsimycin **(**$CDCl_3$**)** and compound **4** in pyridine-d_5. .. 9

Table S3 Comparison of ^{1}H**-** and ^{13}C-NMR data between Capsimycin B **(**$CDCl_3$**)** and compound **5** in pyridine-d_5. ... 10

Table S4 Comparison of ^{1}H- and ^{13}C-NMR data between Capsimycin G (90% MeOD/ C_6D_6) and compound **6** in pyridine-d_5. ... 11

Table S5 Inhibition of antibacterial activity (MICs, μg/mL) of **4 ‒ 6.** ... 12

Table S6 Inhibition of antibacterial activity (MICs, μg/mL) of **4 ‒ 6.** ... 13

Table S7 Inhibition of α-glucosidase activity of **4 ‒ 6.** ... 14

Table S8 Logarithms of free binding energies (FBE, kcal/mol) of Capsimycin (**4**), Capsimycin B (**5**) and acarbose to the active cavities of α-glucosidase (PDB ID: 2QMJ) and targeting residues of the binding site located on the mobile flap. ... 15

Table S9 Binding free energies and energy components predicted by MM/GBSA (kcal/mol) ... 16

Table S10 Putative BGCs responsible for secondary metabolites of *Streptomyces*. sp. PH9007 ... 17

Figure S1 (**A**) HPLC analysis of crude extracts from *Streptomyces*. sp. PH9006 fermented in different media. (**B**) HPLC analysis of crude extracts from *Streptomyces*. sp. PH9007 fermented in different media. ... 18

Figure S2 16S RNA sequence data of *Streptomyces* sp. PH9006 ... 19

Figure S3 16S RNA sequence data of *Streptomyces* sp. PH9007 ... 20

Figure S4 ^{1}H NMR spectrum of **4** in pyridine-d_5 (600 MHz) ... 21

Figure S5 ^{13}C NMR spectrum of **4** in pyridine-d_5 (150 MHz) ... 22

Figure S6 DEPT-90 spectrum of **4** ... 23

Figure S7 DEPT-135 spectrum of **4** ... 24

Figure S8 HSQC spectrum of **4** ... 25

Figure S9 HMBC spectrum of **4** ... 26

Figure S10 ^{1}H-^{1}H COSY spectrum of **4** ... 27

Figure S11 NOESY spectrum of **4** ... 28

Figure S12 HRESIMS spectrum of **4** ... 29

Figure S13 UV spectrum of **4** ... 30

Figure S14 ^{1}H NMR spectrum of **5** in pyridine-d_5 (400 MHz) ... 31

Figure S15 ^{13}C NMR spectrum of **5** in pyridine-d_5 (100 MHz) ... 32

Figure S16 DEPT-90 spectrum of **5** ... 33

Figure S17 DEPT-135 spectrum of **5** ... 34

Figure S18 HSQC spectrum of **5** ... 35

Figure S19 HMBC spectrum of **5** ... 36

Figure S20 ^{1}H-^{1}H COSY spectrum of **5** ... 37

Figure S21 NOESY spectrum of **5** 38
Figure S22 HRESIMS spectrum of **5** 39
Figure S23 UV spectrum of **5** 40
Figure S24 ^{1}H NMR spectrum of **6** in pyridine-d_5 (600 MHz) 41
Figure S25 ^{13}C NMR spectrum of **6** in pyridine-d_5 (150 MHz) 42
Figure S26 DEPT-135 spectrum of **6** 43
Figure S27 HSQC spectrum of **6** 44
Figure S28 HMBC spectrum of **6** 45
Figure S29 HRESIMS spectrum of **6** 46
Figure S30 UV spectrum of **6** 47
Figure S31 Inhibition rates of the combination of compounds **4** (A), **5** (B) and acarbose (C) against α-glucosidase in vitro at gradient concentrations 48
Figure S32 Docking poses and interactions of compound **4** and acarbose with α-glucosidase (PDB ID: 2QMJ). 49
Figure S33 96 well plate assay of **4**－**6** against MRSA (A), *S. aureus* ATCC 29213 (B), *Klebsiella pneumonia* ATCC 113 (C) and *Pseudomonas aeruginosa* ATCC 9027 (D) using the microbroth dilution method. 50
References 51

Experimental Procedures

General experimental procedures

Ultraviolet spectra were acquired using a Waters 2998 PDA Detector [Waters Technology (Shanghai) Co., Ltd., Shanghai, China]. Semi-preparative reversed-phase high-performance liquid chromatography (RP-HPLC) was conducted utilizing a Waters 1525 Binary HPLC pump, paired with a Waters 2489 UV/visible detector [Waters Technology (Shanghai) Co., Ltd., Shanghai, China], and employing a Welch Ultimate AQ-C18 column (250 × 10 mm, 5 μm) [Welch Technology (Shanghai) Co., Ltd., Shanghai, China]. HRESIMS spectra were obtained using a Q-Exactive Focus Orbitrap MS (Thermo Electron, Bremen, Germany) linked to the Thermo Scientific Dionex Ultimate 3000 RS (Thermo Fisher Scientific, California, USA). NMR spectra were obtained with a Bruker 400 MHz or 600 MHz spectrometer (Bruker Corporation, Massachusetts, USA). The chemical shifts in ^{1}H NMR and ^{13}C NMR spectra were calibrated against the solvents for pyridine-d_5 (δ_H 7.58 and δ_C 135.7) (Cambridge Isotope Laboratories, Inc., Massachusetts, USA). Column chromatography (CC) was performed using silica gel (200–300 mesh, Yantai Jiangyou Silica Gel Development Co., Ltd., Yantai, China), RP-C18 (AAG12S50, YMC Co., Ltd., Kyoto, Japan), and MCI GEL CHP20/P120 (Mitsubishi Chemical Group Corporation, Tokyo, Japan).

α-Glucosidase inhibition assay

The inhibitory activity of compounds **4**–**6** against α-glucosidase [Sigma-Aldrich (Shanghai) Trading Co., Ltd., Shanghai, China, Product No. G5003] was examined utilizing a modified version of the Worawalai method [1]. The chemicals were solubilized in DMSO (Shanghai Macklin Biochemical Technology Co., Ltd., Shanghai, China). The α-glucosidase enzyme and 4-Nitrophenyl α-D-glucopyranoside (PNPG) (Shanghai Aladdin Biochemical Technology Co., Ltd., Shanghai, China) were each diluted in a 0.1 M potassium phosphate buffer (Shanghai Aladdin Biochemical Technology Co., Ltd., Shanghai, China) at pH 6.8. Four groups were established: the test group containing the test compound and the enzyme, the control group with solely the enzyme, the sample group with only the test compound, and the blank group devoid of any components. Each group was supplemented with potassium phosphate buffer and PNPG to create a 200 μL reaction system. In the experimental group, 10 μL of α-glucosidase (final concentration 0.1 U/mL), 130 μL of phosphate buffer, and 10 μL of the test compounds were sequentially put to 96-well plates (Corning Incorporated, New York, USA). Following incubation at 37 ℃ for 20 minutes, 50 μL of PNPG (final concentration 1 mmol/L) was introduced into the mixture, which was then incubated at 37 ℃ for an additional 40 minutes. Ultimately, 100 μL of Na_2CO_3 (0.5 M) (Shanghai Aladdin Biochemical Technology Co., Ltd., Shanghai, China) was included into the mixture to terminate the reaction. The enzymatic activity was evaluated by measuring absorbance at 405 nm with a multi-

mode microplate reader (BMG Labtech, Ortenberg, Germany). The percentage of inhibition was computed as follows: Percentage of inhibition = [1 - (A_s - A_t) / (A_c - A_b)] × 100%, where A_s represents the absorbance of the sample group, A_t denotes the absorbance of the test group, A_c indicates the absorbance of the control group, and A_b signifies the absorbance of the blank group.

Antibacterial assay

The antibacterial activity of compounds **4–6** was evaluated against *Klebsiella pneumoniae*, *Pseudomonas aeruginosa*, methicillin-resistant *Staphylococcus aureus* (MRSA), and *S. aureus* ATCC 29213. The method used to determine the MICs was the broth dilution test. The bacterial strains were cultivated overnight, and the resulting culture was then diluted to a concentration of 10^6 CFU/mL in Luria-Bertani (LB) broth. This broth was made up of tryptone (Thermo Scientific Oxoid, Massachusetts, USA), yeast extract (Thermo Scientific Oxoid, Massachusetts, USA), and NaCl (Sinopharm Chemical Reagent Co., Ltd., Shanghai, China). In order to prepare the 96-well plate, a serial dilution was performed in LB broth using ten different doses (64, 32, 16, 8, 4, 2, 1, 0.5. 0.25, 0.125 μg/mL). The compounds **4–6** were dissolved in DMSO before undergoing the serial dilution process. Levofloxacin, which was manufactured by Bide Pharmatech Ltd. of Shanghai, China, was dissolved in dimethyl sulfoxide (DMSO), and the resulting solution was serially diluted to create ten different concentrations ranging from 0.125 to 64 micrograms per milliliter. This process was carried out using LB broth on each of the 96-well plates. The next step was to combine a solution of bacteria with 100 μL of LB broth containing either compounds **4–6** or levofloxacin in each well. For a period of 18 hours, the plates were incubated at a temperature of 37 ℃. In order to better visualize the outcome, each well was then filled with fifty microliters of resazurin (Shanghai Macklin Biochemical Technology Co., Ltd., Shanghai, China). Each of the 96-well plates was used to test both **4–6** and levofloxacin in triplicate.

Antifungal assay

Antifungal assay was based on broth dilution assay CLSI-M27-A3 by the clinical and laboratory standard institute (CLSI). Compounds **4–6** were tested for antifungal activity against *Candida albicans* CMCC 98001 and *Candida parapsilosis* ATCC 22019. The MICs were determined using broth dilution assay. The fungal strains were cultured overnight and diluted to 2 × 10^3 cells/mL in RPMI-1640 medium buffered with MOPS (pH 7.0) **4–6** were dissolved in DMSO, serially diluted to 10 different concentrations (64, 32, 16, 8, 4, 2, 1, 0.5. 0.25, 0.125 μg/mL) using RPMI-1640 broth on the 96 well plate. Fluconazole (Bide Pharmatech Ltd., Shanghai, China) was dissolved in ultrapure water, serially diluted to 10 concentrations (0.125–64 μg/mL) using RPMI-1640 broth on each 96 well plate. Then 100 μL RPMI-1640 broth containing compounds **4–6** or fluconazole and 100 μL of

fungal solution were mixed per well. The plates were incubated at 37 °C for 24 h. Finally, the drug effect was assessed visually. Wells exhibiting a clear solution without fungal growth were considered positive. The minimum inhibitory concentration (MIC) was defined as the lowest drug concentration showing no fungal growth. The experiment was performed in at least triplicate.

Molecular docking

Autodock Vina 1.1.2 [2] was used to conduct molecular docking investigations in order to examine the binding mechanism between the N-terminal subunit of human maltase-glucoamylase in complex with acarbose (PDB ID 2QMJ) and the derivative capsimycin (**4**) as well as capsimycin B (**5**). The docking input papers were created using the AutoDockTools 1.5.6 package[3, 4]. The search grid of a-glucosidase was characterized by the following dimensions: size_x: 15, size_y: 15, and size_z: 16.5. Its center was located at the coordinates center_x: -21.727, center_y: -6.323, and center_z: -5.281. The default parameters in Vina docking were adopted for all calculations unless otherwise specified. The Vina docking score was used to select the best scoring pose, and PyMOL 1.8 software (available at https://www.pymol.org) was used to do visual analysis.

Molecular dynamic simulations

The AMBER 18 software package was utilized to conduct molecular dynamics simulations for simulation [5]. The system was made as energy efficient as possible before to the simulation. This involved using the steepest descent approach, which had 2500 stages, as well as the conjugate gradient method, which also had 2500 steps. The system's temperature was gradually raised from 0 K to 298.15 K by heating it for 200 picoseconds at a constant heating rate while maintaining a constant volume; this was done after the system had undergone energy optimization. For a duration of 500 ps, the NVT (isothermal isobody) system simulation was conducted in order to ensure that the solvent molecules were dispersed more equally across the solvent box. The simulation was carried out under the condition that the system maintenance temperature was maintained at 298.15 K. In the instance of NPT, which is an acronym for isothermal and isopressure, the equilibrium simulation of the entire system was carried out for a period of 500 picoseconds before finally reaching its conclusion. In conclusion, the NPT (isothermal and isopressure) system simulation was performed for both of the composite systems under periodic boundary conditions over a period of 100 nanoseconds. The nonbonded cutoff distance was established at 10 Å for simulations, and the Particle Mesh Ewald (PME) method was employed to calculate electrostatic interactions that occurred over long distances [6]. The SHAKE method was also utilized in order to limit the length of hydrogen atomic bonds [7], and the Langevin algorithm was utilized in order to control the temperature [8]. The collision frequency, denoted by the symbol γ, was set at 2 ps-1. For the purposes of later analysis, trajectories were

kept at intervals of ten picoseconds. Additionally, the system pressure was one atmosphere, and the integration step was two femtoseconds.

MM/GBSA binding free energy calculation

The MM/GBSA technique [9–12] was used to compute the binding free energies between protein and ligand for all systems. It is possible that the accuracy of MM/GBSA calculations [9],will be compromised if the molecular dynamics simulation is run for a long period of time. Because of this, the MD trajectory of 45–50 ns is utilized for the calculations in this investigation, and the precise formula is as follows:

$$\begin{aligned}\Delta G_{bind} &= \Delta G_{\mathrm{complex}} - (\Delta G_{\mathrm{receptor}} + \Delta G_{\mathrm{ligand}}) \\ &= \Delta E_{\mathrm{internal}} + \Delta E_{\mathrm{VDW}} + \Delta E_{\mathrm{elec}} + \Delta G_{\mathrm{GB}} + \Delta G_{\mathrm{SA}} \qquad (1)\end{aligned}$$

The symbols that are used in equation (1) are as follows: indicates the electrostatic interaction, denotes the van der Waals action, and denotes the internal energy. Bond energy (Ebond), angle energy (Eangle), and torsion energy (Etorsion) are all components of the internal energy of a system. They are collectively referred to as solvation energies that are free from any outside factors. The polar solvation free energy is represented by GGB, while the non-polar solvation free energy is represented by GSA in this case. To do calculations, this work used the GB model that was created by Nguyen et al. [13], with igb = 2. The non-polar solvation free energy (GSA) was determined by multiplying the surface tension (γ) by the solvent accessible surface area (SA), which resulted in the equation GSA= 0.0072 × SASA [14]. Because of the significant consumption of computing resources and the low level of accuracy [9, 10], the entropy change is not taken into account in this work.

Table S1 The media used in the experiment.

Media	Compositions
G1	2% soluble starch, 5×10^{-2}% NaCl, 0.1% KNO_3, 5×10^{-2}% $K_2HPO_4\cdot3H_2O$, 5×10^{-2}% $MgSO_4\cdot7H_2O$, 1×10^{-3}% $FeSO_4\cdot7H_2O$, 2% agar powder
ISP4	1% soluble starch, 0.1% $K_2HPO_4\cdot3H_2O$, 0.1% $MgSO_4\cdot7H_2O$, 0.1% NaCl, 0.2% $(NH_4)_2SO_4$, 0.2% $CaCO_3$, 1×10^{-4}% $FeSO_4\cdot7H_2O$, 1×10^{-4}% $MnCl_2\cdot4H_2O$, 2% agar powder
MH13	2% soluble starch, 0.5% sucrose, 2% fishmeal, 1×10-2% CuSO4·5H2O, 5×10-4% NaI, 0.2% CaCO3
MH18	2% soluble starch, 2% corn flour, 5×10^{-2}% KH_2PO_4, 2.5×10^{-2}% $MgSO_4$, 0.1% $ZnSO_4\cdot7H_2O$, 0.1% $FeSO_4\cdot7H_2O$, 0.1% $MnCl_2\cdot4H_2O$, 0.1% $CaCl_2$, 0.5% $CaCO_3$
MH20	3% soluble starch, 1% soybean flour, 0.5% yeast extract, 0.3% $CaCO_3$
MH25	2% soluble starch, 1% glucose, 0.5% soya peptone, 0.5% yeast extract, 0.4% NaCl, 5×10^{-2}% K_2HPO_4, 5×10^{-2}% $MgSO_4\cdot7H_2O$, 0.5% $CaCO_3$

Table S2 Comparison of ^{1}H- and ^{13}C-NMR data between Capsimycin **(**$CDCl_3$**)** and compound **4** in pyridine-d_5.

No	Capsimycin		4	
	**δ_C, type**	**δ_H (*J* in Hz)**	**δ_C, type**	**δ_H (*J* in Hz)**
1	196.5, C		197.50, C	
2	61.6, CH	3.83, br d (3.6)	62.55, CH	4.10, m
3	27.2, CH_2	1.75, m 1.97, m	28.39, CH_2	2.18, m, 2.04, m
4	20.9, CH_2	1.19, m 1.47, m	22.19, CH_2	1.74, m, 1.54, m
5	38.9, CH_2	2.55, br t (10.4) 3.61, m	39.91, CH_2	3.86, m, 2.77, m
7	166.3, C		167.48, C	
8	124.2, CH	5.82, dd (11.4, 2.0)	125.97, CH	6.25, m
9	140.0, CH	6.04, ddd (11.4, 11.4, 2.3)	139.89, CH	5.98, m
10	25.3, CH_2	2.36, m 3.67, m	26.68, CH_2	4.20, m, 2.63, m
11	45.5, CH	1.65, m	46.74, CH	1.55, m
12	40.1, CH	2.31, m	41.33, CH	2.41, m
13	53.8, CH	2.87, d (4.0)	54.35, CH	2.95, m
14	58.3, CH	3.22, dd (4.0, 2.1)	58.73, CH	3.27, m
15	47.1, CH	0.95, m	48.05, CH	1.06, m
16	50.1, CH	1.87, m	50.89, CH	2.02, m
17	33.6, CH	2.30, m	34.64, CH	2.38, m
18	39.0, CH_2	0.59, m 1.96, m	39.82, CH_2	1.90, m, 0.56, m
19	47.5, CH	1.07, m	48.12, CH	1.06, m
20	40.8, CH	1.67, m	41.78, CH	1.66, m
21	36.8, CH_2	1.11, m 2.07, m	37.70, CH_2	1.95, m 1.00, m
22	48.7, CH	2.44, m	49.48, CH	2.33, m
23	151.2, CH	6.69, dd (15.4, 10.1)	149.37, CH	6.90, m
24	122.7, CH	7.11, d (15.4)	125.58, CH	7.76, m
25	173.6, C		175.18, C	
26	100.7, C		102.80, C	
27	175.7, C		177.67, C	
29	17.7, CH_3	0.93, d (7.2)	18.58, CH_3	1.03, d
30	77.4, CH	3.30, m	78.12, CH	3.28, m
31	17.4, CH_3	1.25, d (7.2)	18.15, CH_3	1.33, d
32	55.2, CH_3O	3.25, s	55.62, CH_3	3.26, s

Table S3 Comparison of ^{1}H- and ^{13}C-NMR data between Capsimycin B **(**$CDCl_3$**)** and compound **5** in pyridine-d_5.

**No**	**Capsimycin B**		**5**	
	δ_C, **type**	δ_H **(*J* in Hz)**	δ_C, **type**	δ_H **(*J* in Hz)**
1	195.9, C		197.35, C	
2	61.4, CH	3.85, br d (3.7)	62.76, CH	4.12, d, (3.7)
3	27.4, CH_2	1.77, m, 1.98, m	28.37, CH_2	2.14, m, 2.03, m
4	21.0, CH_2	1.20, m, 1.51, m	22.09, CH_2	1.74, m, 1.51, m
5	38.9, CH_2	2.56, br t (11.4) 3.63, m	39.83, CH_2	3.87, m, 2.77, m
7	166.3, C		167.35, C	
8	124.2, CH	5.79, dd (11.6, 2.0)	125.88, CH	6.24, dd, (11.3, 2.4)
9	140.4, CH	6.06, ddd (11.6, 11.6, 2.5)	139.97, CH	5.99, td, (11.1, 3.3)
10	25.5, CH_2	2.38, m, 3.61, m	26.64, CH_2	4.24, m, 2.63, m
11	45.2, CH	1.65, m	46.43, CH	1.58, m
12	40.8, CH	2.34, m	41.67, CH	2.42, m
13	53.5, CH	2.89, d (3.8)	53.92, CH	2.98, m
14	57.7, CH	3.13, dd (3.8, 2.0)	58.04, CH	3.17, m
15	49.6, CH	0.91, m	50.77, CH	0.97, m
16	46.6, CH	1.60, m	47.01, CH	1.60, m
17	33.0, CH	2.18, m	33.89, CH	2.16, m
18	38.3, CH_2	0.59, m, 1.96, m	39.19, CH_2	1.93, m, 0.53, m
19	47.9, CH	1.01, m	48.29, CH	0.94, m
20	40.7, CH	1.70, m	41.52, CH	1.67, m
21	36.6, CH_2	1.11, m, 2.06, m	37.40, CH_2	1.94, m, 1.02, m
22	48.9, CH	2.46, m	49.74, CH	2.33, m
23	151.6, CH	6.70, dd (15.6, 10.2)	150.35, CH	6.94 dd, (15.5, 10.2)
24	122.7, CH	7.11, d (15.6)	124.60, CH	7.68, d, (15.5)
25	173.7, C		174.03, C	
26	100.6, C		102.58, C	
27	175.5, C		177.39, C	
29	17.5, CH_3	0.81, d (7.3)	17.98, CH_3	0.83, d, (7.1)
30	22.5, CH_2	1.44, m	23.58, CH_2	1.41, m
31	13.2, CH_3	0.94, d (7.3)	13.83, CH_3	0.99, t, (7.4)

Table S4 Comparison of ^{1}H- and ^{13}C-NMR data between Capsimycin G (90% MeOD/ C_6D_6) and compound **6** in pyridine-d_5.

No	Capsimycin G		6	
	**δ_C, type**	**δ_H (J in Hz)**	**δ_C, type**	**δ_H (J in Hz)**
1	198.0, C		197.62, C	
2	62.5, CH	3.78, br d (3.8)	62.75, CH	4.13, m
3	27.9, CH_2	1.85, m 2.00, m	28.43, CH_2	2.19, m, 2.09, m
4	21.7, CH_2	1.26, m 1.52, m	22.21, CH_2	1.76, m, 1.58, m
5	39.7, CH_2	2.64, m 3.46, br d (12.0)	39.91, CH_2	3.88, m, 2.78, m
7	168.6, C		167.59, C	
8	124.9, CH	5.91, d (11.9)	125.98, CH	6.28, d, (11.4)
9	141.4, CH	6.05, ddd (11.9, 10.1, 2.8)	140.10, CH	6.00, t, (10.6)
10	26.8, CH_2	2.42, m 3.63, m	26.76, CH_2	4.22, m, 2.67, m
11	47.1, CH	1.61, m	46.86, CH	1.61, m
12	41.5, CH	2.28, m	41.44, CH	2.43, m
13	55.1, CH	2.89, d (3.8)	54.50, CH	2.98, m
14	59.4, CH	3.26, br s	58.98, CH	3.40, m
15	48.3, CH	0.93, m	48.07, CH	1.17, m
16	52.8, CH	1.88, m	53.15, CH	2.18, m
17	34.8, CH	2.39, m	34.78, CH	2.60, m
18	40.0, CH_2	0.65, td (11.9, 8.1) 2.04, m	39.91, CH_2	2.00, m, 0.65, m
19	48.6, CH	1.12, m	48.51, CH	1.05, m
20	42.5, CH	1.68, m	42.07, CH	1.73, m
21	38.0, CH_2	1.10, m 2.02, m	37.80, CH_2	1.99, m, 1.05, m
22	50.0, CH	2.35, m	49.65, CH	2.34, m
23	149.3, CH	6.68, dd (15.0, 9.8)	150.35, CH	6.94, dd, (15.4, 10.2)
24	124.7, CH	7.28, m	125.17, CH	7.72, d, (15.5)
25	176.3, C		174.76, C	
26	102.4, C		102.76, C	
27	177.5, C		177.55, C	
29	18.3, CH_3	1.08, d (7.7)	19.21, CH_3	1.32, d, (7.0)
30	68.9, CH	3.87, m	68.37, CH	4.15, m
31	23.5, CH_3	1.36, d (6.3)	24.78, CH_3	1.64, d, (6.0)

Table S5 Inhibition of antibacterial activity (MICs, μg/mL) of **4–6.**

Compounds	Pathogenic bacterium (MIC μg/mL)			
	S. aureus ATCC 29213	MRSA	*K. pneumonia* ATCC 113	*P. aeruginosa* ATCC 9027
**4**	>64	>64	>64	>64
**5**	16	16	>64	>64
**6**	>64	>64	>64	>64

Table S6 Inhibition of antibacterial activity (MICs, μg/mL) of **4–6.**

Compounds	Pathogenic fungi (MIC, μg/mL)	
	C. parapsilosis ATCC 22019	*C. albicans* CMCC 98001
**4**	＞64	＞64
**5**	32	16
**6**	＞64	＞64
Fluconazole	4	4

Table S7 Inhibition of α-glucosidase activity of **4–6.**

Compounds	Concentration (μM)	Inhibition rate (%, mean ± SD, n=3)	IC_{50} (μM, mean ± SD)
	75	0.62 ± 2.38	
	150	6.73 ± 3.58	
**4**	300	24.40 ± 5.45	484.6 ± 14.10
	400	41.82 ± 5.80	
	600	60.20 ± 3.37	
	75	7.20 ± 1.91	
	150	20.90 ± 2.40	
**5**	300	42.31 ± 1.47	366.9 ± 12.85
	400	48.66 ± 2.30	
	600	73.22 ± 3.69	
**6**		NA[a]	
	50	-0.28 ± 4.09	
	100	7.55 ± 3.86	
Acarbose	200	18.93 ± 1.59	671.5 ± 0.17
	400	35.21 ± 2.70	
	800	55.79 ± 1.15	

[a] NA: not active.

Table S8 Logarithms of free binding energies (FBE, kcal/mol) of Capsimycin (**4**), Capsimycin B (**5**) and acarbose to the active cavities of α-glucosidase (PDB ID: 2QMJ) and targeting residues of the binding site located on the mobile flap.

compounds	-log (FBE)	targeting residues
Capsimycin	-8.2	Arg-202, Asp-203, Thr-204, Thr-205, Tyr-299, Trp-406, Leu-473, Phe-575
Capsimycin B	-7.6	Arg-202, Asp-203, Thr-204, Tyr-299, Trp-406, Phe-575
Acarbose	-6.7	Arg-526, Asp-542, Asp-327, Trp-406, Tyr-299, Tyr-605

Table S9 Binding free energies and energy components predicted by MM/GBSA (kcal/mol)

System	α-glucosidase/Acarbose	α-glucosidase/Capsimycin_B
ΔE_{vdW}	-16.70 ± 3.34	-35.46 ± 2.42
ΔE_{elec}	-44.74 ± 6.44	-15.47 ± 6.82
ΔG_{GB}	46.91 ± 6.28	35.78 ± 8.20
ΔG_{SA}	-2.24 ± 0.51	-6.92 ± 0.36
ΔG_{bind}	-16.77 ± 1.25	-22.07 ± 3.89

ΔE_{vdW}: van der Waals energy.

ΔE_{elec}: electrostatic energy.

ΔG_{GB}: electrostatic contribution to solvation.

ΔG_{SA}: non-polar contribution to solvation.

ΔG_{bind}: binding free energy.

Table S10 Putative BGCs responsible for secondary metabolites of *Streptomyces*. sp. PH9007

Cluster	Type	Most similar known cluster	Similarity	Cluster	Type	Most similar known cluster	Similarity
Cluster 1	T1PKS, NRPS-like, T3PKS	kendomycin	40%	Cluster 14	NRPS, T1PKS, linaridin	ikarugamycin	56%
Cluster 2	lassopeptide	pyrrolomycin A/B/C/D	5%	Cluster 15	terpene	—	—
Cluster 3	NRPS,linaridin	cypemycin	77%	Cluster 16	thiopeptide,LAP	conglobatin	10%
Cluster 4	NI-siderophore	legonoxamine A/B/desferrioxamine B	50%	Cluster 17	NI-siderophore	kinamycin	13%
Cluster 5	ectoine	ectoine	100%	Cluster 18	hydrogen-cyanide,NRPS	valinomycin/montanastatin	17%
Cluster 6	nucleoside	—	—	Cluster 19	NRPS, T1PKS	carotenoid	18%
Cluster 7	terpene	chlortetracycline	5%	Cluster 20	terpene	hopene	30%
Cluster 8	RRE-containing	colibrimycin	3%	Cluster 21	T3PKS	naringenin	100%
Cluster 9	lanthipeptide-class-i	—	—	Cluster 22	NAPAA,terpene,NRPS	deoxyhangtaimycin	22%
Cluster 10	NRPS, T1PKS	depsibosamycin B/C/D	14%	Cluster 23	T1PKS, NRPS	lividomycin	6%
Cluster 11	other	colibrimycin	11%	Cluster 24	T1PKS	butyrolactol A	26%
Cluster 12	butyrolactone	methylenomycin A	9%	Cluster 25	NRPS	triacsin C	25%
Cluster 13	lanthipeptide-class-i,linaridin	naphthomycin A	6%				

Secondary metabolite types detected by antiSMASH: T1PKS, type I PKS cluster; T3PKS, type III PKS cluster; NRPS, nonribosomal peptide synthetase cluster; other cluster containing a secondary metabolite, related protein that did not fit into any other category. The similarity is the percentage of homologous genes in the query cluster that are present in the hit cluster of the antiSMASH database. —, not available.

Figure S1 (**A**) HPLC analysis of crude extracts from *Streptomyces*. sp. PH9006 fermented in different media. (**B**) HPLC analysis of crude extracts from *Streptomyces*. sp. PH9007 fermented in different media.

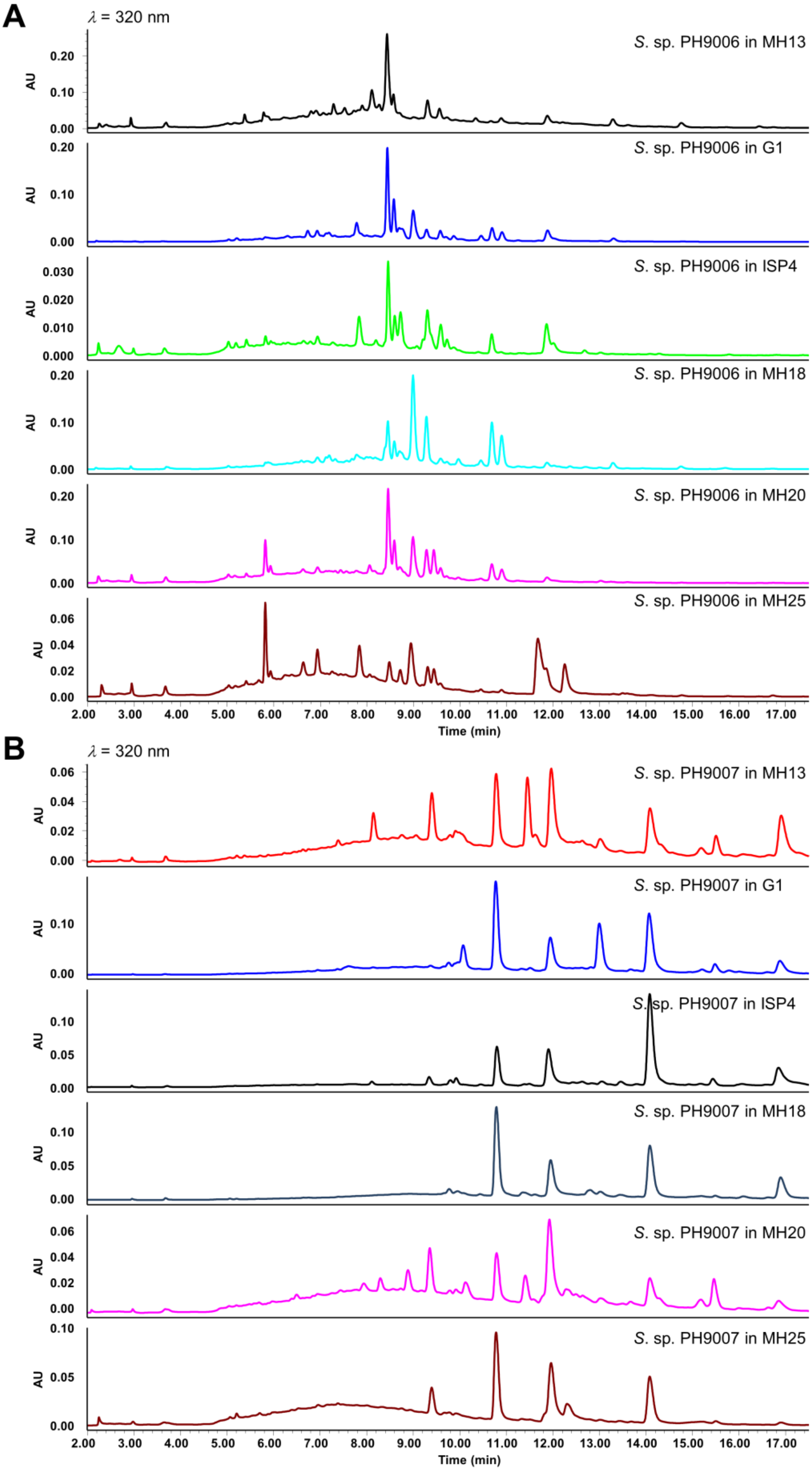

Figure S2 16S rRNA sequence data of *Streptomyces* sp. PH9006

CGGGGGGGGTGCTTACCATGCAAGTCGAACGATGAAGCCCTTCGGGGTGGATTAGTGGCGAACGGGT
GAGTAACACGTGGGCAATCTGCCCTTCACTCTGGGACAAGCCCTGGAAACGGGGTCTAATACCGGAT
ACCACTCCTGCCTGCATGGGCGGGGGTTGAAAGCTCCGGCGGTGAAGGATGAGCCCGCGGCCTATCA
GCTTGTTGGTGGGGTAATGGCCTACCAAGGCGACGACGGGTAGCCGGCCTGAGAGGGCGACCGGCC
ACACTGGGACTGAGACACGGCCCAGACTCCTACGGGAGGCAGCAGTGGGGAATATTGCACAATGGG
CGAAAGCCTGATGCAGCGACGCCGCGTGAGGGATGACGGCCTTCGGGTTGTAAACCTCTTTCAGCAG
GGAAGAAGCGAAAGTGACGGTACCTGCAGAAGAAGCGCCGGCTAACTACGTGCCAGCAGCCGCGGT
AATACGTAGGGCGCAAGCGTTGTCCGGAATTATTGGGCGTAAAGAGCTCGTAGGCGGCTTGTCACGTC
GGATGTGAAAGCCCGAGGCTTAACCTCGGGTCTGCATTCGATACGGGCTAGCTAGAGTGTGGTAGGG
GAGATCGGAATTCCTGGTGTAGCGGTGAAATGCGCAGATATCAGGAGGAACACCGGTGGCGAAGGCG
GATCTCTGGGCCATTACTGACGCTGAGGAGCGAAAGCGTGGGGAGCGAACAGGATTAGATACCCTGG
TAGTCCACGCCGTAAACGTTGGGAACTAGGTGTTGGCGACATTCCACGTCGTCGGTGCCGCAGCTAA
CGCATTAAGTTCCCCGCCTGGGGAGTACGGCCGCAAGGCTAAAACTCAAAGGAATTGACGGGGGCCC
GCACAAGCGGCGGAGCATGTGGCTTAATTCGACGCAACGCGAAGAACCTTACCAAGGCTTGACATAT
ACCGGAAAGCATTAGAGATAGTGCCCCCCTTGTGGTCGGTATACAGGTGGTGCATGGCTGTCGTCAGC
TCGTGTCGTGAGATGTTGGGTTAAGTCCCGCAACGAGCGCAACCCTTGTCCTGTGTTGCCAGCATGCC
CTTCGGGGTGATGGGGACTCACAGGAGACCGCCGGGGTCAACTCGGAGGAAGGTGGGGACGACGTC
AAGTCATCATGCCCCTTATGTCTTGGGCTGCACACGTGCTACAATGGCCGGTACAATGAGCTGCGATA
CCGTGAGGTGGAGCGAATCTCAAAAAGCCGGTCTCAGTTCGGATTGGGGTCTGCAACTCGACCCCAT
GAAGTCGGAGTCGCTAGTAATCGCAGATCAGCATTGCTGCGGTGAATACGTTCCCGGGCCTTGTACAC
ACCGCCCGTCACGTCACGAAAGTCGGTAACACCCGAAGCCGGTGGCCCAACCCGTAAGGAGGGAG
CTGTCGAAGGTGGGACTGGCGATTGGGACGAAGTCGTAACAAGGTAGCCGTACCGGAAGTGGTGGG
GGTTCCCCCCCTCTATAAGGGGG

Figure S3 16S rRNA sequence data of *Streptomyces* sp. PH9007

CGGGCGGGGTGCTTACCGTGCAAGTCGAACGATGAAGCCCTTCGGGGTGGATTAGTGGCGAACGGGT
GAGTAACACGTGGGCAATCTGCCCTTCACTCTGGGACAAGCCCTGGAAACGGGGTCTAATACCGGAT
AACACTTCCACTCTCCTGAGTGGAGGTTAAAAGCTCCGGCGGTGAAGGATGAGCCCGCGGCCTATCA
GCTTGTTGGTGAGGTAATGGCTCACCAAGGCGACGACGGGTAGCCGGCCTGAGAGGGCGACCGGCC
ACACTGGGACTGAGACACGGCCCAGACTCCTACGGGAGGCAGCAGTGGGGAATATTGCACAATGGG
CGAAAGCCTGATGCAGCGACGCCGCGTGAGGGATGACGGCCTTCGGGTTGTAAACCTCTTTCAGCAG
GGAAGAAGCGAAAGTGACGGTACCTGCAGAAGAAGCGCCGGCTAACTACGTGCCAGCAGCCGCGGT
AATACGTAGGGCGCGAGCGTTGTCCGGAATTATTGGGCGTAAAGAGCTCGTAGGCGGCTTGTCACGTC
GGTTGTGAAAGCCCGGGGCTTAACCCCGGGTCTGCAGTCGATACGGGCAGGCTAGAGTTCGGTAGGG
GAGATCGGAATTCCTGGTGTAGCGGTGAAATGCGCAGATATCAGGAGGAACACCGGTGGCGAAGGCG
GATCTCTGGGCCGATACTGACGCTGAGGAGCGAAAGCGTGGGGAGCGAACAGGATTAGATACCCTGG
TAGTCCACGCCGTAAACGGTGGGCACTAGGTGTGGGCAACATTCCACGTTGTCCGTGCCGCAGCTAA
CGCATTAAGTGCCCCGCCTGGGGAGTACGGCCGCAAGGCTAAAACTCAAAGGAATTGACGGGGGCCC
GCACAAGCGGCGGAGCATGTGGCTTAATTCGACGCAACGCGAAGAACCTTACCAAGGCTTGACATAC
ACCGGAAACGTCTGGAGACAGGCGCCCCCTTGTGGTCGGTGTACAGGTGGTGCATGGCTGTCGTCAG
CTCGTGTCGTGAGATGTTGGGTTAAGTCCCGCAACGAGCGCAACCCTTGTCCCGTGTTGCCAGCAGG
CCCTTGTGGTGCTGGGGACTCACGGGAGACCGCCGGGGTCAACTCGGAGGAAGGTGGGGACGACGT
CAAGTCATCATGCCCCTTATGTCTTGGGCTGCACACGTGCTACAATGGCCGGTACAATGAGCTGCGAT
ACCGCGAGGTGGAGCGAATCTCAAAAAGCCGGTCTCAGTTCGGATTGGGGTCTGCAACTCGACCCCA
TGAAGTCGGAGTCGCTAGTAATCGCAGATCAGCATTGCTGCGGTGAATACGTTCCCGGGCCTTGTACA
CACCGCCCGTCACGTCACGAAAGTCGGTAACACCCGAAGCCGGTGGCCCAACCCCTTGTGGGAGGG
AGCTGTCGAAGGTGGGACTGGCGATTGGGACGAAGTCGTAACAAGGTAGCCGTACCGGAAGTGCGT
GGAAATCCCCCCTCCTTAAAGGGGG

Figure S4 ^{1}H NMR spectrum of **4** in pyridine-d_5 (600 MHz)

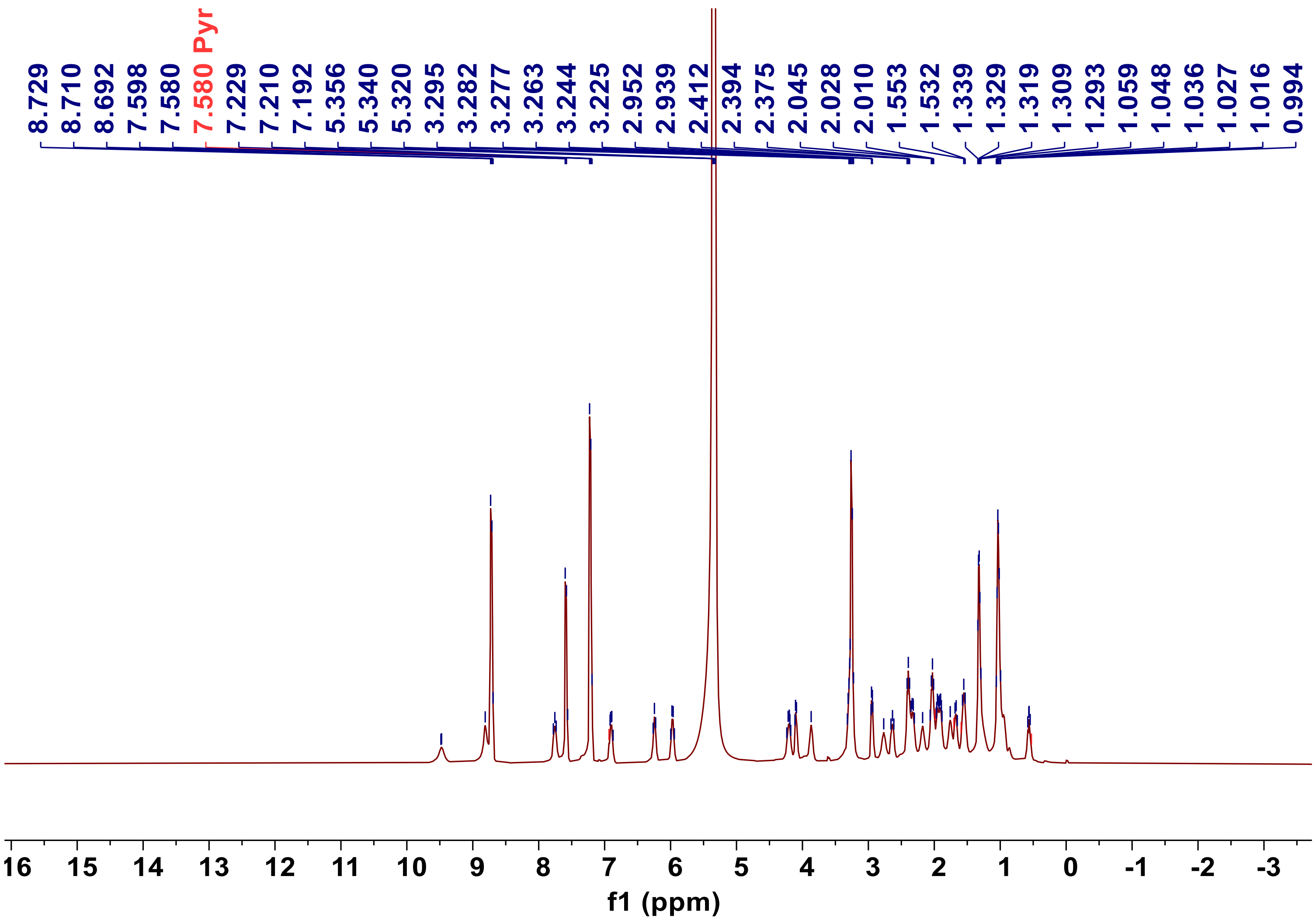

Figure S5 ^{13}C NMR spectrum of **4** in pyridine-d_5 (150 MHz)

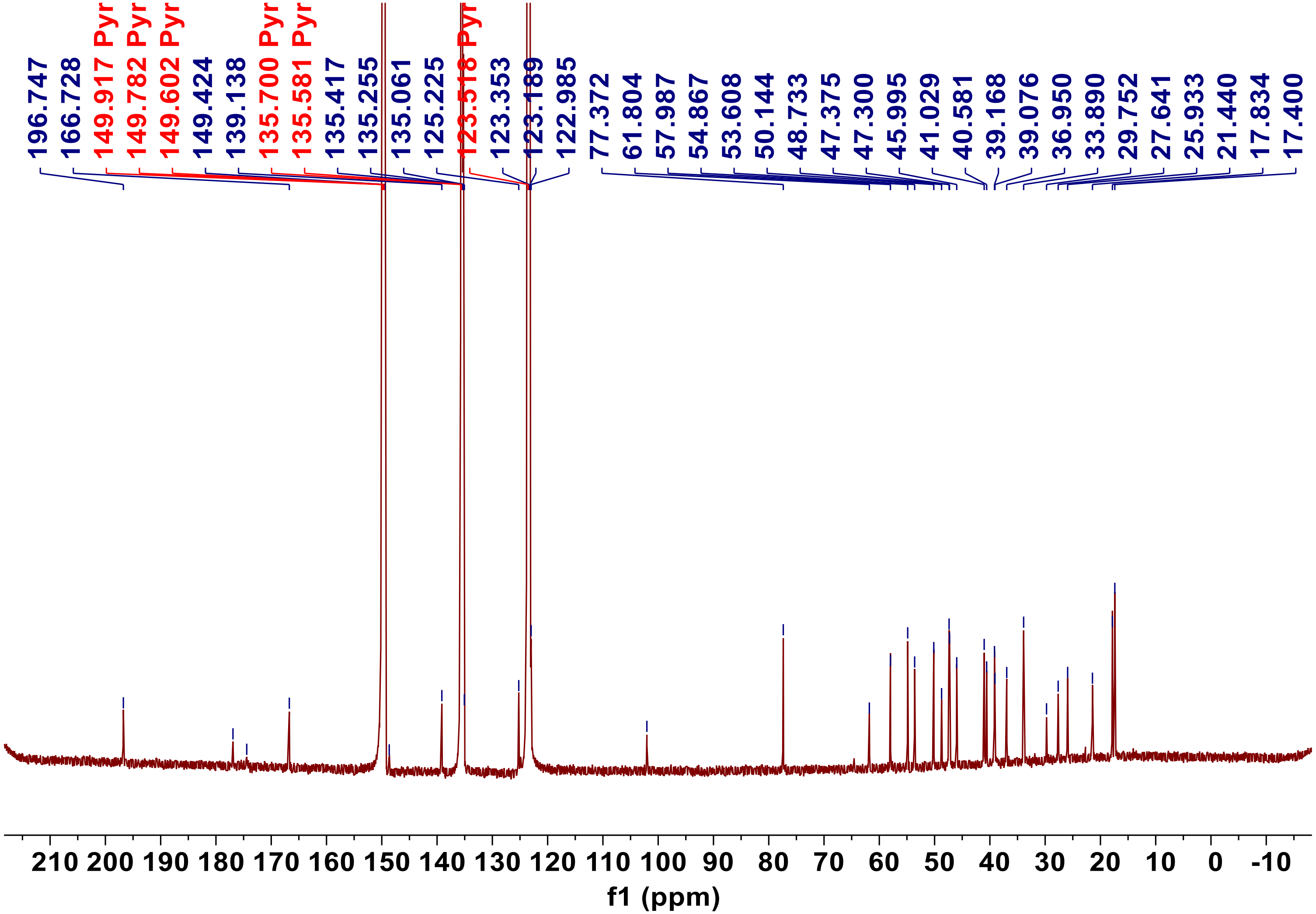

Figure S6 DEPT-90 spectrum of **4**

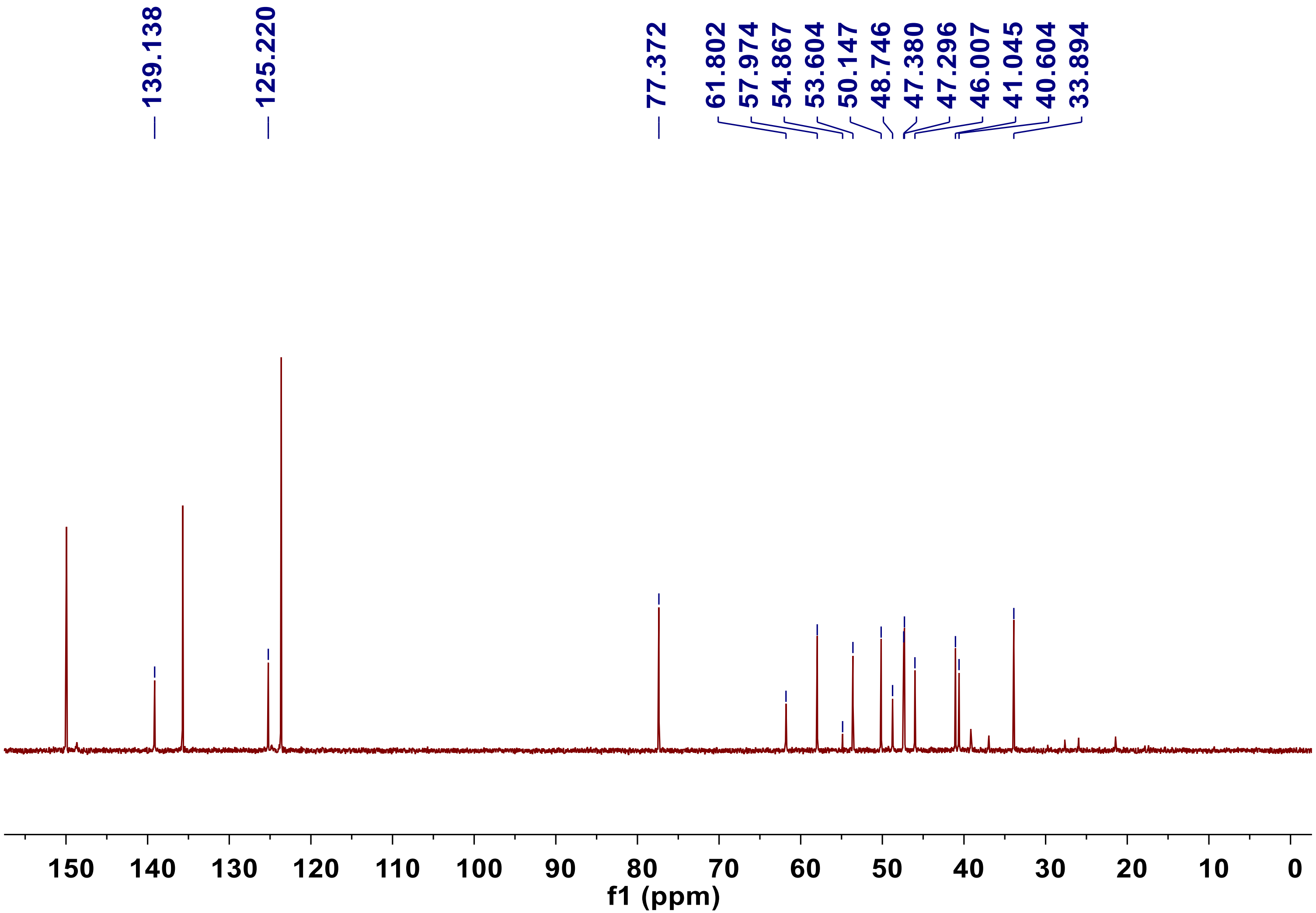

Figure S7 DEPT-135 spectrum of **4**

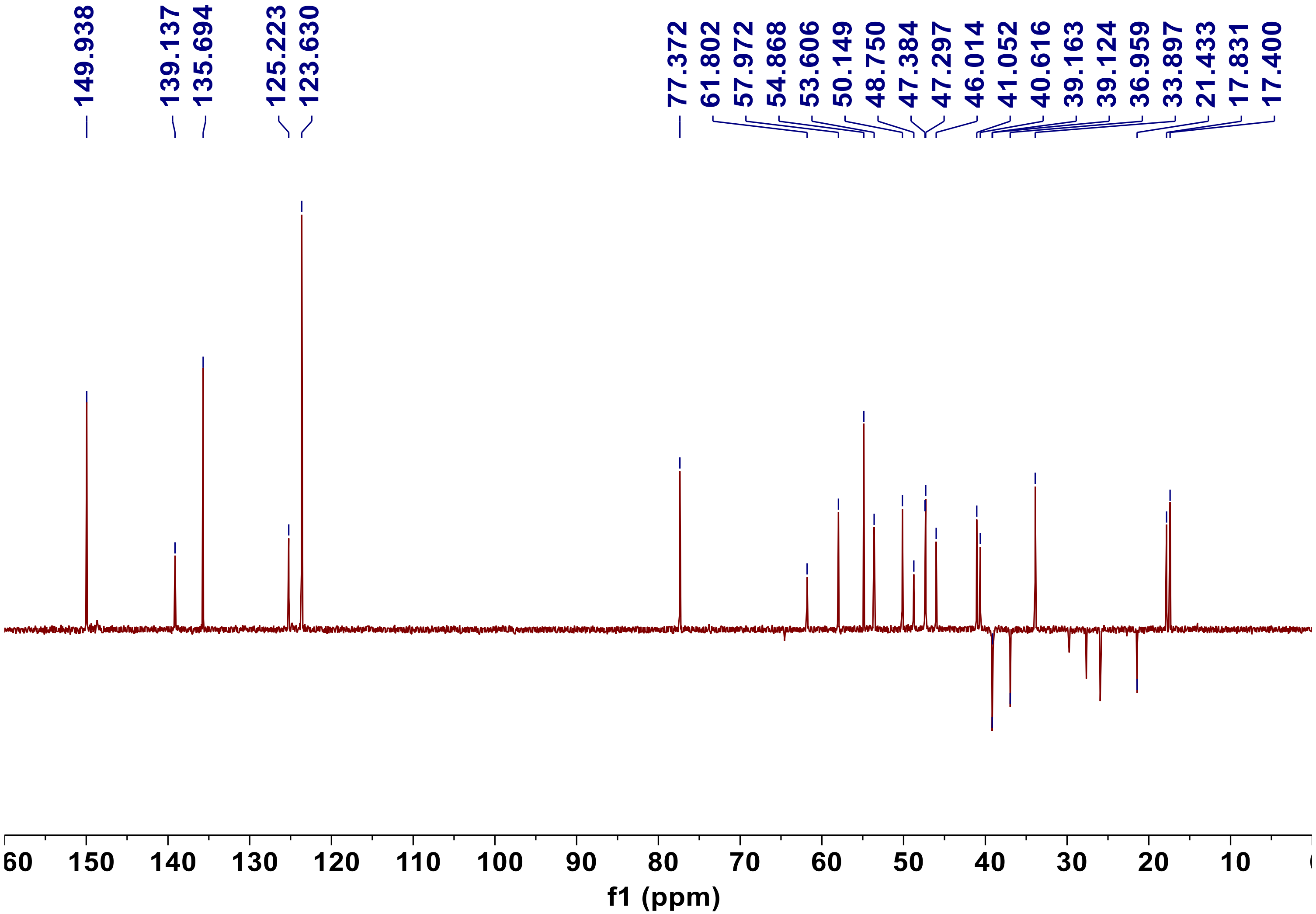

Figure S8 HSQC spectrum of **4**

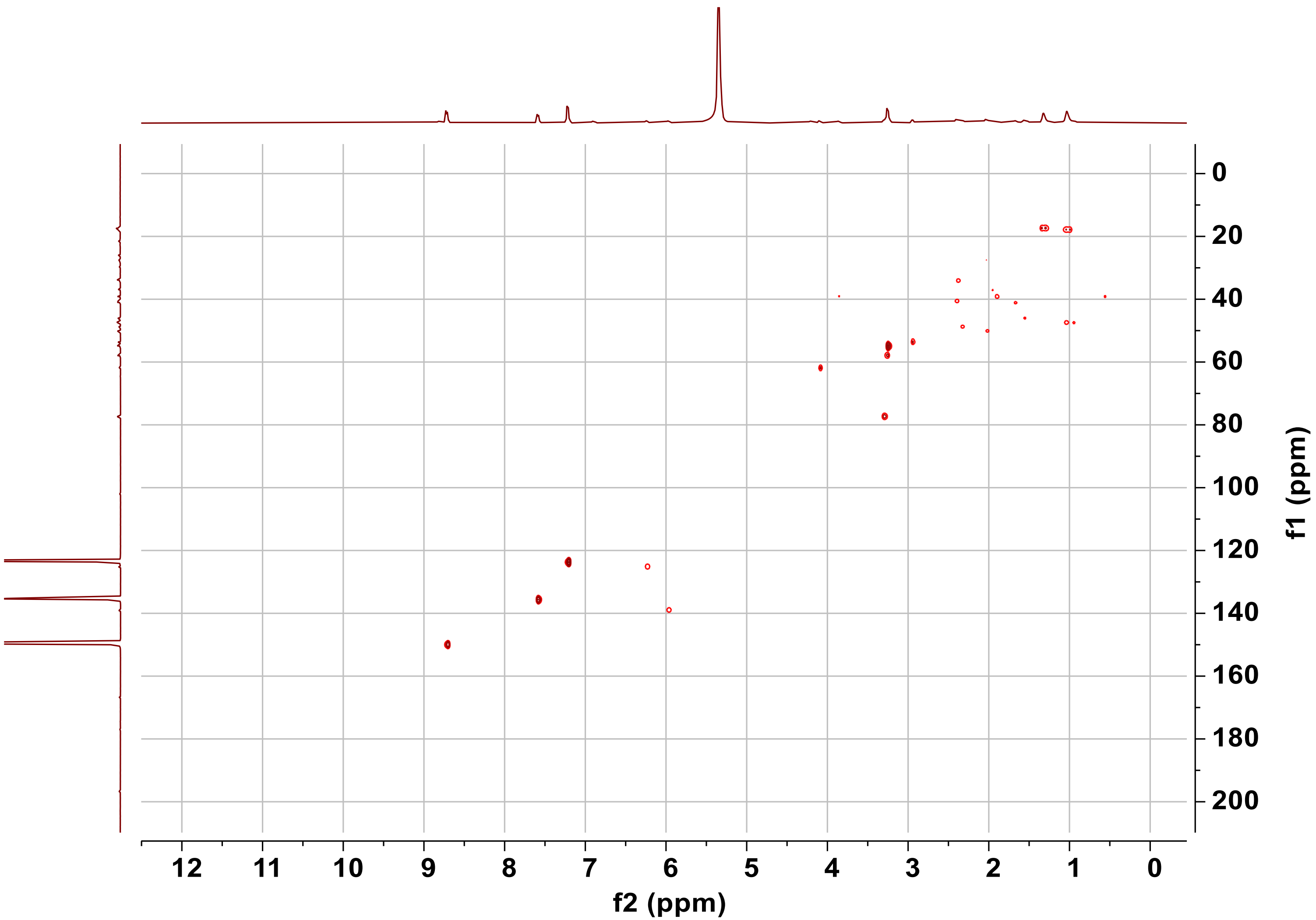

Figure S9 HMBC spectrum of **4**

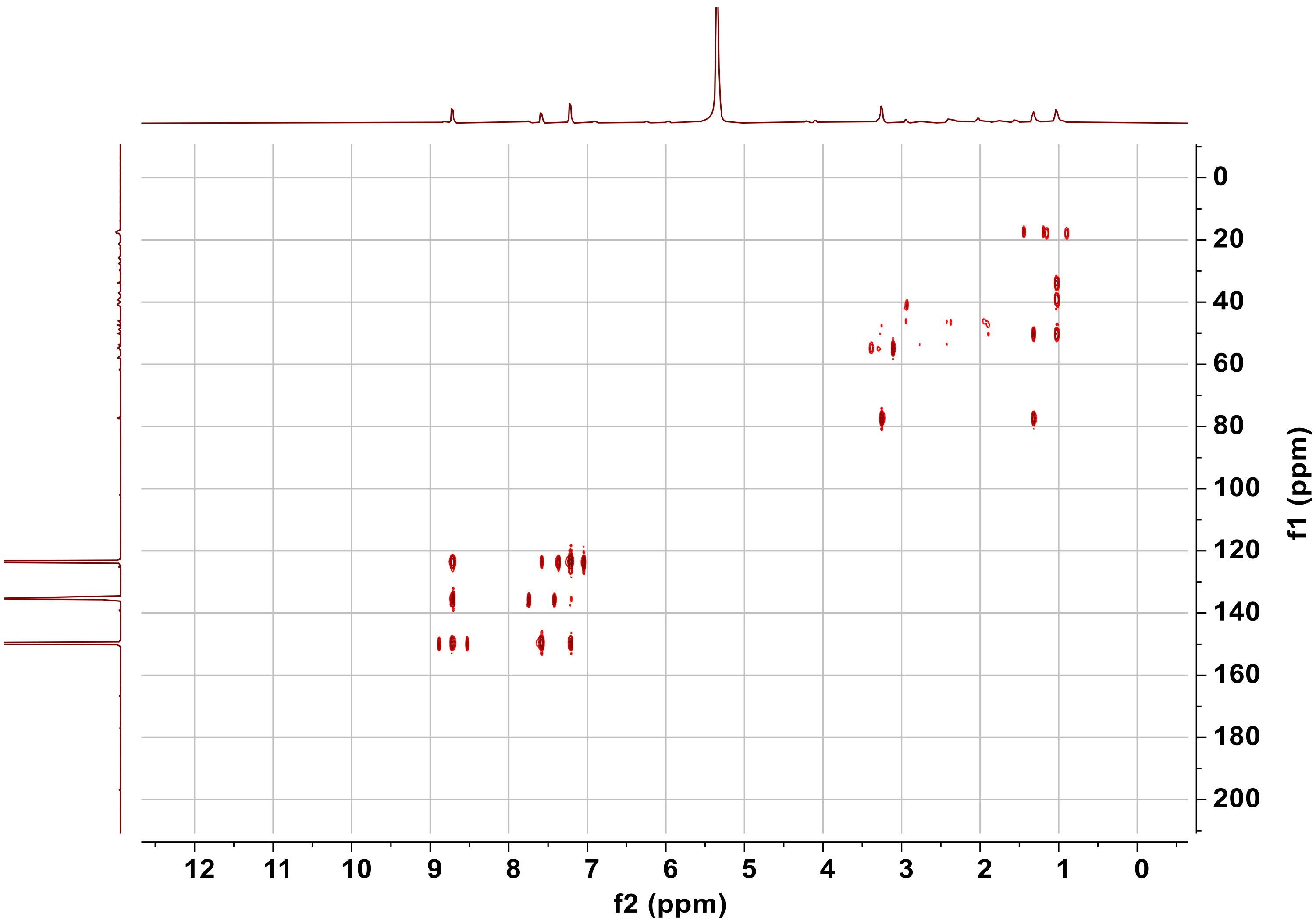

Figure S10 ^{1}H-^{1}H COSY spectrum of **4**

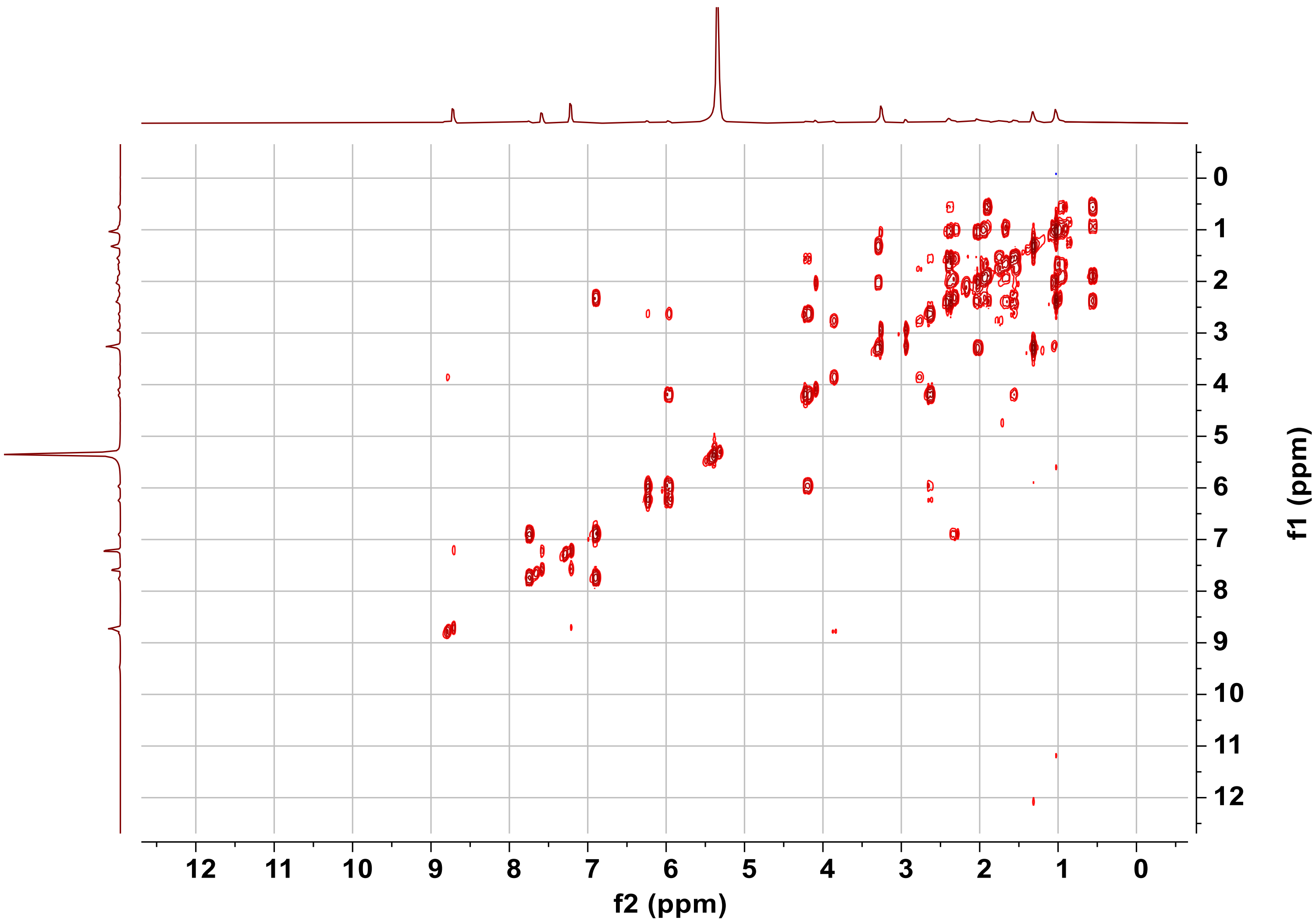

Figure S11 NOESY spectrum of **4**

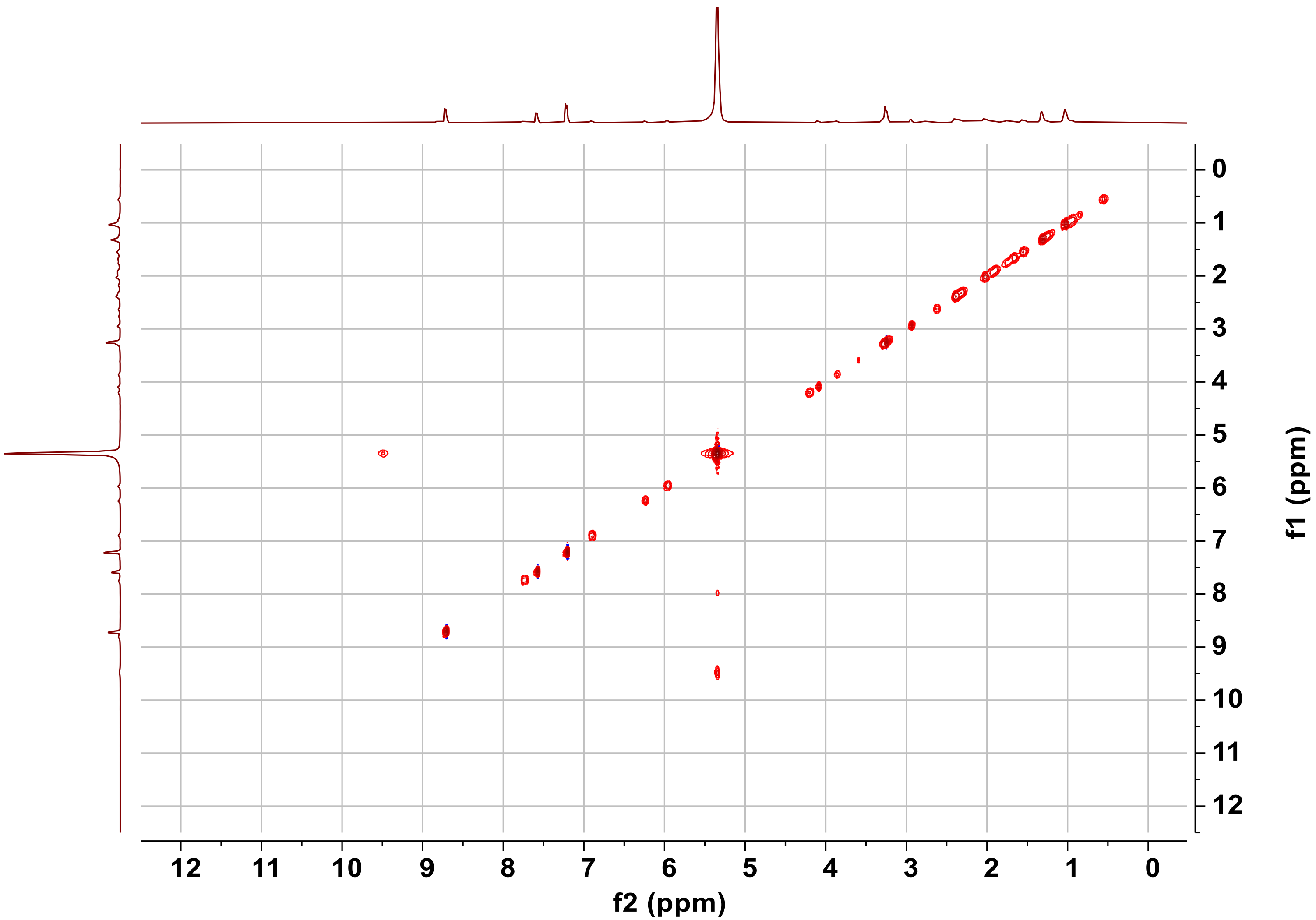

Figure S12 HRESIMS spectrum of **4**

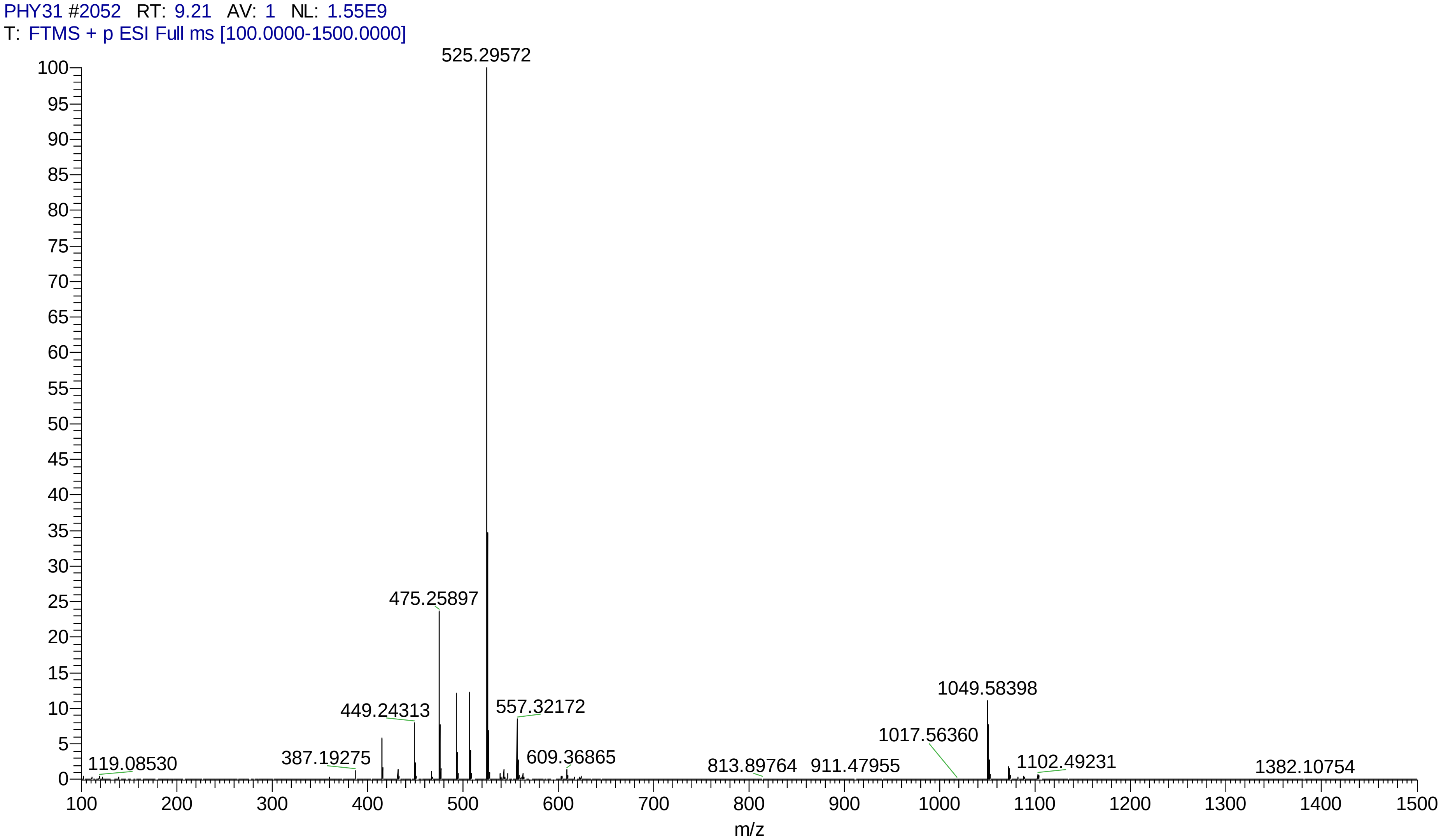

Figure S13 UV spectrum of **4**

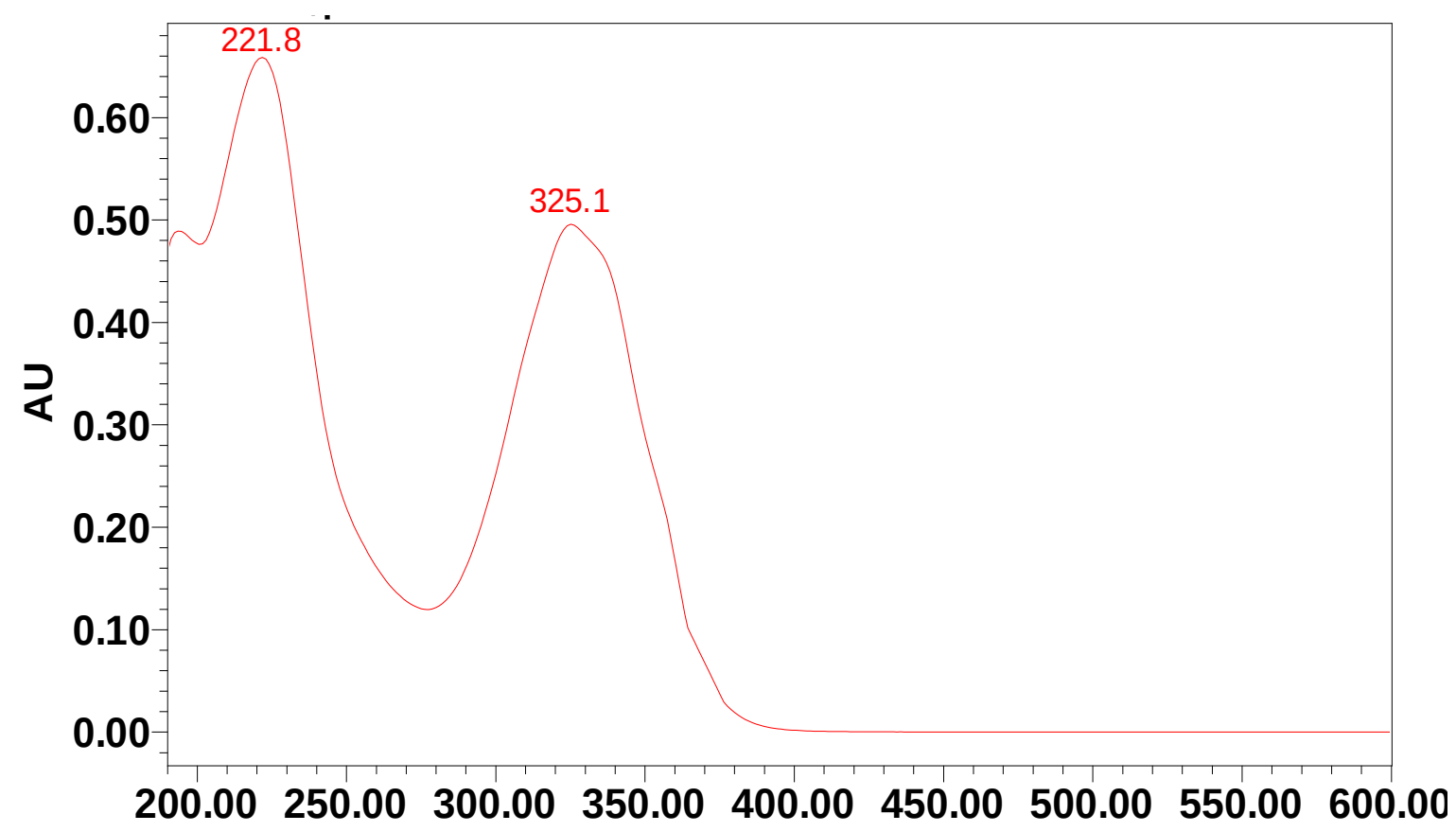

No.	Wavelength (nm)	Abs
1	221.8	0.659
2	325.1	0.496

Figure S14 ^{1}H NMR spectrum of **5** in pyridine-d_5 (400 MHz)

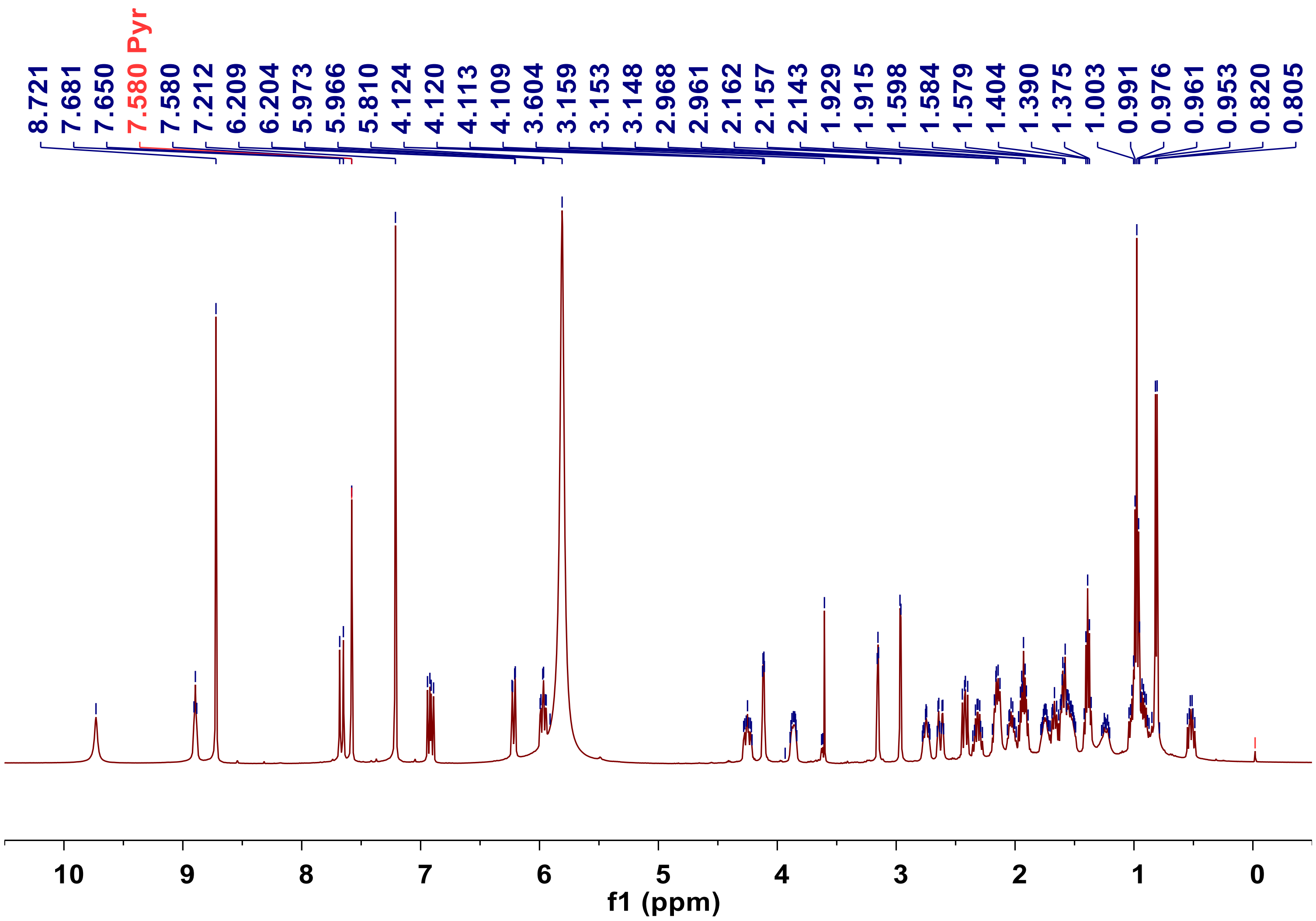

Figure S15 ^{13}C NMR spectrum of **5** in pyridine-d_5 (100 MHz)

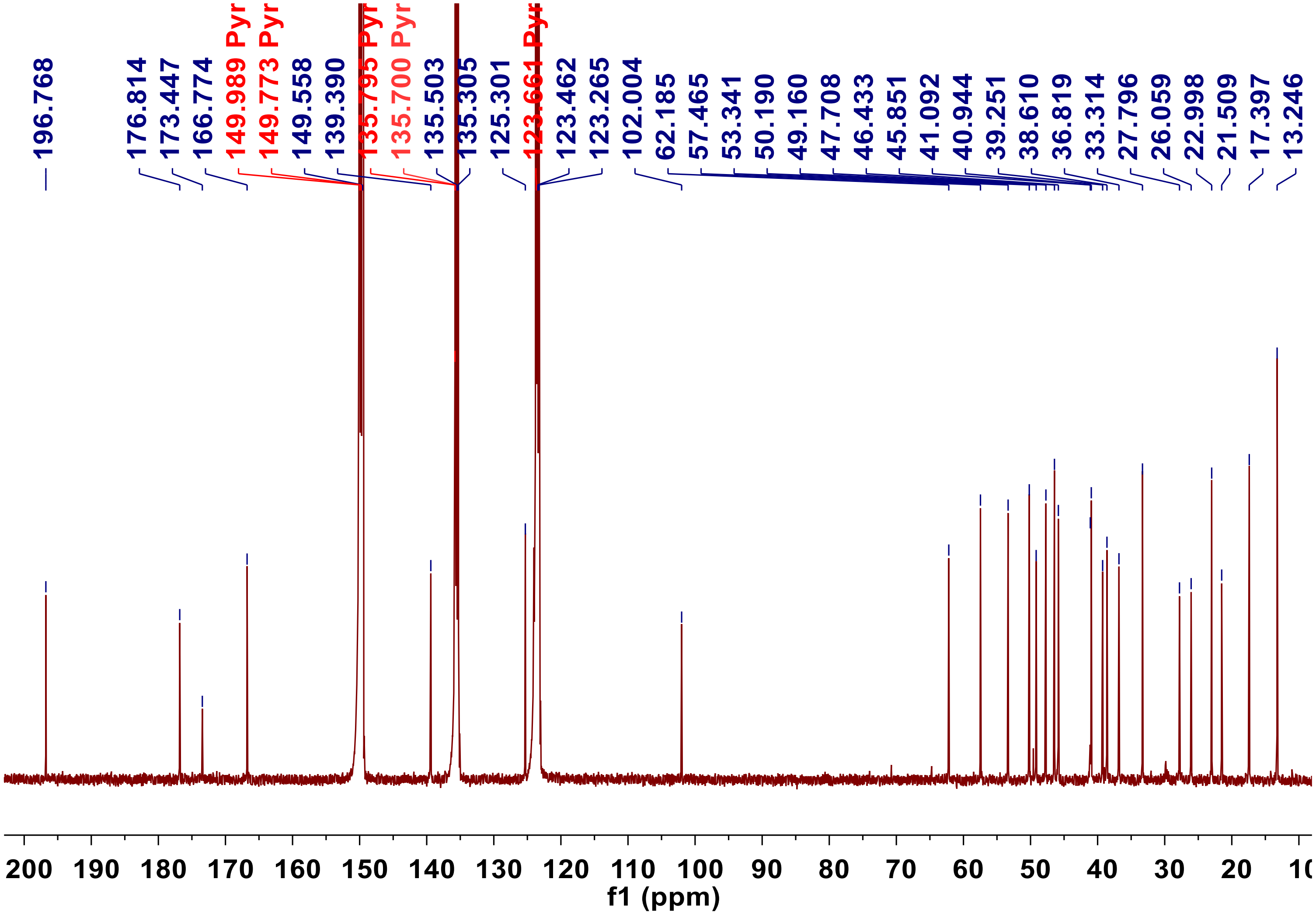

Figure S16 DEPT-90 spectrum of **5**

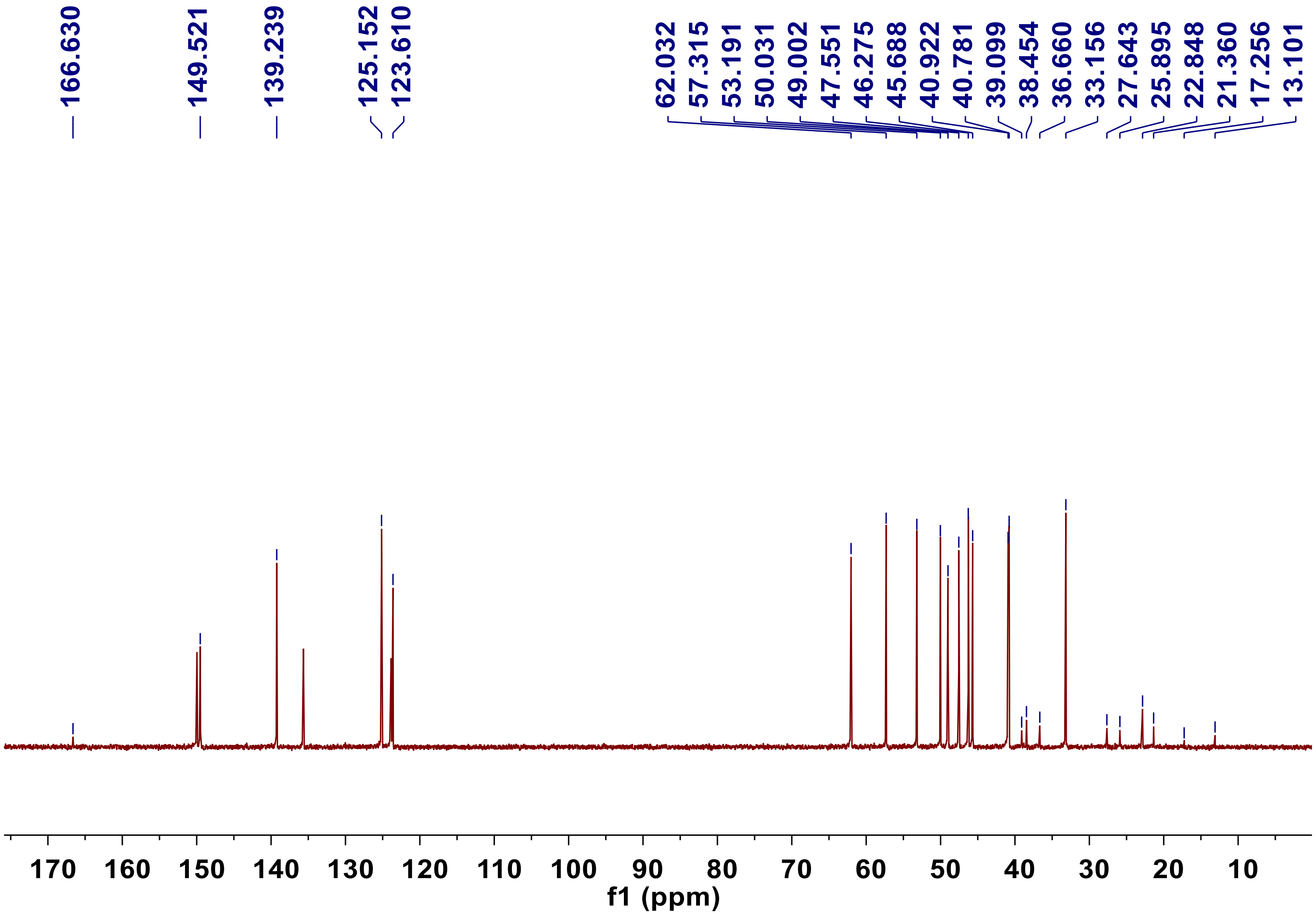

Figure S17 DEPT-135 spectrum of **5**

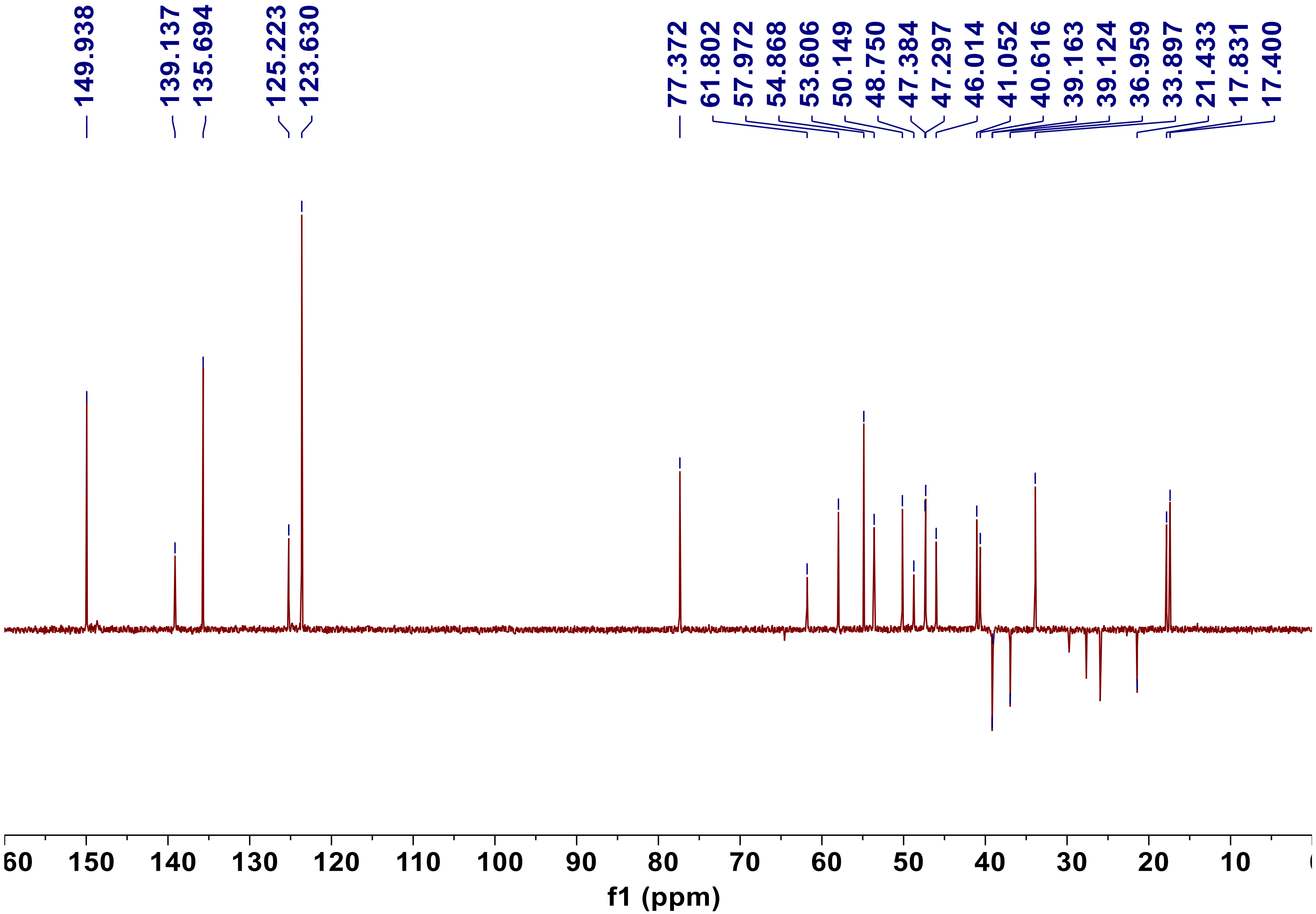

Figure S18 HSQC spectrum of **5**

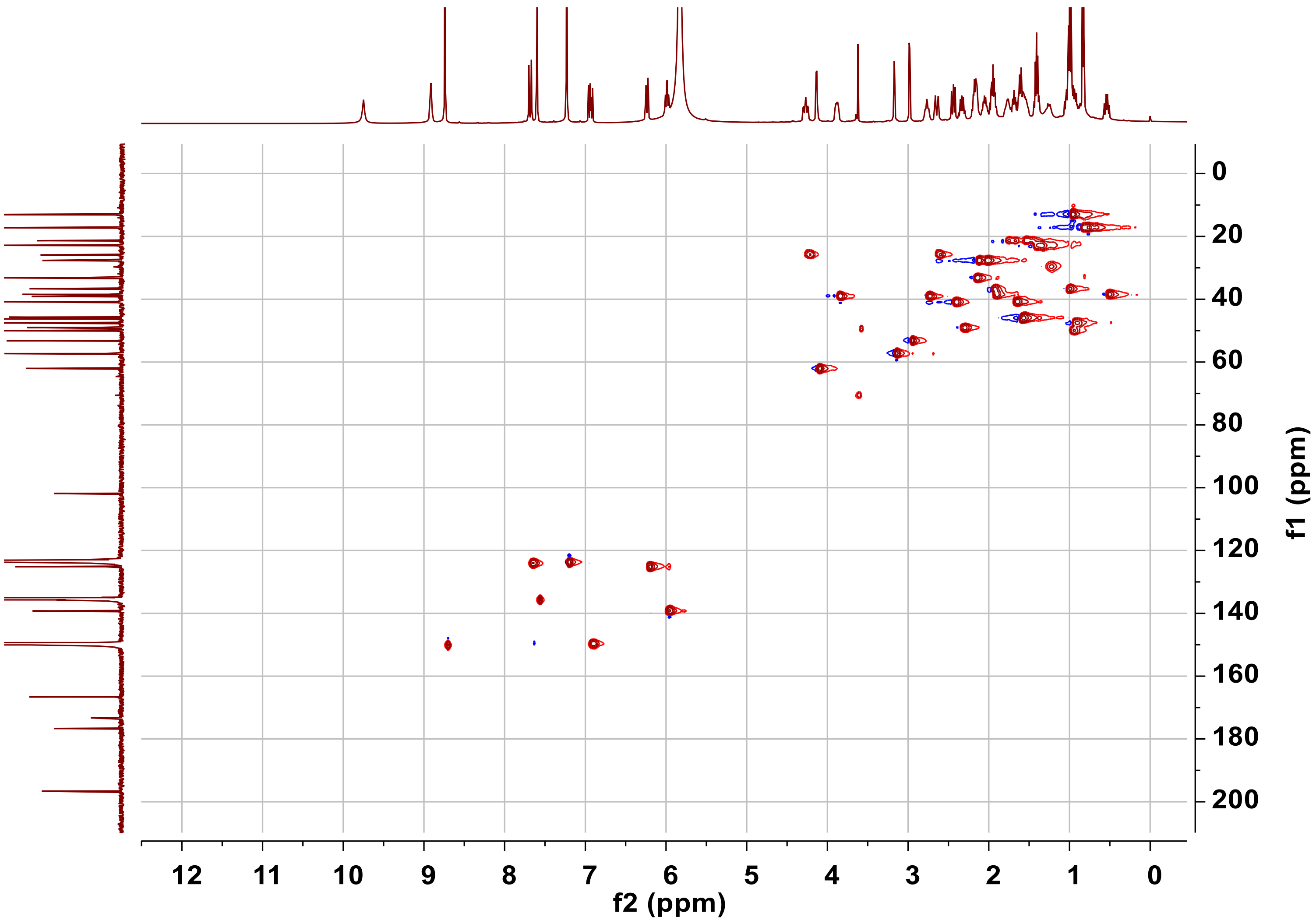

Figure S19 HMBC spectrum of **5**

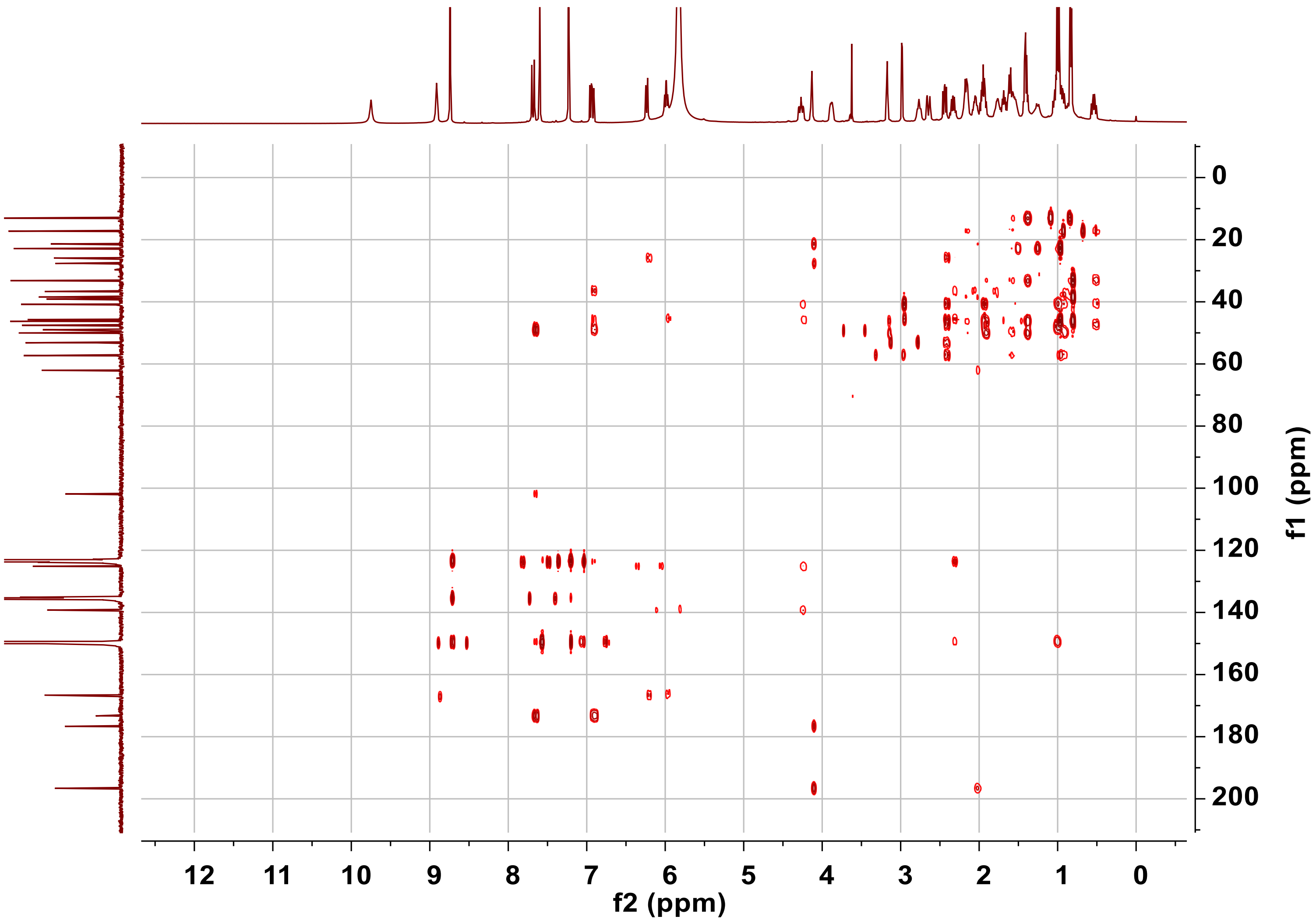

Figure S20 ^{1}H-^{1}H COSY spectrum of **5**

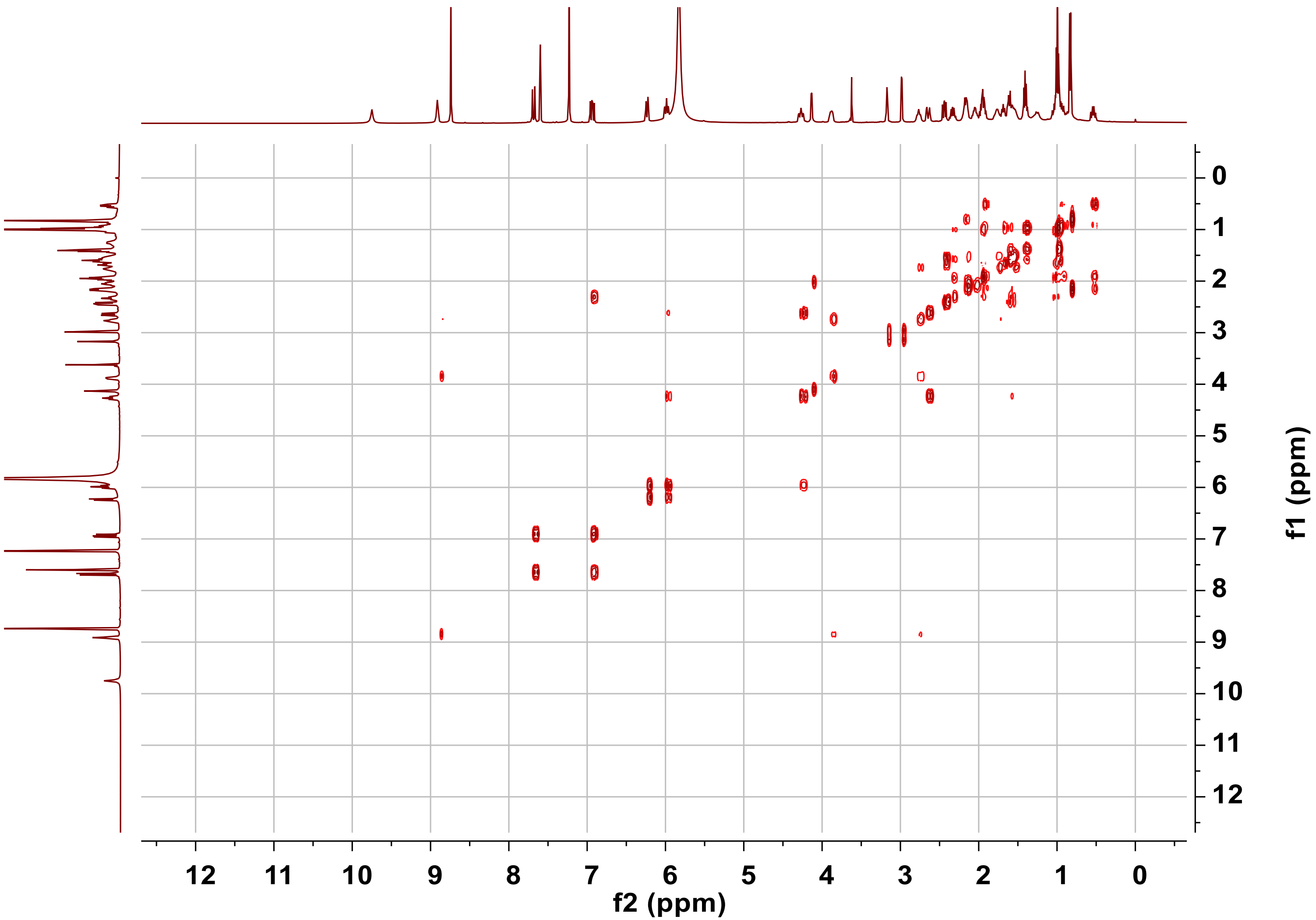

Figure S21 NOESY spectrum of **5**

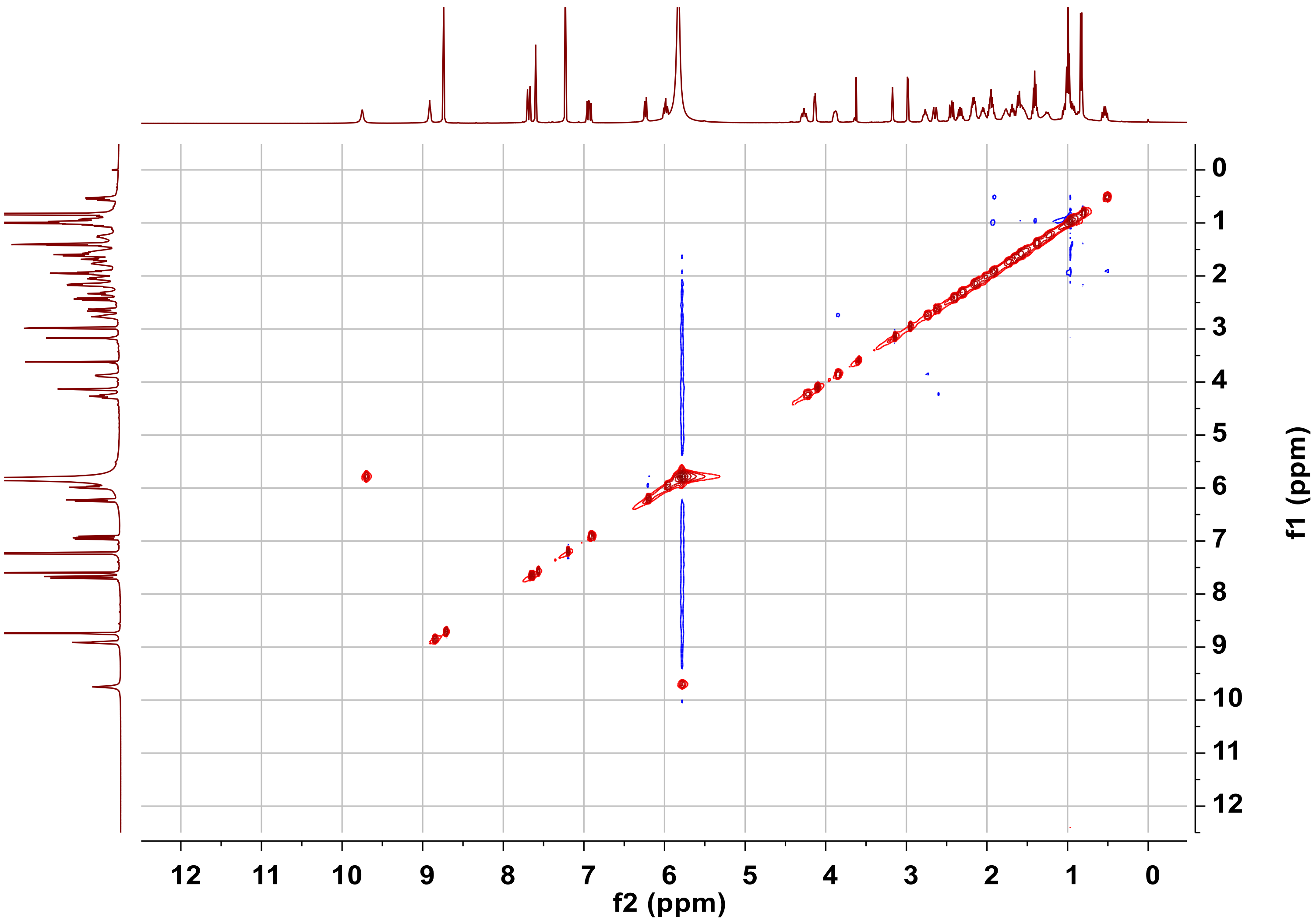

Figure S22 HRESIMS spectrum of **5**

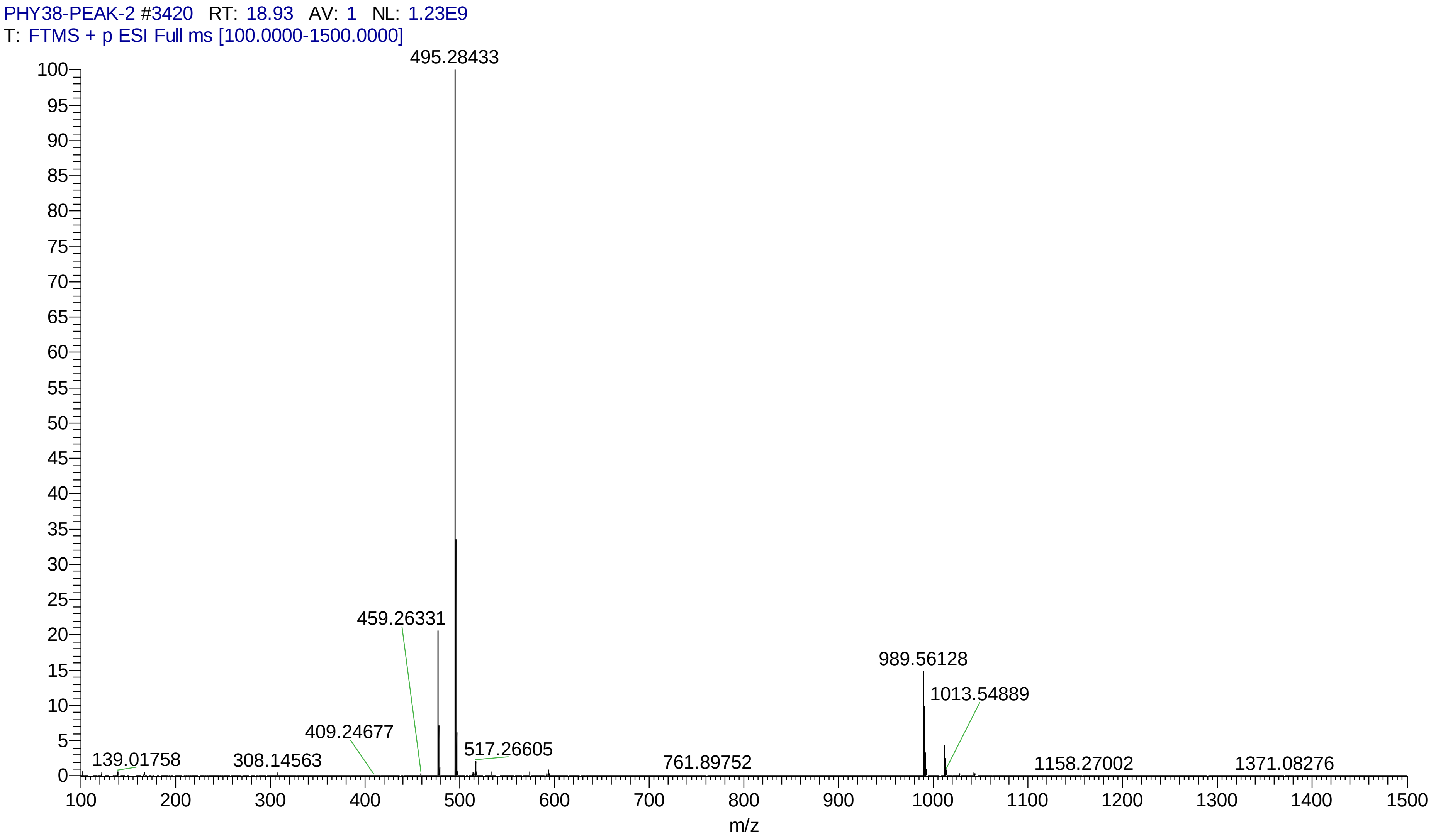

Figure S23 UV spectrum of **5**

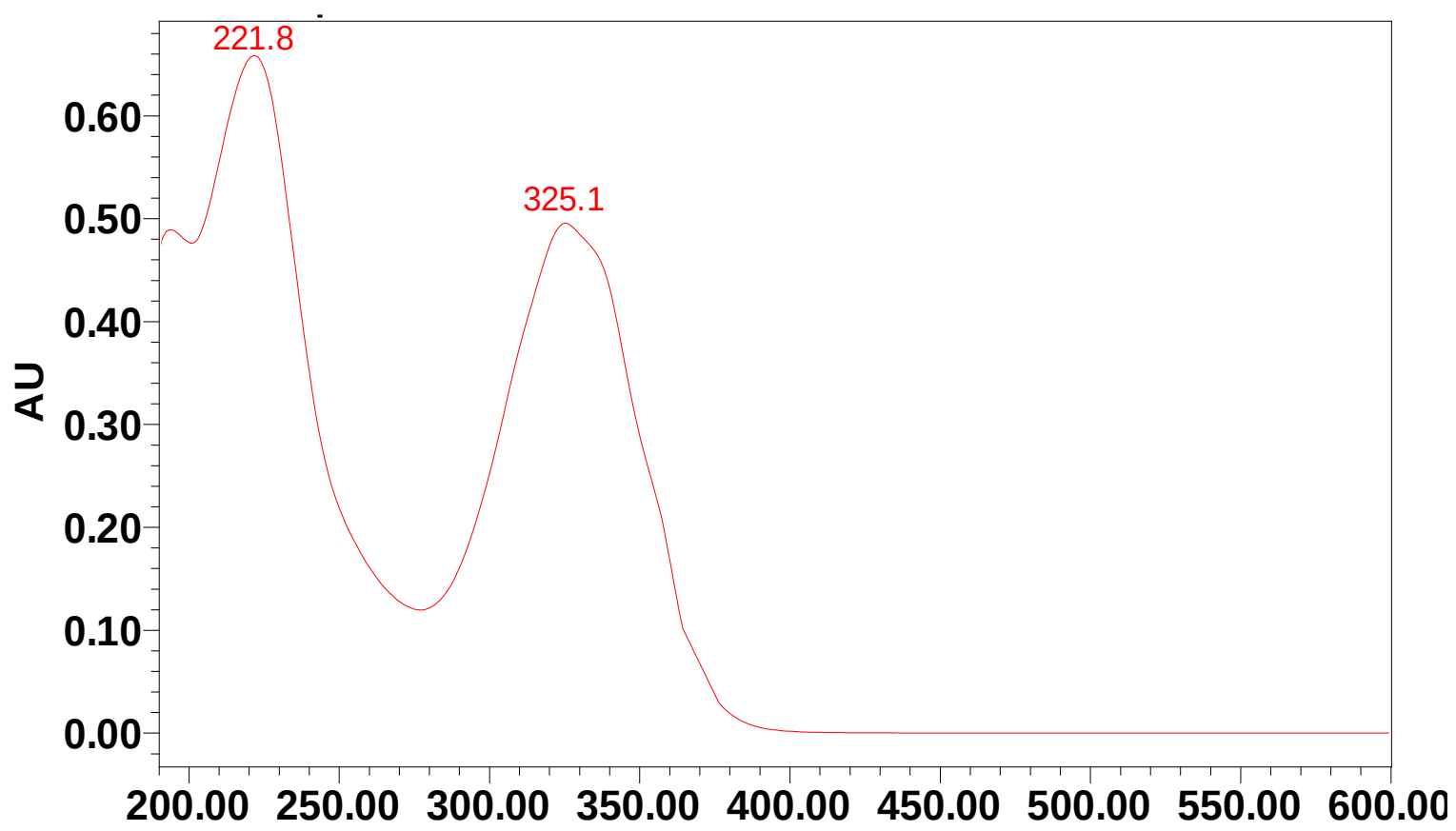

No.	Wavelength (nm)	Abs
1	221.8	0.659
2	325.1	0.496

Figure S24 ^{1}H NMR spectrum of **6** in pyridine-d_5 (600 MHz)

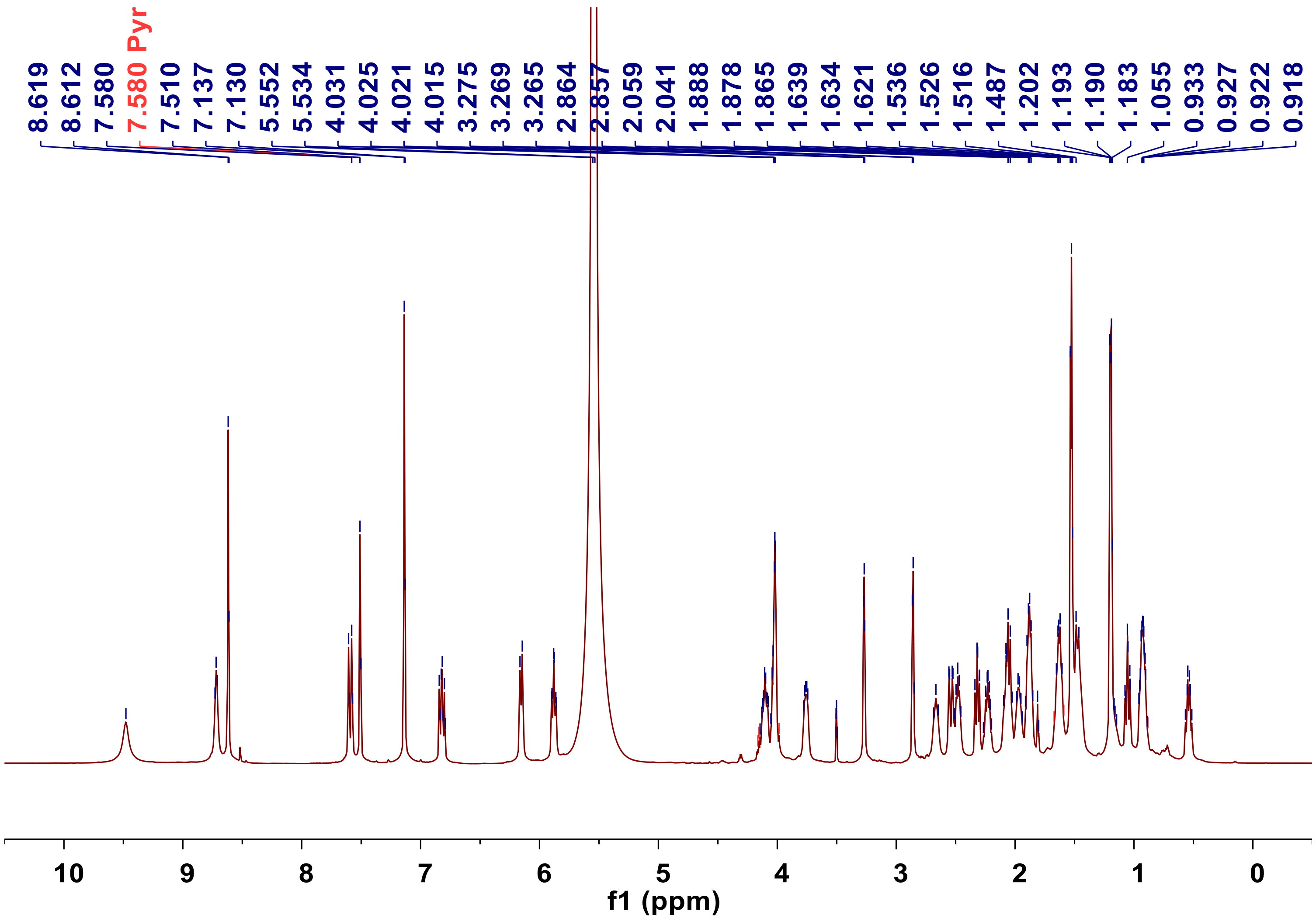

Figure S25 ^{13}C NMR spectrum of **6** in pyridine-d_5 (150 MHz)

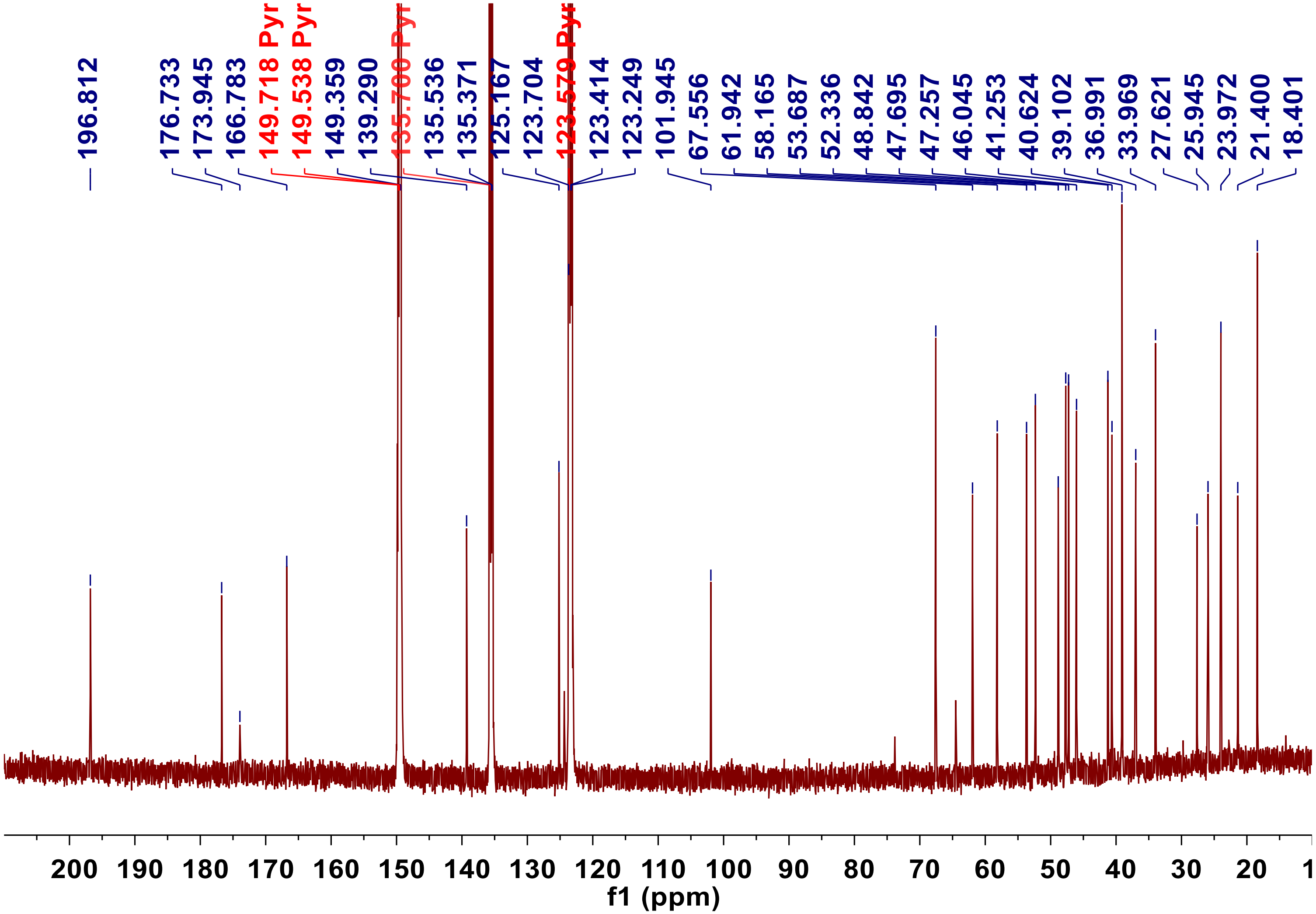

Figure S26 DEPT-135 spectrum of **6**

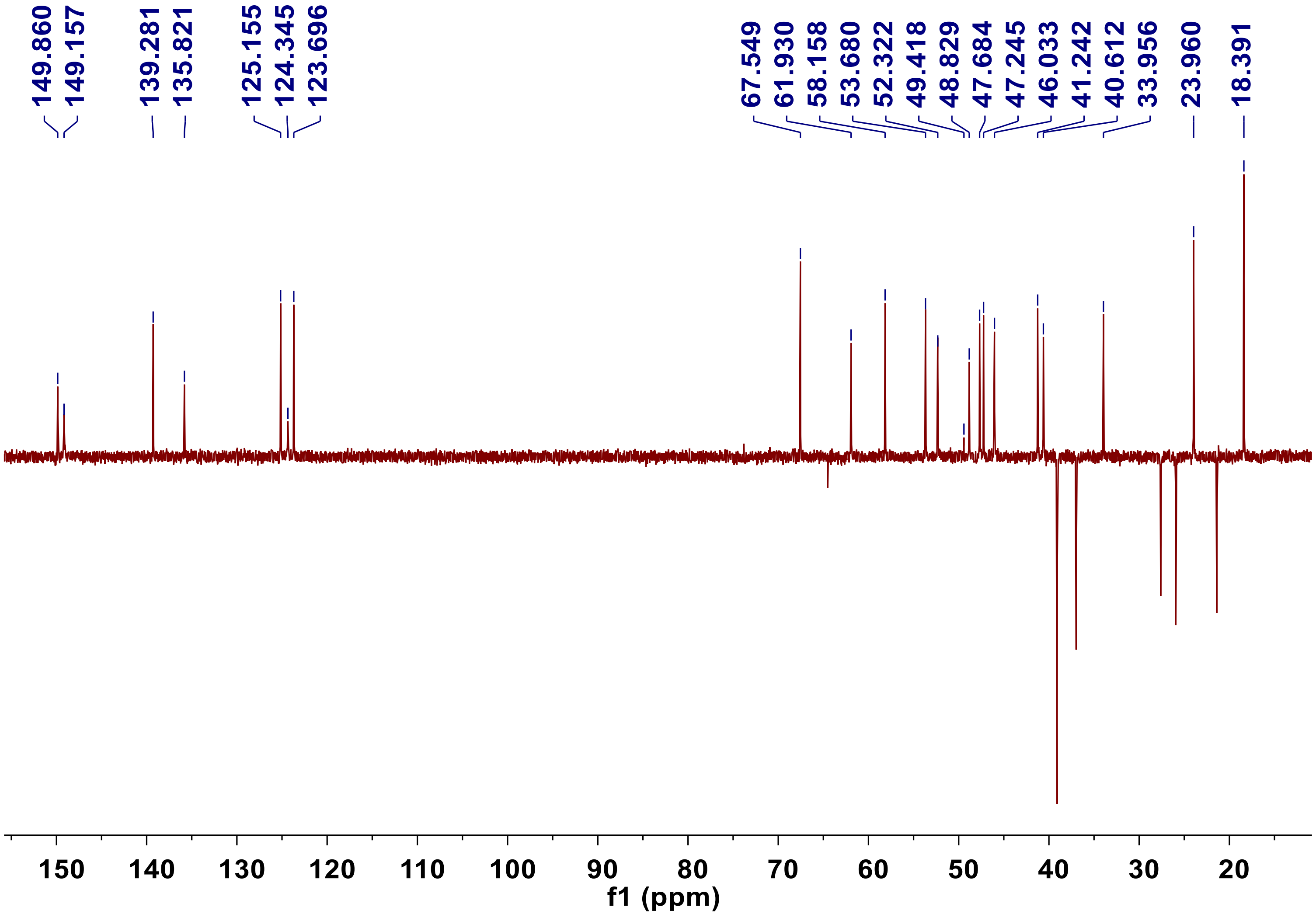

Figure S27 HSQC spectrum of **6**

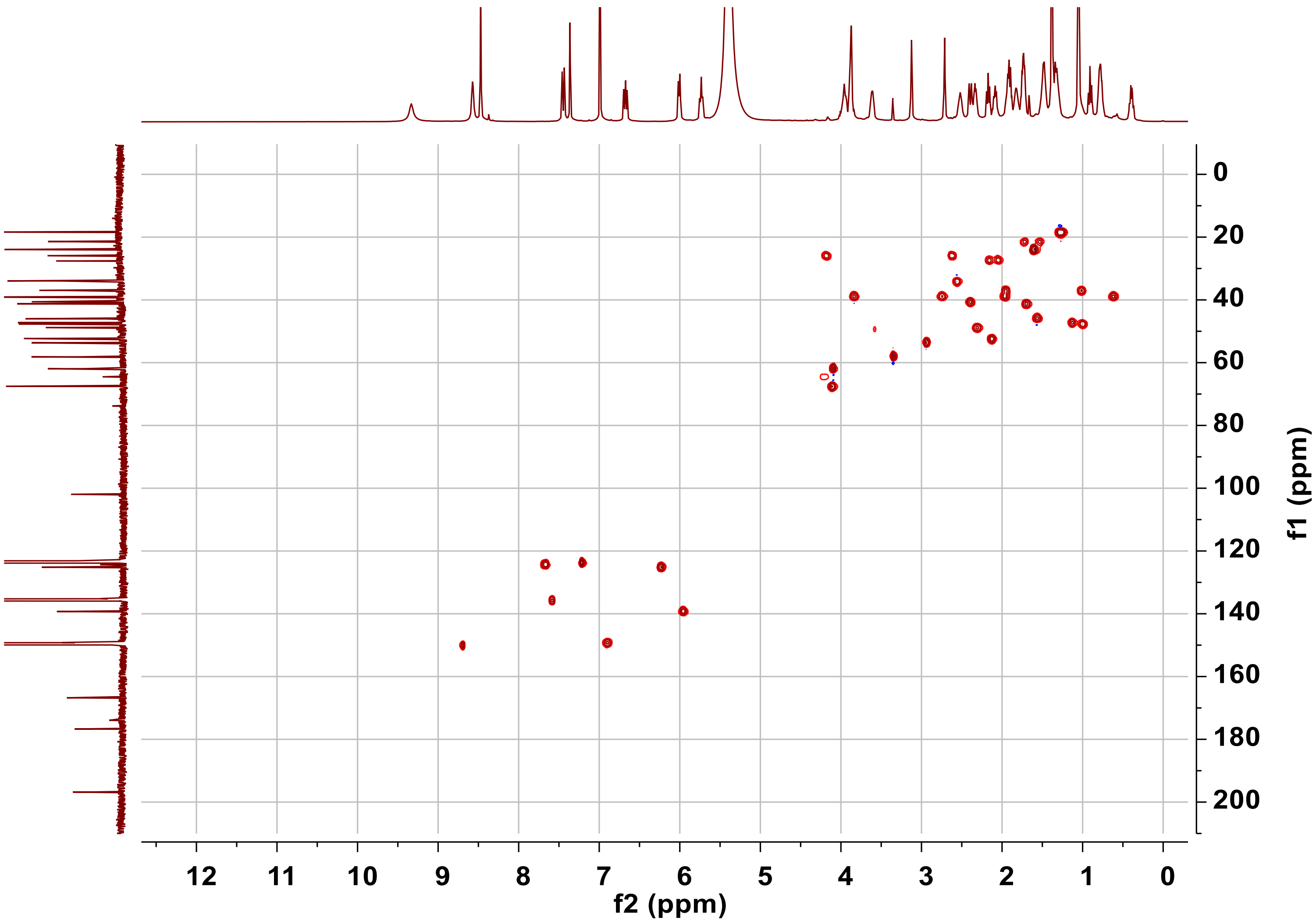

Figure S28 HMBC spectrum of **6**

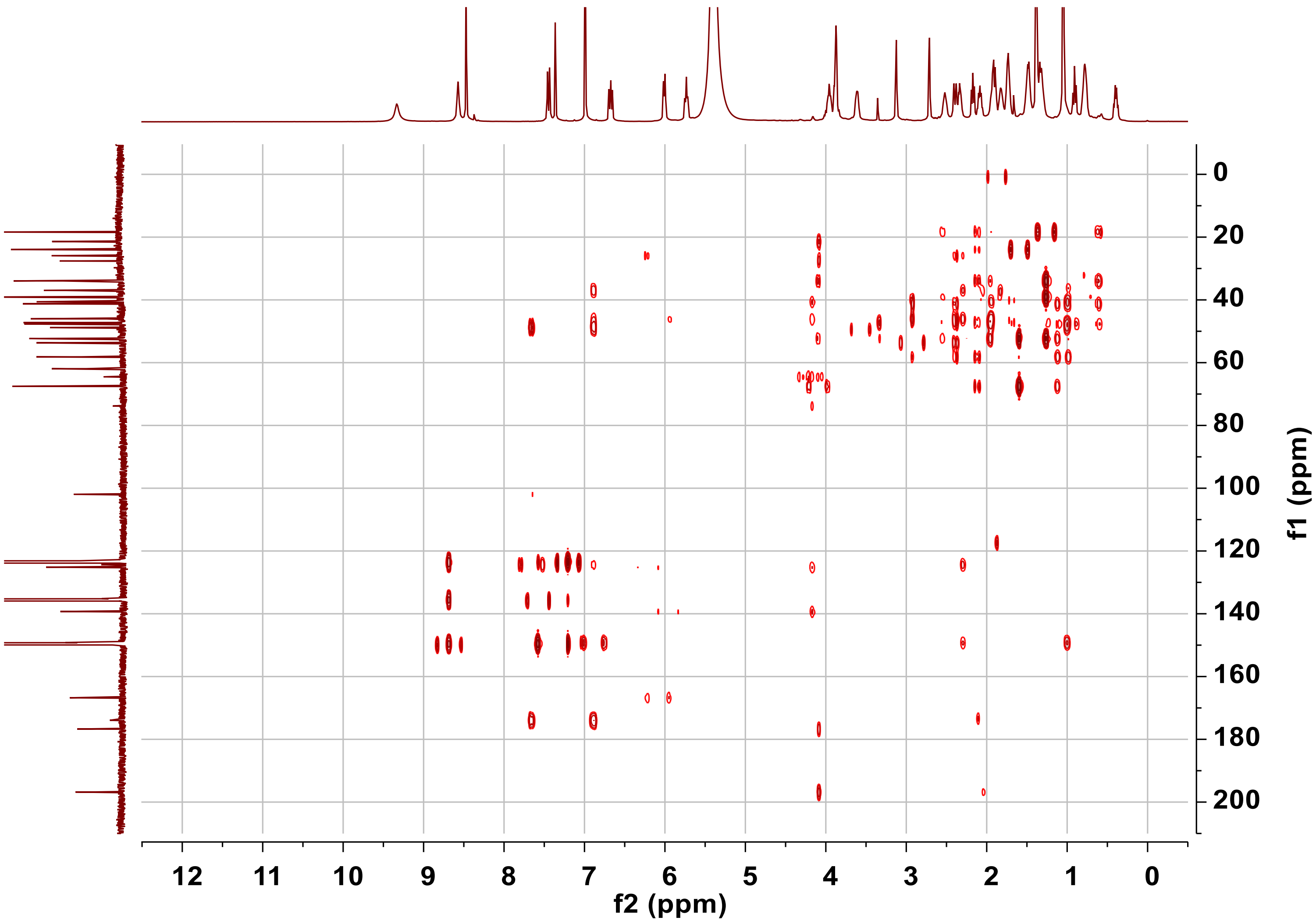

Figure S29 HRESIMS spectrum of **6**

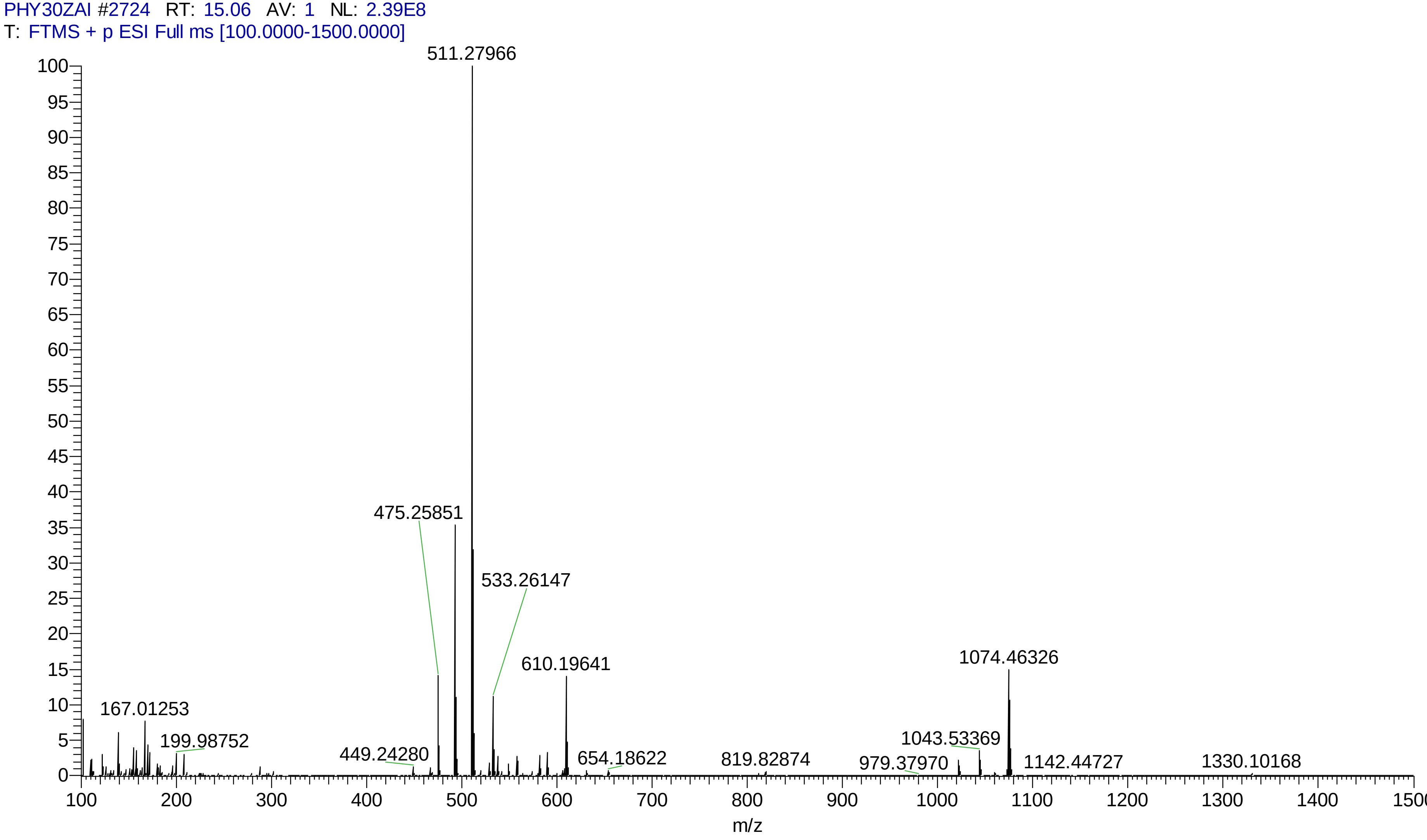

Figure S30 UV spectrum of **6**

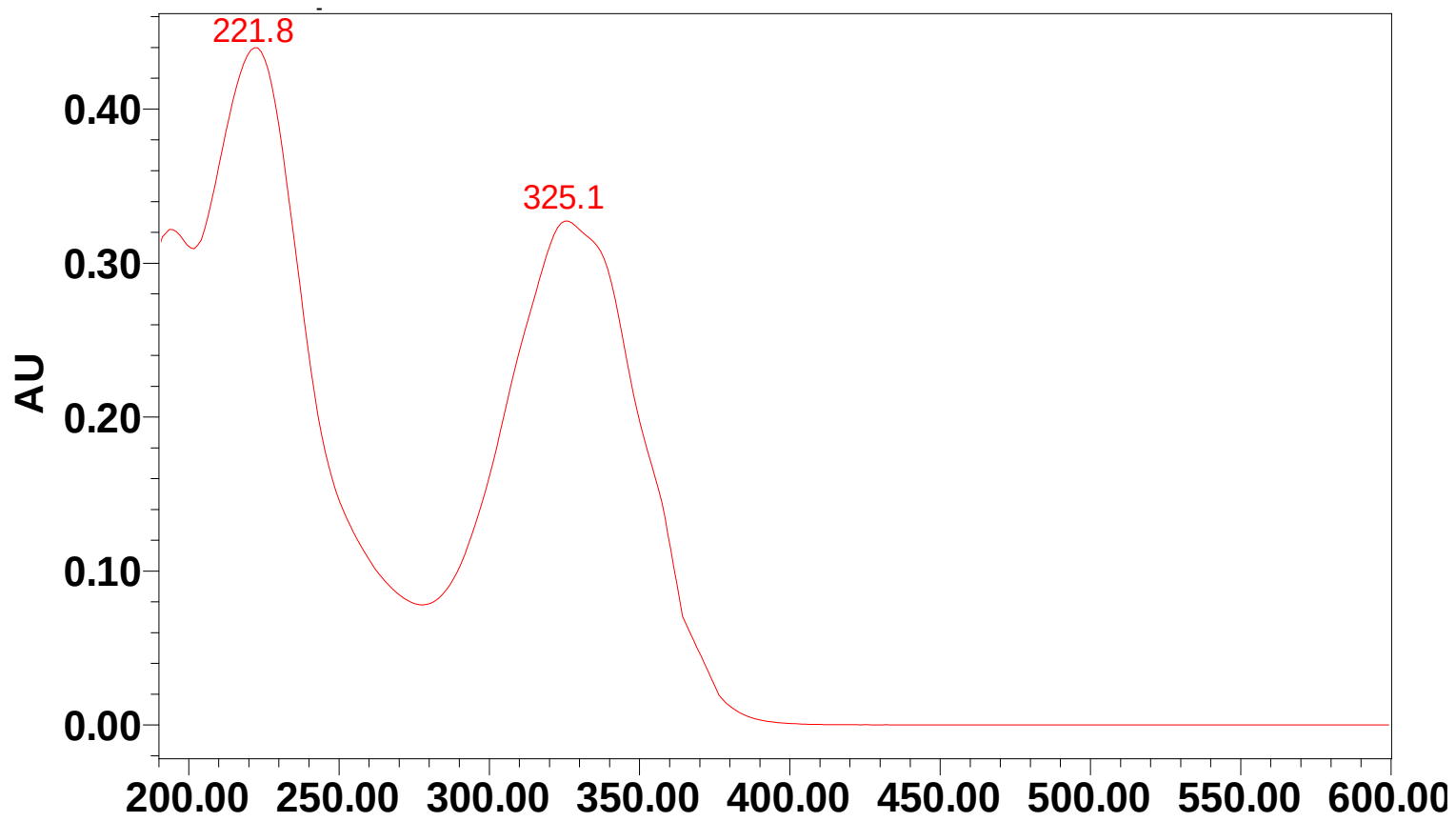

No.	Wavelength (nm)	Abs
1	221.8	0.440
2	325.1	0.327

Figure S31 Inhibition rates of the combination of compounds **4** (A), **5** (B) and acarbose (C) against α-glucosidase in vitro at gradient concentrations

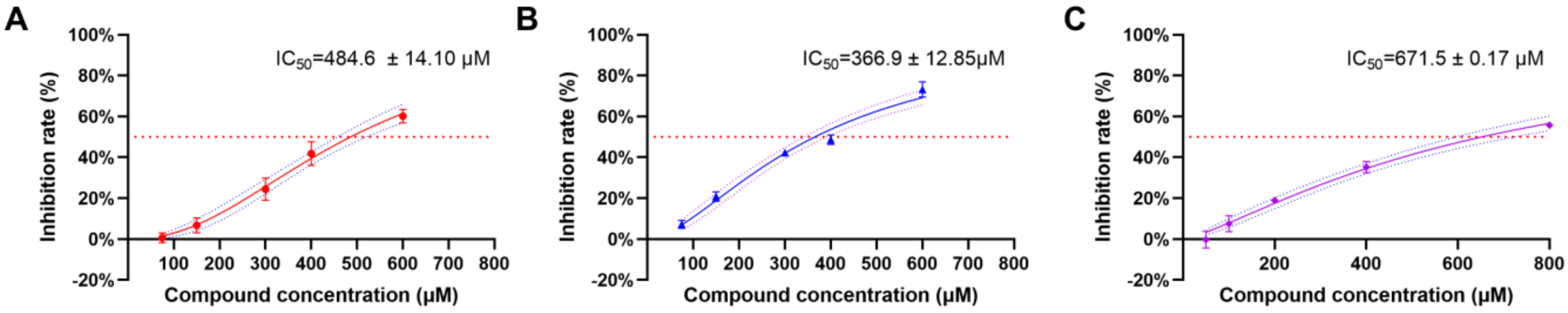

Figure S32 Docking poses and interactions of compound **4** and acarbose with α-glucosidase (PDB ID: 2QMJ).

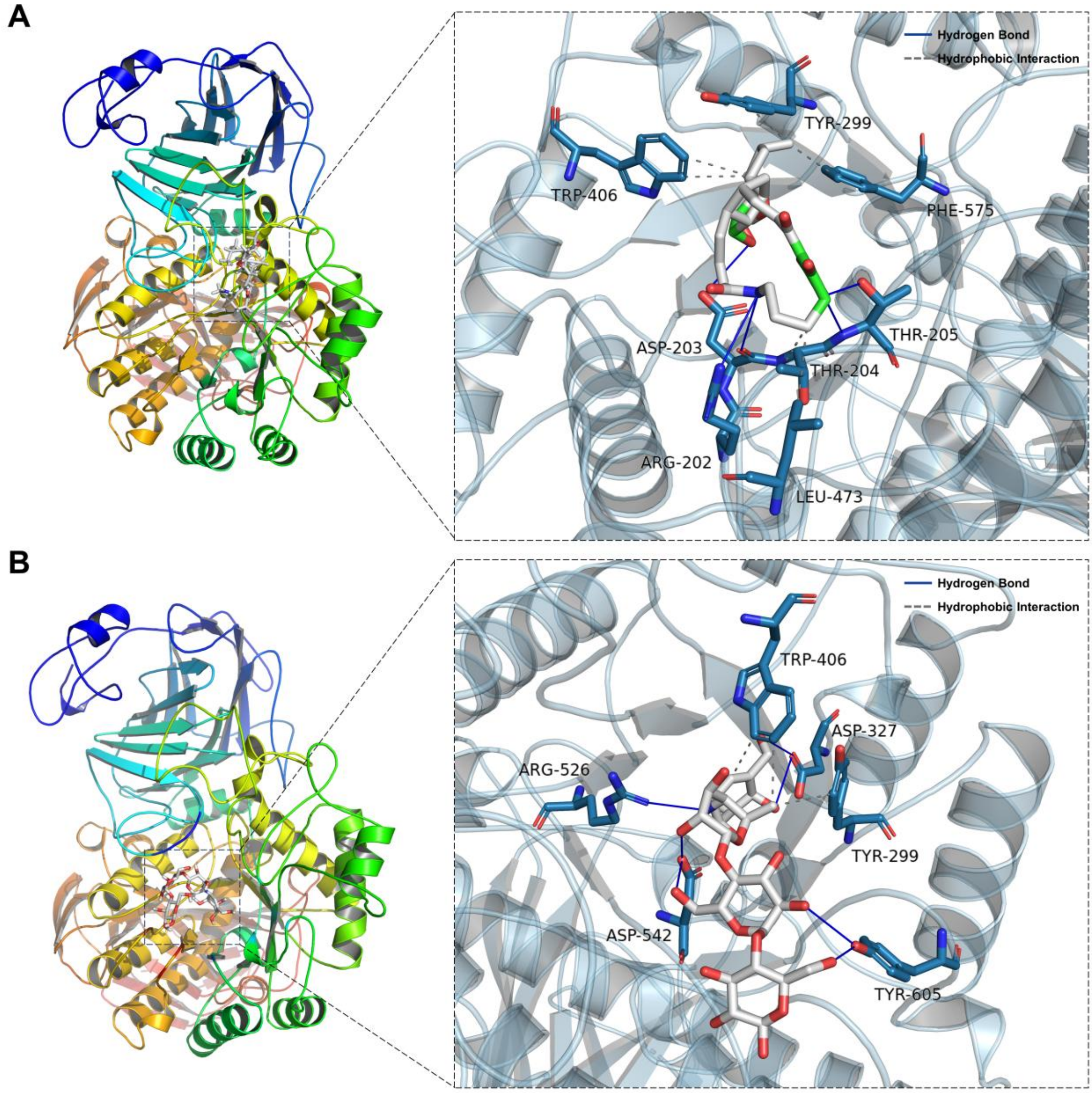

Figure S33 96 well plate assay of **4**–**6** against MRSA (A), *S. aureus* ATCC 29213 (B), *Klebsiella pneumonia* ATCC 113 (C) and *Pseudomonas aeruginosa* ATCC 9027 (D) using the microbroth dilution method.

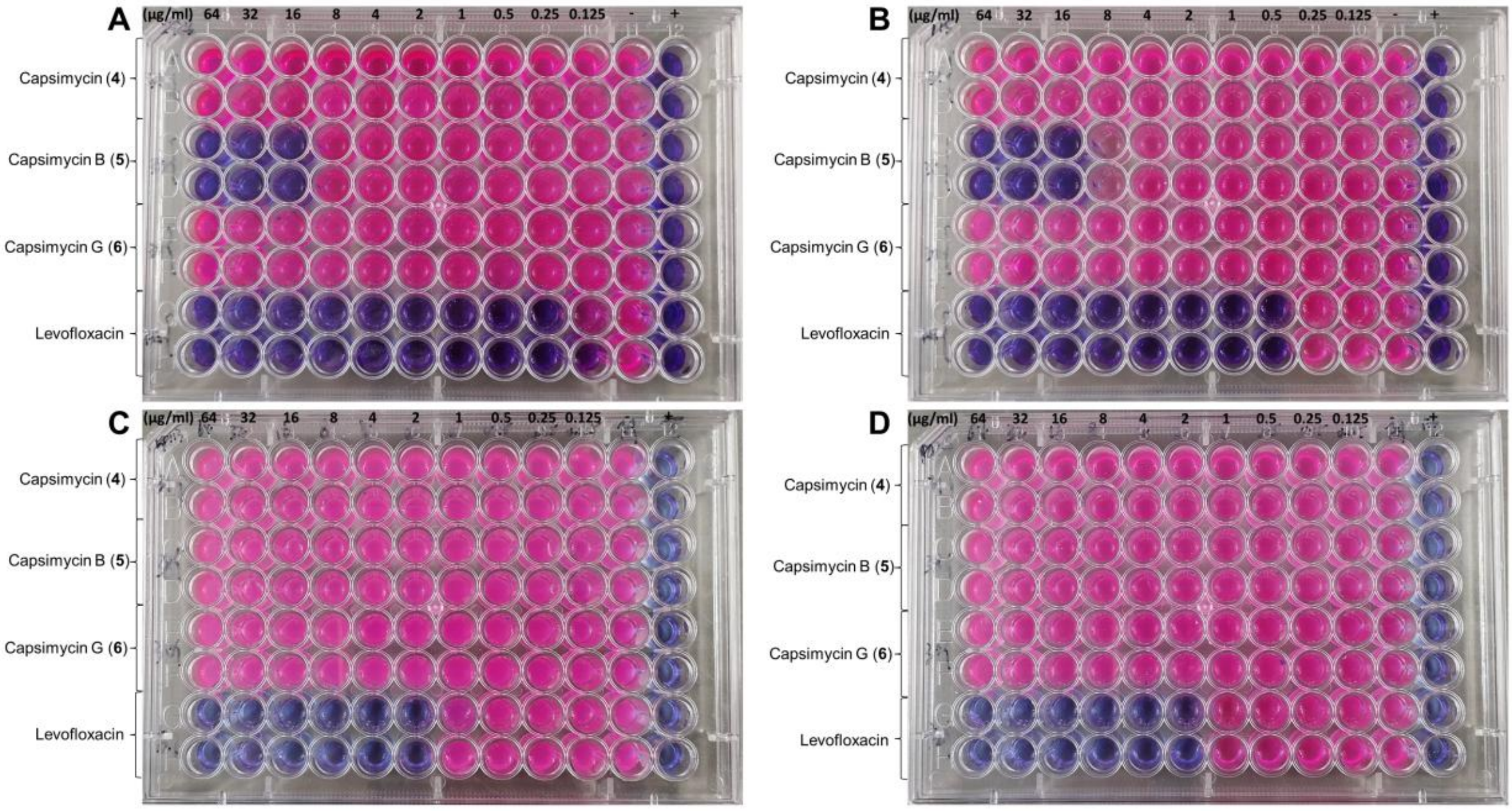

References

1. Xu XT, Deng XY, Chen J, Liang QM, Zhang K, Li DL, et al. Synthesis and biological evaluation of coumarin derivatives as α-glucosidase inhibitors. Eur J Med Chem. 2020;189:112013. https://doi.org/10.1016/j.ejmech.2019.112013.

2. Trott O, Olson AJ. AutoDock Vina: improving the speed and accuracy of docking with a new scoring function, efficient optimization, and multithreading. J Comput Chem. 2010;31:455–61. https://doi.org/10.1002/jcc.21334.

3. Sanner MF. Python: a programming language for software integration and development. J Mol Graph Model. 1999;17:57–61.

4. Morris GM, Huey R, Lindstrom W, Sanner MF, Belew RK, Goodsell DS, et al. AutoDock4 and AutoDockTools4: Automated docking with selective receptor flexibility. J Comput Chem. 2009;30:2785–91. https://doi.org/10.1002/jcc.21256.

5. Salomon-Ferrer R, Case DA, Walker RC. An overview of the Amber biomolecular simulation package. WIREs Comput Mol Sci. 2013;3:198–210. https://doi.org/10.1002/wcms.1121.

6. Sagui C, Darden TA. Molecular dynamics simulations of biomolecules: long-range electrostatic effects. Annu Rev Biophys Biomol Struct. 1999;28:155–79. https://doi.org/10.1146/annurev.biophys.28.1.155.

7. Kräutler V, Van Gunsteren WF, Hünenberger PH. A fast SHAKE algorithm to solve distance constraint equations for small molecules in molecular dynamics simulations. J Comput Chem. 2001;22:501–8. https://doi.org/10.1002/1096-987X(20010415)22:5%253C501::AID-JCC1021%253E3.0.CO;2-V.

8. Larini L, Mannella R, Leporini D. Langevin stabilization of molecular-dynamics simulations of polymers by means of quasisymplectic algorithms. J Chem Phys. 2007;126. https://doi.org/10.1063/1.2464095.

9. Hou T, Wang J, Li Y, Wang W. Assessing the performance of the MM/PBSA and MM/GBSA methods: 1. The accuracy of binding free energy calculations based on molecular dynamics simulations. J Chem Inf Model. 2011;51:69–82. https://doi.org/10.1021/ci100275a.

10. Chen Y, Zheng Y, Fong P, Mao S, Wang Q. The application of the MM/GBSA method in the binding pose prediction of FGFR inhibitors. Phys Chem Chem Phys. 2020;22:9656–63. https://doi.org/10.1039/D0CP00831A.

11. Genheden S, Ryde U. The MM/PBSA and MM/GBSA methods to estimate ligand-binding affinities. Expert Opin Drug Discov. 2015;10:449–61. https://doi.org/10.1517/17460441.2015.1032936.

12. Rastelli G, Rio AD, Degliesposti G, Sgobba M. Fast and accurate predictions of binding free energies using MM-PBSA and MM-GBSA. J Comput Chem. 2010;31:797–810. https://doi.org/10.1002/jcc.21372.

13. Nguyen H, Roe DR, Simmerling C. Improved generalized born solvent model parameters for protein simulations. J Chem Theory Comput. 2013;9:2020–34. https://doi.org/10.1021/ct3010485.

14. Weiser J, Shenkin PS, Still WC. Approximate atomic surfaces from linear combinations of pairwise overlaps (LCPO). J Comput Chem. 1999;20:217–30. https://doi.org/10.1002/(SICI)1096-987X(19990130)20:2%253C217::AID-JCC4%253E3.0.CO;2-A.
