# Supplementary material for: Capsimycin derivatives from Streptomyces sp. PH9007 as α‑glucosidase inhibitors and insights into their biosynthetic gene cluster
Source: BMC Genomics. 2026 Aug 18;27:944. doi: 10.1186/s12864-026-13269-7 (PMC13761111; doi:10.1186/s12864-026-13269-7)
Supplement: Supplementary file 2 [file 12864_2026_13269_MOESM2_ESM.pdf]

Supporting Information II

Capsimycin Derivatives from *Streptomyces* sp. PH9007 as α-Glucosidase Inhibitors and Insights into Their Biosynthetic Gene Cluster

Zhong Wang [1, a], Guangling Wu [1, a], Jian Pan [1, c], Runze Sun [a], Shun Cao [a], Xiaoyan Jin [d], Yani Zhong [a], Yuhan Chen [a], Lizi Cao [a], Xinlu Chen [a], Bin Feng [b, *], Xueshuang Huang [a, *], and Hong Pu [a, *]

[a]*Hunan Provincial Key Laboratory for Synthetic Biology of Traditional Chinese Medicine, School of Pharmaceutical Sciences, Hunan University of Medicine, Huaihua, Hunan, 418000, China;*

[b]*Huaihua Hospital of Traditional Chinese Medicine, Huaihua 418000, China;*

[c]*College of Pharmacy, Hunan Vocational College of Science and Technology, Changsha 410004, China;*

[d]*College of Pharmacy, Xinjiang Medical University, Urumchi, Xinjiang, 830011, China*

*__Corresponding author.__

E-mail address: puhong@hnmu.edu.cn (H. Pu), xueshuanghuang@126.com (X. S. Huang),

txz-1221@163.com (B. Feng)

[1] These authors contributed equally to this work

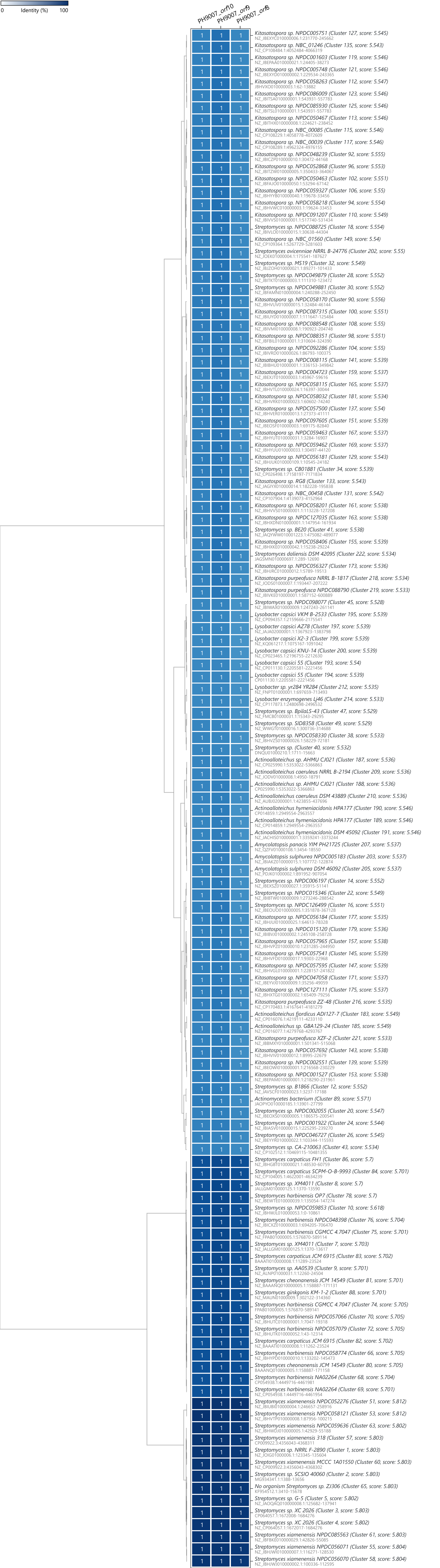
0
Identity (%)
100
PH9007_orf10
PH9007_orf9
PH9007_orf8
Kitasatospora sp. NPDC005751 (Cluster 127, score: 5.545)
Kitasatospora sp. NBC_01246 (Cluster 135, score: 5.543)
Kitasatospora sp. NPDC001603 (Cluster 119, score: 5.546)
Kitasatospora sp. NPDC005748 (Cluster 121, score: 5.546)
Kitasatospora sp. NPDC058263 (Cluster 112, score: 5.547)
Kitasatospora sp. NPDC086009 (Cluster 123, score: 5.546)
Kitasatospora sp. NPDC085930 (Cluster 125, score: 5.546)
Kitasatospora sp. NPDC050467 (Cluster 113, score: 5.546)
Kitasatospora sp. NBC_00085 (Cluster 115, score: 5.546)
Kitasatospora sp. NBC_00039 (Cluster 117, score: 5.546)
Kitasatospora sp. NPDC048239 (Cluster 92, score: 5.555)
Kitasatospora sp. NPDC052868 (Cluster 96, score: 5.553)
Kitasatospora sp. NPDC050463 (Cluster 102, score: 5.551)
Kitasatospora sp. NPDC059327 (Cluster 106, score: 5.55)
Kitasatospora sp. NPDC058218 (Cluster 94, score: 5.554)
Kitasatospora sp. NPDC091207 (Cluster 110, score: 5.549)
Streptomyces sp. NPDC088725 (Cluster 18, score: 5.554)
Kitasatospora sp. NBC_01560 (Cluster 149, score: 5.54)
Streptomyces avicenniae NRRL B-24776 (Cluster 202, score: 5.55)
Streptomyces sp. MS19 (Cluster 32, score: 5.549)
Streptomyces sp. NPDC049879 (Cluster 28, score: 5.552)
Streptomyces sp. NPDC049881 (Cluster 30, score: 5.552)
Kitasatospora sp. NPDC058170 (Cluster 90, score: 5.556)
Kitasatospora sp. NPDC087315 (Cluster 100, score: 5.551)
Kitasatospora sp. NPDC088548 (Cluster 108, score: 5.55)
Kitasatospora sp. NPDC088351 (Cluster 98, score: 5.551)
Kitasatospora sp. NPDC092286 (Cluster 104, score: 5.55)
Kitasatospora sp. NPDC008115 (Cluster 141, score: 5.539)
Kitasatospora sp. NPDC004723 (Cluster 159, score: 5.537)
Kitasatospora sp. NPDC058115 (Cluster 165, score: 5.537)
Kitasatospora sp. NPDC058032 (Cluster 181, score: 5.534)
Kitasatospora sp. NPDC057500 (Cluster 137, score: 5.54)
Kitasatospora sp. NPDC097605 (Cluster 151, score: 5.539)
Kitasatospora sp. NPDC059463 (Cluster 167, score: 5.537)
Kitasatospora sp. NPDC059462 (Cluster 169, score: 5.537)
Kitasatospora sp. NPDC056181 (Cluster 129, score: 5.543)
Streptomyces sp. CB01881 (Cluster 34, score: 5.539)
Kitasatospora sp. RG8 (Cluster 133, score: 5.543)
Kitasatospora sp. NBC_00458 (Cluster 131, score: 5.542)
Kitasatospora sp. NPDC058201 (Cluster 161, score: 5.538)
Kitasatospora sp. NPDC127035 (Cluster 163, score: 5.538)
Streptomyces sp. BE20 (Cluster 41, score: 5.538)
Kitasatospora sp. NPDC058406 (Cluster 155, score: 5.539)
Streptomyces daliensis DSM 42095 (Cluster 222, score: 5.534)
Kitasatospora sp. NPDC056327 (Cluster 173, score: 5.536)
Kitasatospora purpeofusca NRRL B-1817 (Cluster 218, score: 5.534)
Kitasatospora purpeofusca NPDC088790 (Cluster 219, score: 5.533)
Streptomyces sp. NPDC098077 (Cluster 45, score: 5.528)
Lysobacter capsici VKM B-2533 (Cluster 195, score: 5.539)
Lysobacter capsici AZ78 (Cluster 197, score: 5.539)
Lysobacter capsici X2-3 (Cluster 199, score: 5.539)
Lysobacter capsici KNU-14 (Cluster 200, score: 5.539)
Lysobacter capsici 55 (Cluster 193, score: 5.54)
Lysobacter capsici 55 (Cluster 194, score: 5.539)
Lysobacter sp. yr284 YR284 (Cluster 212, score: 5.535)
Lysobacter enzymogenes Lj46 (Cluster 214, score: 5.533)
Streptomyces sp. BpilaLS-43 (Cluster 47, score: 5.529)
Streptomyces sp. SID8358 (Cluster 49, score: 5.529)
Streptomyces sp. NPDC058330 (Cluster 38, score: 5.533)
Streptomyces sp. (Cluster 40, score: 5.532)
Actinoalloteichus sp. AHMU CJ021 (Cluster 187, score: 5.536)
Actinoalloteichus caeruleus NRRL B-2194 (Cluster 209, score: 5.536)
Actinoalloteichus sp. AHMU CJ021 (Cluster 188, score: 5.536)
Actinoalloteichus caeruleus DSM 43889 (Cluster 210, score: 5.536)
Actinoalloteichus hymeniacidonis HPA177 (Cluster 190, score: 5.546)
Actinoalloteichus hymeniacidonis HPA177 (Cluster 189, score: 5.546)
Actinoalloteichus hymeniacidonis DSM 45092 (Cluster 191, score: 5.546)
Amycolatopsis panacis YIM PH21725 (Cluster 207, score: 5.537)
Amycolatopsis sulphurea NPDC005183 (Cluster 203, score: 5.537)
Amycolatopsis sulphurea DSM 46092 (Cluster 205, score: 5.537)
Streptomyces sp. NPDC006197 (Cluster 14, score: 5.552)
Streptomyces sp. NPDC015346 (Cluster 22, score: 5.549)
Streptomyces sp. NPDC126499 (Cluster 16, score: 5.551)
Kitasatospora sp. NPDC056184 (Cluster 177, score: 5.535)
Kitasatospora sp. NPDC015120 (Cluster 179, score: 5.536)
Kitasatospora sp. NPDC057965 (Cluster 157, score: 5.538)
Kitasatospora sp. NPDC057541 (Cluster 145, score: 5.539)
Kitasatospora sp. NPDC057595 (Cluster 147, score: 5.539)
Kitasatospora sp. NPDC047058 (Cluster 171, score: 5.537)
Kitasatospora sp. NPDC127111 (Cluster 175, score: 5.537)
Kitasatospora purpeofusca ZZ-48 (Cluster 216, score: 5.535)
Actinoalloteichus fjordicus ADI127-7 (Cluster 183, score: 5.549)
Actinoalloteichus sp. GBA129-24 (Cluster 185, score: 5.549)
Kitasatospora purpeofusca XZF-2 (Cluster 221, score: 5.533)
Kitasatospora sp. NPDC057692 (Cluster 143, score: 5.538)
Kitasatospora sp. NPDC002551 (Cluster 139, score: 5.539)
Kitasatospora sp. NPDC001527 (Cluster 153, score: 5.538)
Streptomyces sp. B1866 (Cluster 12, score: 5.552)
Actinomycetes bacterium (Cluster 89, score: 5.571)
Streptomyces sp. NPDC002055 (Cluster 20, score: 5.547)
Streptomyces sp. NPDC001922 (Cluster 24, score: 5.544)
Streptomyces sp. NPDC046727 (Cluster 26, score: 5.545)
Streptomyces sp. CA-210063 (Cluster 43, score: 5.534)
Streptomyces carpaticus FH1 (Cluster 86, score: 5.7)
Streptomyces carpaticus SCPM-O-B-9993 (Cluster 84, score: 5.701)
Streptomyces sp. XM4011 (Cluster 8, score: 5.7)
Streptomyces harbinensis OP7 (Cluster 78, score: 5.7)
Streptomyces sp. NPDC059853 (Cluster 10, score: 5.618)
Streptomyces harbinensis NPDC048398 (Cluster 76, score: 5.704)
Streptomyces harbinensis CGMCC 4.7047 (Cluster 75, score: 5.701)
Streptomyces sp. XM4011 (Cluster 7, score: 5.703)
Streptomyces carpaticus JCM 6915 (Cluster 83, score: 5.702)
Streptomyces sp. AA0539 (Cluster 9, score: 5.701)
Streptomyces cheonanensis JCM 14549 (Cluster 81, score: 5.701)
Streptomyces ginkgonis KM-1-2 (Cluster 88, score: 5.701)
Streptomyces harbinensis CGMCC 4.7047 (Cluster 74, score: 5.705)
Streptomyces harbinensis NPDC057066 (Cluster 70, score: 5.705)
Streptomyces harbinensis NPDC057079 (Cluster 72, score: 5.705)
Streptomyces carpaticus JCM 6915 (Cluster 82, score: 5.702)
Streptomyces harbinensis NPDC058774 (Cluster 66, score: 5.705)
Streptomyces cheonanensis JCM 14549 (Cluster 80, score: 5.705)
Streptomyces harbinensis NA02264 (Cluster 68, score: 5.704)
Streptomyces harbinensis NA02264 (Cluster 69, score: 5.701)
Streptomyces xiamenensis NPDC052276 (Cluster 51, score: 5.812)
Streptomyces xiamenensis NPDC058121 (Cluster 53, score: 5.812)
Streptomyces xiamenensis NPDC059636 (Cluster 63, score: 5.802)
Streptomyces xiamenensis 318 (Cluster 57, score: 5.803)
Streptomyces sp. NRRL F-2890 (Cluster 1, score: 5.803)
Streptomyces xiamenensis MCCC 1A01550 (Cluster 60, score: 5.803)
Streptomyces sp. SCSIO 40060 (Cluster 2, score: 5.803)
No organism Streptomyces sp. ZJ306 (Cluster 65, score: 5.803)
Streptomyces sp. G-5 (Cluster 5, score: 5.802)
Streptomyces sp. XC 2026 (Cluster 3, score: 5.803)
Streptomyces sp. XC 2026 (Cluster 4, score: 5.802)
Streptomyces xiamenensis NPDC085563 (Cluster 61, score: 5.803)
Streptomyces xiamenensis NPDC056071 (Cluster 55, score: 5.804)
Streptomyces xiamenensis NPDC056070 (Cluster 58, score: 5.804)
